# Polarization Engineering in Vinylene‐Linked COFs Toward Efficient Neuromorphic Computing

**DOI:** 10.1002/advs.76639

**Published:** 2026-07-14

**Authors:** Hao Wang, Lei Zhao, Haoyuan Yao, Qiongshan Zhang, Fuzhen Xuan, Bin Zhang

**Affiliations:** ^1^ Key Laboratory for Advanced Materials School of Chemistry and Molecular Engineering East China University of Science and Technology Shanghai China; ^2^ Shanghai Key Laboratory of Intelligent Sensing and Detection Institute of Intelligent Sensing and Instruments East China University of Science and Technology Shanghai China

**Keywords:** covalent organic framework, neuromorphic computing, polarization engineering, speech emotion recognition

## Abstract

Functional delocalized covalent organic frameworks (COFs) show promise as active layers for the development of memristor switches and multistate functionalities. However, their potential is limited by poor electron mobility and suboptimal reaction kinetics. This study explores polarization engineering to modify the local electronic structure of the biaryl units within vinylene‐linked COFs. By implementing a conformation‐locking strategy that strategically enhances N^+^ polarization and the redox activity of the framework, this design not only decreases the interfacial Schottky barrier but also facilitates efficient electron transfer, thereby promoting the delocalization of framework electrons, which modulates the conductive state in organic memristors. Furthermore, the polarization of ions, amplified by pyridine nitrogen nuclei and robust interlayer stacking, ensures effective counterion deintercalation and migration. The constructed Al/COF‐DIBPY/Au memristor exhibits a high analog on‐off current ratio (I_max_/I_min_ = 15.7) under a 0.5 V drive. Utilizing these properties, the device shows great potential in performing speech emotion recognition tasks. When integrated into a convolutional neural network, it achieves a learning recognition accuracy of up to 95% in these tasks. This research offers novel insights into the construction of high‐efficiency neuromorphic computing devices based on polarization‐modulated COFs.

## Graphical abstract

A vinyl‐linked covalent organic framework (COF‐DIBPY) was synthesized on a conductive substrate for memristor applications. Redox reactions and bromide migration enable low‐voltage (0.5V) conductance switching with high analog on/off ratio (15.7). The device was further integrated into a speech emotion recognition system for acoustic simulation and human–computer emotional interaction.

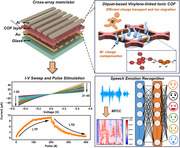

## 1 Introduction

Organic memristors represent a pivotal hardware solution for addressing the energy efficiency limitations of von Neumann architectures in next‐generation computing [1, 2]. Their prominence is primarily attributed to their integrated storage‐computing capabilities, along with advantages such as ultra‐low power consumption and high‐density integration. However, practical applications encounter significant challenges, including elevated operating voltages and restricted analog switching ratios, which arise from inadequate electron migration and ionic conduction [3, 4, 5, 6, 7, 8, 9]. Moreover, the intrinsic noise of these devices adversely affects the signal‐to‐noise ratio during synaptic weight updates, leading to a dispersed conductive state distribution and diminished state retention capabilities [10, 11, 12, 13, 14, 15]. These limitations significantly hinder the advancement of organic memristors in high‐precision, brain‐inspired computing applications.

2D covalent organic frameworks (2D COFs) are a notable class of crystalline porous materials characterized by ordered channel structures, high planarity, and diverse topological architectures [16, 17, 18, 19, 20, 21, 22]. They demonstrate significant potential for applications in the field of organic optoelectronics. To overcome the challenges posed by high charge recombination rates and limited mobility within COFs [23, 24, 25, 26, 27], substantial improvements in performance can be realized through strategies such as band engineering, interface regulation, and the construction of long‐range ordered charge transport networks. For example, recent studies have shown that enhancing the delocalization effect of imines in protonated COF materials markedly increases device stability and cycling endurance [28]. Furthermore, modulating asymmetric dipole interactions to promote anti‐parallel columnar stacking between crystalline layers can facilitate gradual charge transfer, thereby enhancing the multi‐level switching behavior of memristors [2]. Consequently, the development of advanced functional COFs is essential for improving the stability and tunability of conductive states in memristors.

A critical strategy for modulating the spatial distribution of charges and defects, along with the localized behavior of organic materials, is the engineering of their intrinsic polarization through targeted approaches [29, 30, 31, 32]. This modulation enables the effective control of charge carrier transport properties under external fields. One common method involves the introduction of heteroatoms or highly polar functional groups, such as nitro, cyan, or fluorine atoms, into the molecular framework. These heteroatoms or functional groups exert significant σ‐inductive or π‐conjugative effects, thereby enhancing the permanent dipole moment of the molecule [33, 34, 35, 36]. Another notable approach is the construction of zwitterionic systems, which generate strong intrinsic electric fields within molecules. This design significantly increases the asymmetry of electron cloud distribution. The accompanying counterions help maintain the electrical neutrality and structural stability of the system by dynamically compensating for charge imbalances through mechanisms induced by external fields. Furthermore, reducing the spatial or electronic structural symmetry of molecules or materials alleviates constraints on their internal charge distribution [37], promoting non‐equilibrium charge separation.

**FIGURE 1:**
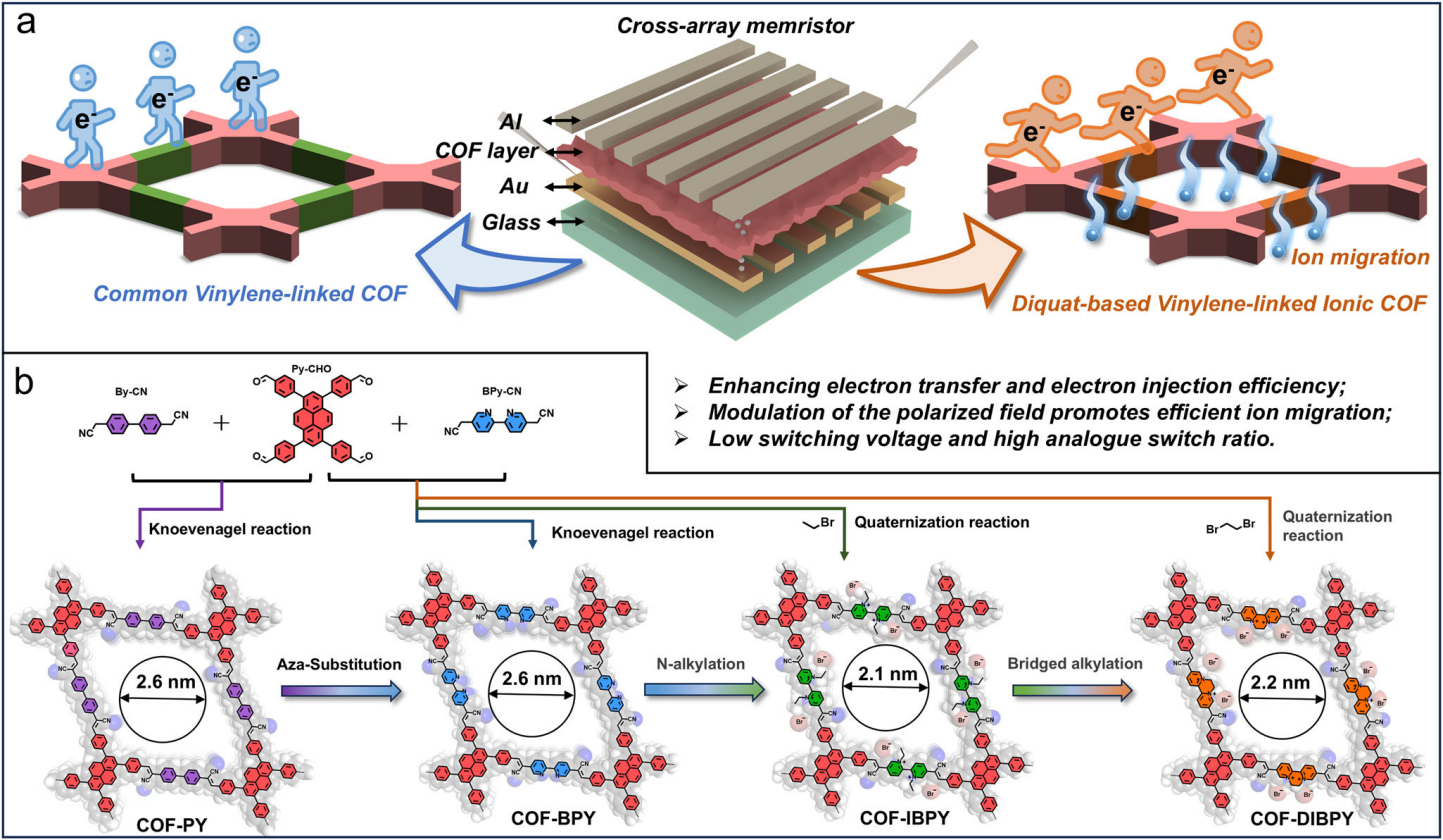
Charge transfer behavior of functional COFs and material design. (a) Schematic of charge transferring in COFs, (b) Methods for synthesizing COF‐PY, COF‐BPY, COF‐IBPY and COF‐DIBPY.

Given the significant impact of the N‐core polarization effect and the conformational stabilization provided by bipyridine on electron delocalization within the framework, this study employed a polarization modulation strategy to reconfigure the local electronic structure of the biaryl unit. This approach enhances the conductance regulation capability of the memristor under low excitation voltages. Both experimental and theoretical results confirm that the COF incorporating diquat (COF‐DIBPY) exhibits exceptional memristive performance, attributable to enhanced electron transfer efficiency and favorable ion migration behavior, as illustrated in Figure 1a. Specifically, bridging alkylation amplifies the polarization effect of the N^+^ species by limiting conformational twisting, which effectively lowers the interfacial Schottky barrier at the electrode‐active layer interface. Simultaneously, the diquat unit acts as an efficient redox center, facilitating precise modulation of electron delocalization throughout the framework. Moreover, tight *π*–*π* stacking, enabled by conformational planarization, along with heightened ion polarization between counterions, further promotes efficient bromide ion deintercalation and migration. The fabricated Al/COF‐DIBPY/Au device demonstrates high‐performance analog switching (I_max_/I_min_ = 15.7) at a low driving voltage of 0.5 V. When integrated into a convolutional neural network, the device achieves 95% accuracy in speech emotion recognition tasks.

## 2 Results and Discussion

We used 4,4',4'',4''‐[1,8‐dihydronaphthalene‐1,3,6,8‐tetrayl] tetraphthalaldehyde (Py‐CHO) and either 2,2'‐[1,1'‐biphenyl]‐4,4'‐diyl diacetone nitrile (By‐CN) or 2,2'‐[2,2'‐bipyridine]‐5,5'‐diyl diethyl dicyanide (BPy‐CN) as the backbone for constructing vinylene‐linked COFs. The reaction was conducted in 1,2‐dichlorobenzene/n‐butanol, employing Cs_2_CO_3_ as the catalyst. Subsequently, COF‐BPY was ionized to obtain COF‐IBPY or COF‐DIBPY (Figure 1b) [38, 39]. Notably, the synthesis of COF‐DIBPY was performed under Brønsted acidic conditions to enhance the conversion rate of trans‐2,2'‐dipyridine to cis‐2,2'‐dipyridine (Figure S1). By adjusting the protonation concentration within the system, the target compound in the skeleton was nearly quaternized into diquat (The quaternization yield for COF‐IBPY was 73.5%, and 70.2% for COF‐DIBPY, see Figure S2 and Table S1).

**FIGURE 2:**
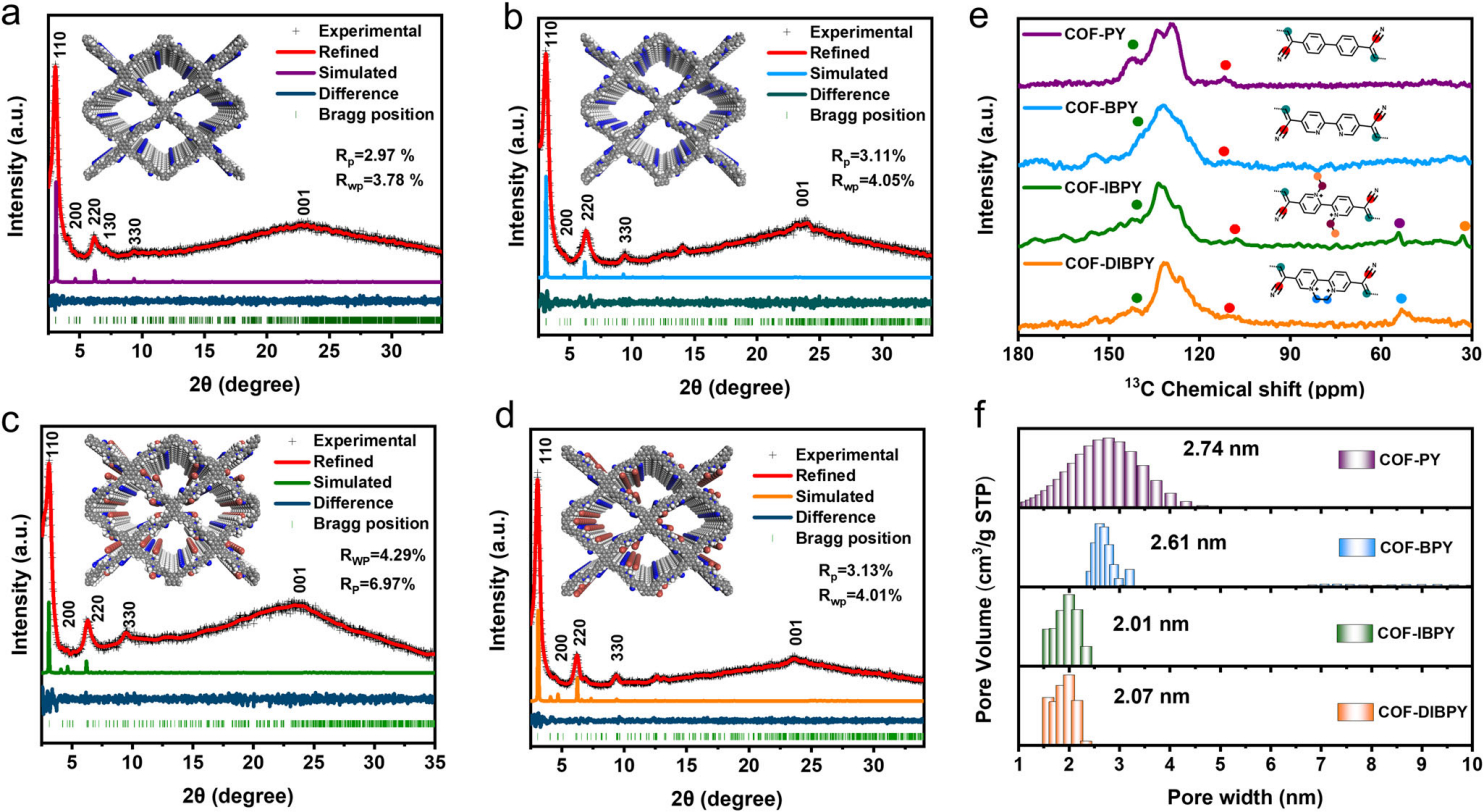
Synthesis and characterization of COFs. X‐ray diffraction patterns of (a) COF‐PY, (b) COF‐BPY, (c) COF‐IBPY and (d) COF‐DIBPY, (e) Solid‐state ^13^C CP/MAS NMR spectra and (f) Pore‐size distributions of four COFs.

First, the X‐ray diffraction (XRD) patterns (Figure 2a–d) show the crystallinity of the four polymers, with diffraction peaks at 2θ values of 3.1°, 6.3°, and 9.3° corresponding to the (110), (220), and (330) crystal planes, respectively. Pawley refinement using the reflex module suggested a crystal cell with a P1 space group characterized by an eclipsed AA stacking mode (specific cell parameters and stacking patterns are detailed in Table S2 and Figure S3). The chemical structures of the four COFs were elucidated through Fourier transform infrared (FTIR) spectroscopy and solid‐state ^13^C nuclear magnetic resonance (ssNMR) spectroscopy. The FTIR spectra (Figure S4) indicated the successful occurrence of the Knoevenagel reaction, evidenced by the decrease in C═O stretching vibrational peaks at 1700 cm^−1^ and the formation of C═C stretching vibrational peaks at 1650 cm^−1^. A weak characteristic peak at 2207 cm^−1^ can be assigned to the C≡N stretching of the vinyl cyan group. Additionally, FTIR analysis revealed the appearance of methylene (–CH_2_‐) stretching at 2926 and 2873 cm^−1^ (Figure S4b), indicating successful quaternization of the pyridine nitrogen in both COF‐IBPY and COF‐DIBPY. The distinct chemical states of various carbon elements within the COFs were analyzed via solid‐state carbon spectroscopy (Figure 2e). Chemical shifts at 110 ppm and 141 ppm correspond to carbon signals for the cyan group and vinyl moieties situated near the pyrene nucleus, respectively. In COF‐IBPY, shifts at 34 ppm and 52 ppm are attributed to the carbon signals of the methyl and methylene groups of the pendant alkyl chain following quaternization. In contrast, the absence of the carbon signal at 34 ppm in COF‐DIBPY alongside the persistence of the signal at 52 ppm further supports the cyclization of 2,2'‐bipyridine into a diquat structure.

The thermal stability of the four COFs was assessed through thermogravimetric analysis (TGA), where weight losses observed around 200°C and 400°C were attributed to the loss of the quaternized chains and framework decomposition, respectively (Figure S5). Brunauer‐Emmett‐Teller (BET) analysis (Figure 2f and Figure S6) indicated that quaternization significantly contracted the pore structure of the COFs. Specifically, compared to COF‐PY and COF‐BPY, which exhibited specific surface areas of 482 and 600 m^2^/g and pore sizes of 2.61 and 2.74 nm, the quaternized products, COF‐IBPY and COF‐DIBPY, displayed markedly reduced specific surface areas (211 and 225 m^2^/g) and pore sizes (2.01 and 2.07 nm). This decrease is attributed to the occupation of the material's effective pore space by grafted alkyl molecules and accompanying bromide counterions. To further investigate the grain structure of the four materials, we conducted field emission scanning electron microscopy (FESEM) and high‐resolution transmission electron microscopy (HRTEM) analyses (Figures S7–S10). After applying Fourier filtering to selected‐area images, we measured the corresponding lattice spacing to be approximately 0.3 nm, which aligns with the expected interlayer spacing. Additionally, the FESEM images (Figures S11 and S12) demonstrated that the prepared films exhibited remarkable continuity and compactness. Energy‐dispersive X‐ray spectroscopy (EDS), in conjunction with elemental imaging, confirmed a uniform elemental distribution both before and after quaternization. Atomic force microscopy (AFM) analysis (Figures S11e–h and S12e–h) indicated film thicknesses in the range of several hundred nanometers.

**FIGURE 3:**
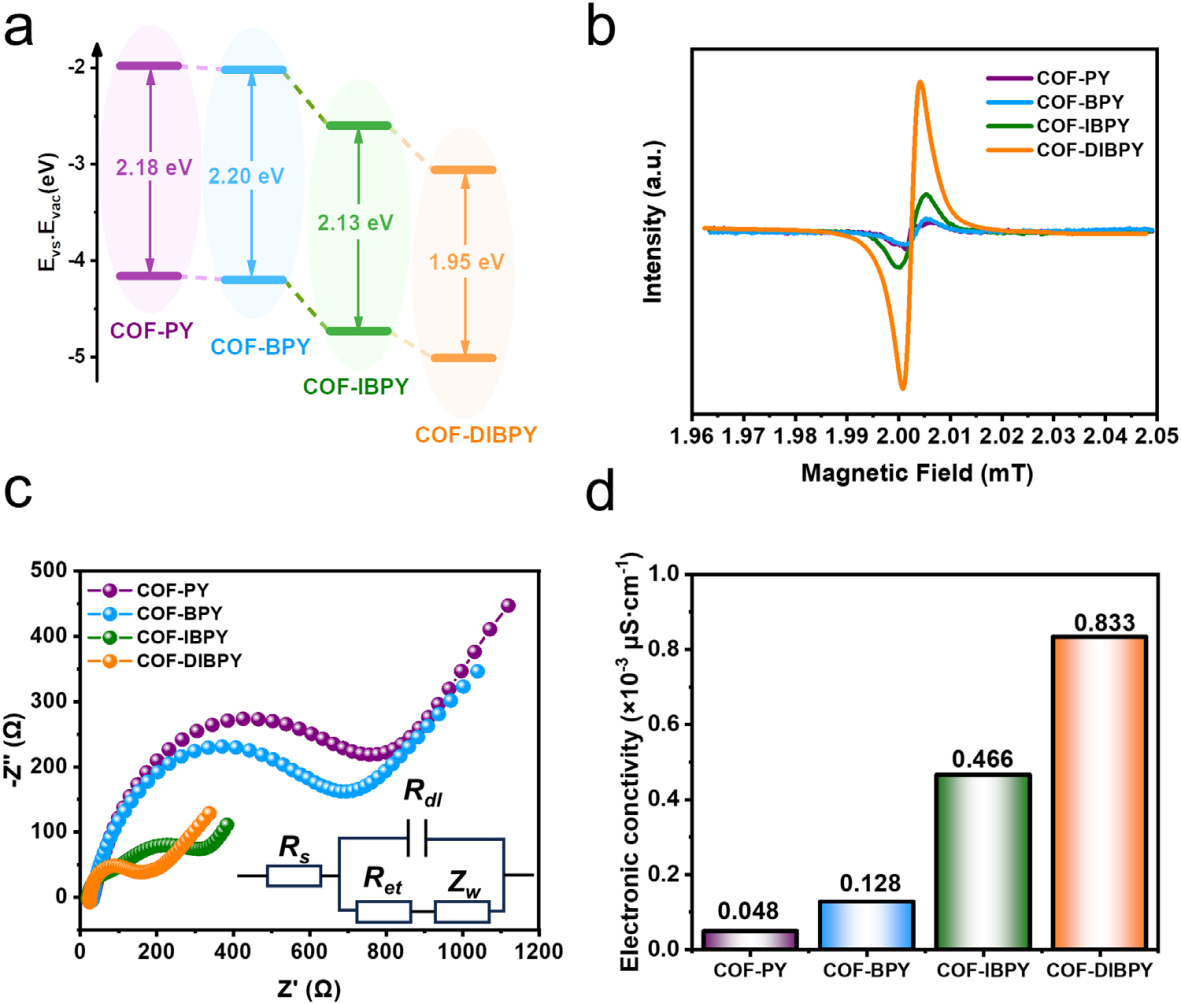
Photoelectrochemical properties. (a) Energy level diagram, (b) EPR measurement, (c) EIS spectra, and (d) Electronic conductivities of four COFs.

The optical properties of COF‐PY, COF‐BPY, COF‐IBPY, and COF‐DIBPY were examined using ultraviolet‐visible (UV) spectroscopy, revealing that all four COFs respond to visible light (Figure S13a). Compared to COF‐PY, COF‐BPY exhibited a red shift of approximately 19 nm, which increased significantly after quaternization (COF‐IBPY: ∼40 nm; COF‐DIBPY: ∼103 nm). Consistent with this, the corresponding fluorescence emission spectra also exhibit a pronounced red‐shift trend (Figure S14). The optical bandgap values were calculated using the Tau‐c method (Figure S13b), while the Valence Band Maximum (VBM), Conduction Band Minimum (CBM) and Fermi level(E_F_)for the four COFs were derived from ultraviolet photoelectron spectroscopy (UPS) results (Figure S15). As illustrated in Figure 3a, the bandgap width decreases sequentially from COF‐PY to COF‐DIBPY. This reduction in bandgap facilitates both intramolecular and intermolecular charge transfer (ICT) processes within the donor‐acceptor (D‐A) system [40]. And the lowering of the E_F_ effectively reduces the difference in work functions between the Al electrode and the COF (1.1 eV→0.37 eV, see Table S3), thereby reducing the bending of the interfacial band structure [41], lowering the contact resistance. To assess the electrical properties of the COFs, cyclic voltammetry (CV) was performed (Figure S16). The reduction potentials of COF‐PY, COF‐BPY, COF‐IBPY, and COF‐DIBPY exhibited a gradual decrease, indicating lower reaction kinetic barriers due to increased rate constants for ion migration and reduction reactions [42]. Notably, COF‐DIBPY displayed reversible redox peaks associated with the two‐electron redox process of diquat.

Electron paramagnetic resonance (EPR) spectroscopy (Figure 3b) demonstrated the presence of free radicals in all four COFs, with COF‐DIBPY having the highest concentration. This finding suggests that the free radicals generated by the diquat units significantly contribute to the observed effects. The charge transfer kinetics of the materials were analyzed using electrochemical impedance spectroscopy (EIS) (Figure 3c). The results indicated that ionization modification of the COFs more effectively reduces interfacial charge transfer resistance. This improvement in interfacial kinetics results in lower operating voltages for the device. To further explore the influence of these intrinsic properties on conductivity, we measured electronic conductivity using the four‐probe method (Figure 3d). As anticipated, the electronic conductivities of COF‐IBPY and COF‐DIBPY were found to be 3.6 times and 6.5 times higher than that of the parent COF, respectively. This demonstrates that the optoelectronic properties of ionic COFs significantly surpass those of their non‐ionic counterparts. The integration of redox‐active diquat units, through synergistic reversible electron transfer effects, leads to narrower bandgaps, reduced resistivity, and enhanced conductivity.

**FIGURE 4:**
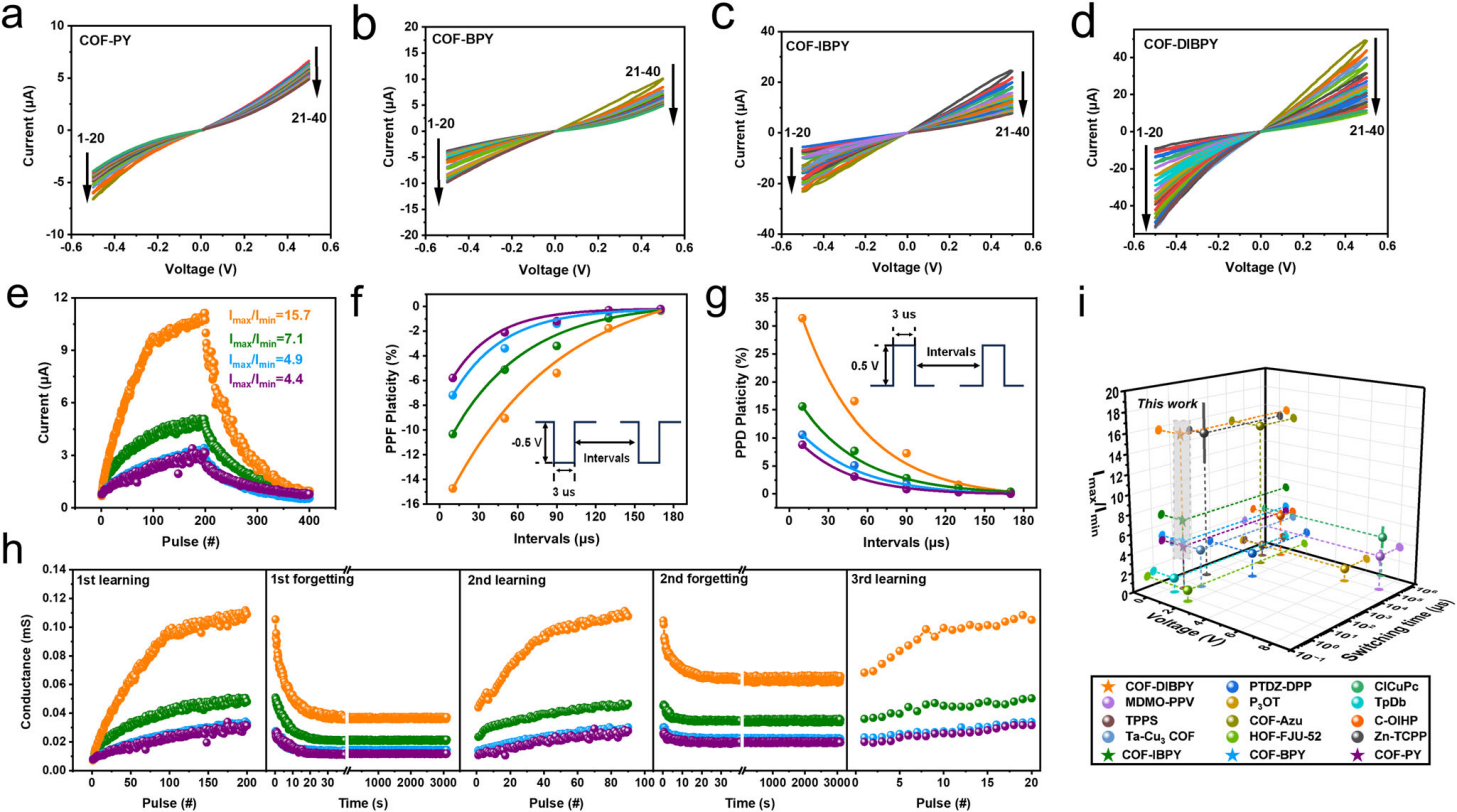
Memristor conductance switching behavior and synaptic simulation. The current‐voltage characteristic curves of (a) Al/COF‐PY/Au, (b) Al/COF‐BPY/Au, (c) Al/COF‐IBPY/Au, and (d) Al/COF‐DIBPY/Au memristors. (e) Simulation of long‐term potentiation (LTP) and long‐term depression (LTD) processes in synapses at a read voltage of 0.1 V. Plasticity versus pulse interval characteristics of the device. The fitting of (f) PPF and (g) PPD are according to the equation of y = y_0_ + A exp (−x/t). (h) Demonstration of the ‘learning‐forgetting‐relearning’ process for four devices, with a pulse amplitude of 0.5 V, a pulse width of 3 µs, and a device monitoring current reading of 0.1 V. (i) Comparison with the performance of organic memristors recently reported. (The analogue switch ratio is estimated based on the LTP values from relevant literature).

The electrical performance of the four COF‐based devices was assessed at room temperature using a Keithley 4200 semiconductor parameter analyzer. As illustrated in Figure 4a–d, all four devices exhibited typical hysteresis loops during voltage scans ranging from 0 to ±0.5 V. After 20 I‐V cycles, we observed distinct changes in the current responses of the devices. Specifically, for the Al/COF‐PY/Au device, the current gradually increased from approximately 4–7 µA during negative scans. The Al/COF‐BPY/Au device showed a current increase from around 4–10 µA, while the Al/COF‐IBPY/Au device's current rose from about 5–25 µA. In the case of the Al/COF‐DIBPY/Au device, the current increased significantly from approximately 10–46 µA. Notably, in the forward scans, the current for all devices returned to their initial states (Figure S17). By monitoring the stability of each conductance state after cycling the devices, we obtained 13, 20, 28, and 31 distinct and stable conductance states, each lasting up to 1000 s (Figure S18), based on the four devices. To further validate these findings, we performed independent measurements of 50 bistable cycles for each device type. The statistical analysis of the switch‐on and switch‐off voltage distributions is presented in Figure S19. The average turn‐on voltages were −0.92, −0.90, −0.80, and −0.79 V, while the turn‐off voltages were 2.24, 2.21, 2.11, and 1.89 V, respectively. Additionally, the off‐state current for each device was measured, revealing that the Al/COF‐DIBPY/Au structure exhibited the largest variance in off‐state current (Figure S20). This high dispersion suggests the presence of numerous metastable states within the device, which significantly enhances the efficiency of neuromorphic computing.

To investigate the synaptic plasticity of the four devices, we subjected the memristors to continuous stimulation under conditions of ±0.5 V pulse amplitude and 3 µs pulse width, as illustrated in Figure S21. Negative electrical stimulation induced varying degrees of change in the threshold current response across the devices, in alignment with the results obtained from the I‐V cycle scan. Repeated electrical stimulation had the most significant impact on the peak current of the Al/COF‐DIBPY/Au device, followed by the Al/COF‐IBPY/Au device, while the Al/COF‐PY/Au device exhibited the least sensitivity to stimulation. Furthermore, positive electrical stimulation was able to restore the peak current to its initial value. By assessing the conductivity of each device during the pulse application, we effectively simulated long‐term potentiation (LTP) and long‐term depression (LTD) in synaptic behavior. As demonstrated in Figure 4e, under 200 alternating negative and positive pulses, the maximum current ratios for the four devices were recorded at 15.7, 7.1, 4.9, and 4.4, respectively. The conductance states demonstrated high reproducibility under repeated pulses, with the noise level for each current measurement maintained below 2% (Figure S22). We also evaluated the frequency‐dependent and voltage‐dependent plasticity of the devices. As shown in Figures S23 and S24, higher frequencies and increased voltages favored positive (and negative) changes in the conductive state, suggesting a positive correlation between frequency, amplitude, and neuronal connectivity. Additionally, paired‐pulse facilitation (PPF) and paired‐pulse depression (PPD) illustrate the dynamic regulatory capacity of synapses in response to input pulses, significantly affecting postsynaptic current levels by modulating the temporal intervals between consecutive pulses. We quantified the functional indices of PPF and PPD using the following formula:

$$\mathrm{PPF}/\mathrm{PPD}\;=\frac{A_{2}-A_{1}}{A_{1}}\times 100\%$$

where A_1_ and A_2_ represent the currents measured in response to the initial and subsequent stimuli, respectively. As illustrated in Figure 4f,g, an increase in the interval time between pulses leads to a decrease in the index variable, indicating a negative correlation between pulse interval duration and changes in postsynaptic current. Among the four devices examined, a reduction in interval time significantly affects the modulation depth of the conductance state in the Al/COF‐DIBPY/Au device, while the Al/COF‐PY/Au device shows less dependence on pulse interval. Additionally, we assessed the learning and forgetting capabilities of these devices to explore their potential for neuromorphic computing. Under identical pulse conditions, the Al/COF‐DIBPY/Au device demonstrates the greatest weight modulation due to its unique conductance characteristics (Figure S25). This enhanced weight modulation significantly improves the reliability, robustness, and noise tolerance of the signal readout. Moreover, repeated learning allows these devices to achieve maximum learning capacity with fewer pulse stimuli (Figure 4h), and their extensive capacity range suggests excellent anti‐forgetting capabilities, contributing to more stable long‐term information retention. Figure 4i summarizes the analog switching performance of organic memristors (Table S4). Under the guidance of our polarized design, the Al/COF‐DIBPY/Au device demonstrates a well‐balanced combination of high analog switching ratio, low operating voltage, and fast switching response.

**FIGURE 5:**
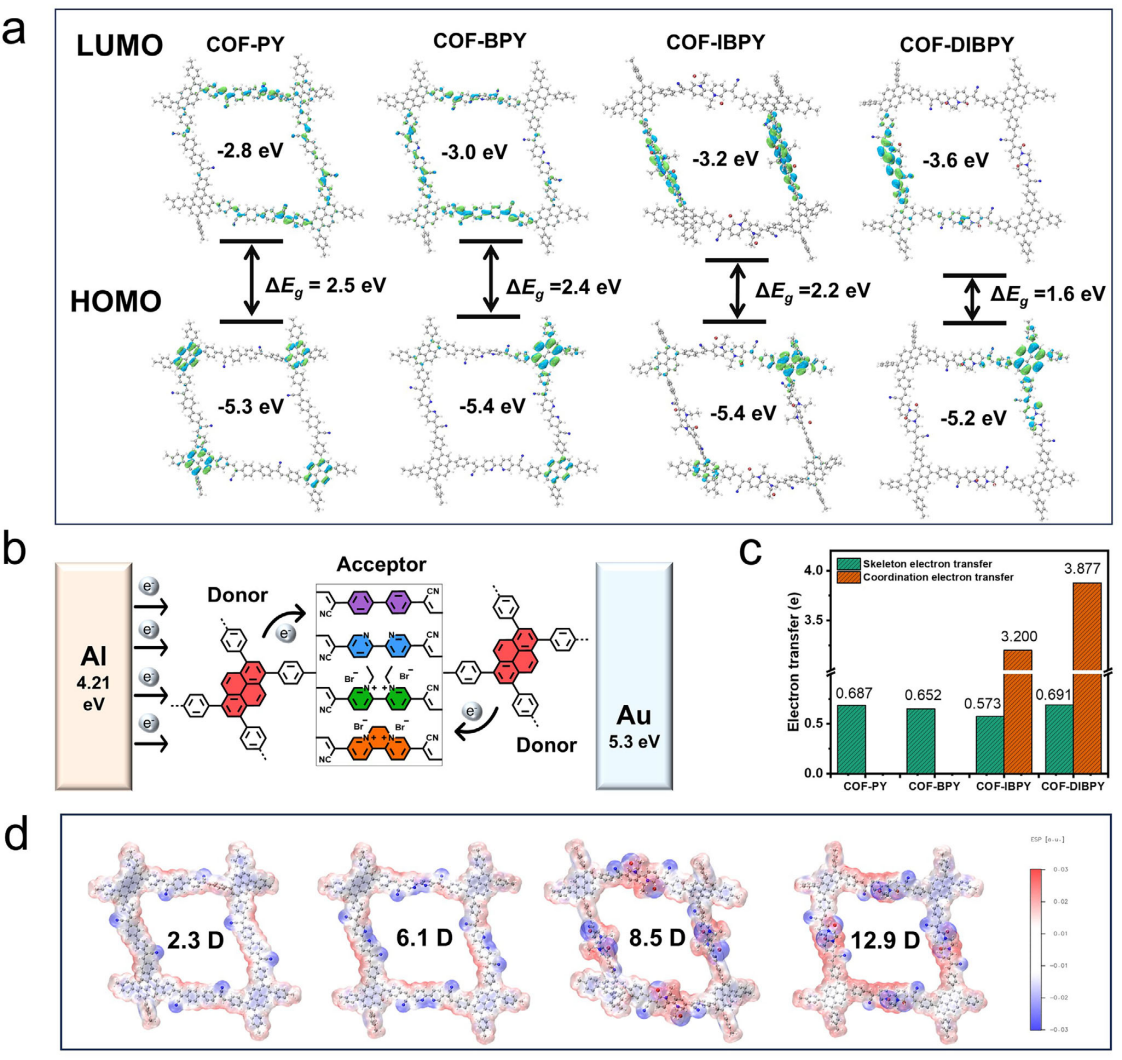
Charge transfer enhancement mechanism. (a) HOMO‐LUMO energy gaps of organic macrocycles based on COF‐PY, COF‐BPY, COF‐IBPY, and COF‐DIBPY. (b) Interface‐based structural engineering to enhance memristor behavior mechanisms. (c) The charge analysis of the skeleton reflects the amount of charge transfer in the COF structure. (d) Electrostatic potential distribution and dipole moment magnitude of four types of COF rings.

To elucidate the potential mechanism of charge transfer in memristors across the four COF structures, we employed representative functionalized repeating unit model and performed density functional theory (DFT) calculations to elucidate the effect of local polarization on the electronic properties of different systems. The analysis of frontier orbitals of the macrocyclic molecule reveals that the highest occupied molecular orbital (HOMO) energy is predominantly localized within the pyrene skeleton (Figure 5a), while the lowest unoccupied molecular orbital (LUMO) energy is primarily associated with the bi‐aryl structural units. Notably, functional ionization significantly lowers the LUMO energy level of the framework [43, 44], which enhances efficient electron capture under external electric fields and substantially reduces the randomness in conductive filament formation [45]. To minimize the possible influence of a native Al_2_O_3_ interfacial layer, all devices were fabricated under identical deposition conditions. The work function of thermally evaporated aluminum was measured to be ∼4.21 eV (Figure S26), and the expected energy alignment phenomenon at the Al/COF interface was further investigated. As illustrated in Figure 5b, the interface potential barriers for COF‐PY, COF‐BPY, COF‐IBPY, and COF‐DIBPY show a decreasing trend, indicating an increased efficiency of electron injection from the Al electrode into the COF layer under negative voltage drive. To quantitatively assess the electron transfer capability of the COFs, we performed atomic dipole moment‐corrected Hirshfeld population analysis on the COF rings (Figure 5c and Figure S27). After ring closure, the COF‐DIBPY structure exhibits a charge value of 0.691 e on the skeletal pyrene, which is significantly higher than that observed in the other three COFs, indicating a more pronounced electron transfer from pyrene toward diquat. Furthermore, functional ionization illustrates that the COF‐DIBPY ring can donate more electrons to the bromine atom, demonstrating enhanced ionic polarization [46]. Additionally, the electrostatic potential (ESP) analysis of the rings revealed dipole moments of 2.3 D for COF‐PY, 6.1 D for COF‐BPY, and 8.5 D and 12.9 D for COF‐IBPY and COF‐DIBPY, respectively (Figure 5d). These results suggest that the vinylene‐linked COF based on diquat exhibits superior charge separation capability [47, 48]. The strong separation of electron and hole stabilized trap levels provides multiple metastable energy states for the quantitative injection and extraction of charge, thereby dynamically ensuring broad‐range, highly stable switching and maintenance between different conduction states.

**FIGURE 6:**
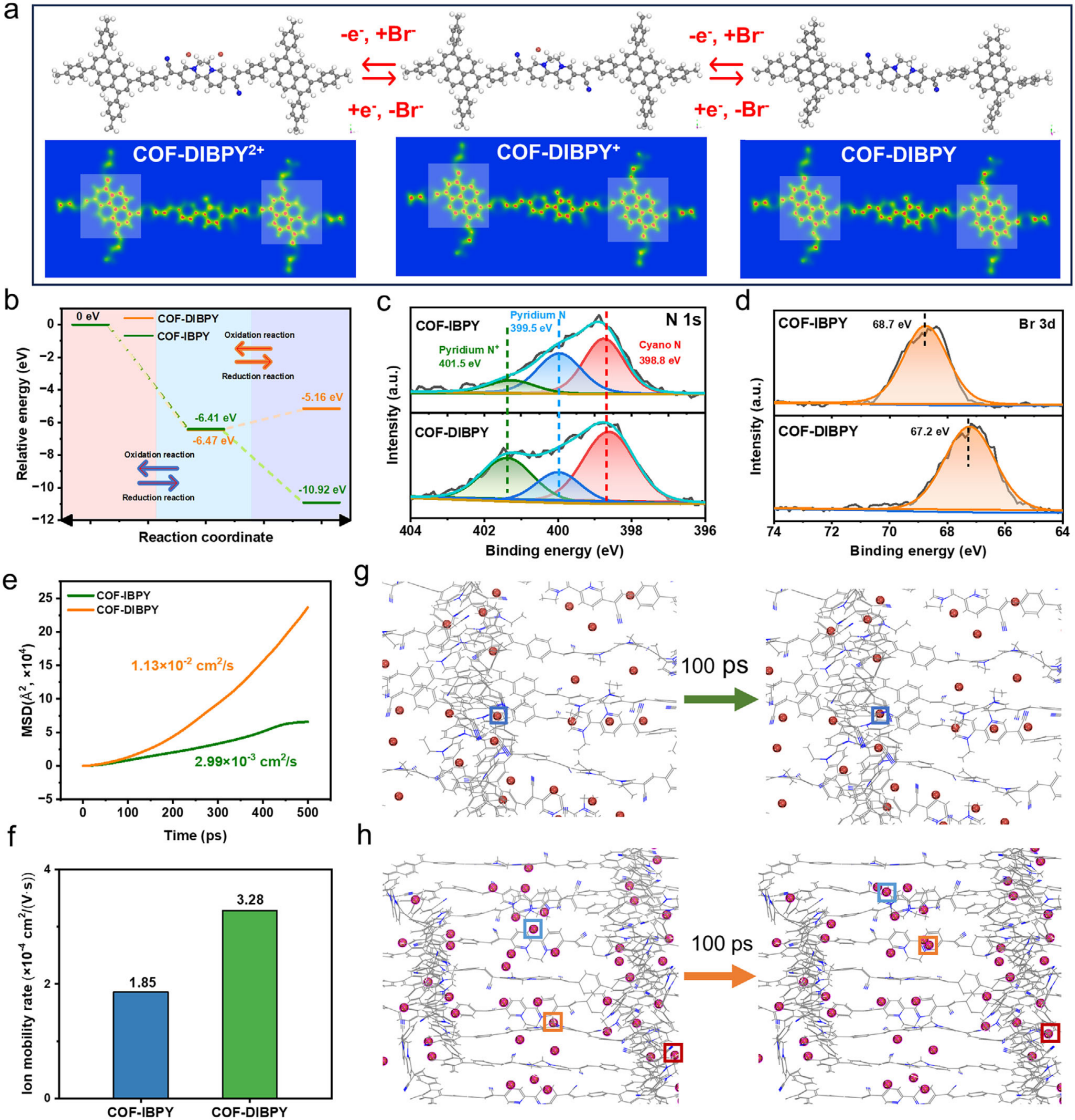
Redox reactions and polarization ions in ionic COFs. Structural evolution during redox reactions based on COF‐DIBPY repeating units, (a) corresponding electron density maps, and (b) relative energy changes. XPS spectra of (c) N 1s orbitals and (d) Br 3d orbitals of COF‐IBPY and COF‐DIBPY. The curve depicting the (e) mean square displacement (MSD) and (f) electro‐migration rate of bromide ions within the COF as a function of simulation time. Simulated snapshots captured at 0 (left) and 100 (right) ps respectively, illustrate the bromide ion transfer process within the simulated unit cells of (g) COF‐IBPY and (h) COF‐DIBPY.

Investigating the synergistic mechanisms of redox activity and ion migration kinetics in viologens enhances our understanding of their multi‐conductive state behavior in Al/COF‐DIBPY/Au devices. To examine the electronic structural origins of conductive evolution during redox processes, we first constructed a simplified model system based on D‐A‐D repeating units [49, 50]. Electron density mapping indicates that the molecular framework of COF‐DIBPY demonstrates significantly enhanced electron delocalization following reduction compared to COF‐IBPY (Figure 6a and Figure S28). Moreover, COF‐DIBPY exhibits a smaller thermodynamic energy difference between its reduced and initial states, suggesting greater efficiency in electron capture and release within a polarizing field (Figure 6b). X‐ray photoelectron spectroscopy (XPS) analysis was performed on both ionic COFs (Figure S29), with binding energies calibrated using the C 1s peak (284.6 eV) of contaminant carbon as a reference. Analysis of the N 1s orbitals (Figure 6c) shows that the binding energies at 399.5 eV (pyridine N) and 401.5 eV (pyridine N^+^) in COF‐DIBPY exhibit lower relative abundances compared to COF‐IBPY, indicating that COF‐ DIBPY develops stronger localized charges through ionic polarization [51, 52]. Furthermore, in comparison to COF‐IBPY, the Br 3d XPS peak in COF‐DIBPY displays a significant negative shift (from 68.7 to 67.2 eV; Figure 6d), further indicating reduced delocalization of Br^−^ outer electrons toward the vacant orbitals of the skeleton. Zeta potential analysis (Figure S30) further reveals alterations in surface potential induced by the presence of highly localized charges. Moreover, the restriction of conformational twists in the conjugated aromatic rings of COF‐DIBPY, which arises from bridged alkylation, facilitates tighter *π*–*π* stacking (Figure S31), significantly enhancing interlayer electronic transition conduction.

To elucidate the differences in polarized ons, we employed molecular dynamics simulations. Because ion hopping is a long‐range collective process, we constructed a 3 × 3 × 3 supercell to represent the periodic potential field, thereby accounting for both the hopping barrier and lattice deformation energy. The 2D number density distribution (Figure S32) indicates that counterions were initially anchored by the framework. For the examined bromide ion species, the time‐dependent mean square displacement (MSD) is defined as follows: $\mathrm{MSD}(t)=\frac{1}{N}\;\sum_{i=1}^{N}\left\langle {|r_{i}\left(t_{0}+t\right)-r_{i}\left(t_{0}\right)|}^{2}\right\rangle$where N is the number of diffusing ions, r_i_(t) represents the cartesian coordinate of ion i at time t, and the angular brackets denote an average over all possible time origins t_0_ along the equilibrium segment of the trajectory. As shown in Figure 6e, under the Fickian regime, their diffusion coefficients D can be calculated as 1.13 × 10^−^^2^ cm^2^/s and 2.99 × 10^−3^ cm^2^/s, respectively. Concurrently, the corresponding ion migration rates governed by the electric field can be estimated as 1.85 × 10^−4^ cm^2^/(V·s) and 3.28 × 10^−4^ cm^2^/(V·s), respectively (Figure 6f and Figure S33; further details are provided in the Supporting Equations). Simulation snapshots further reveal that after 100 ps, a greater number of bromide ions undergo deintercalation and migration within COF‐DIBPY (Figure 6g,h). This is attributed to enhanced *π*–*π* stacking interactions and stronger ion polarization, establishing efficient polarization pathways with the polar main chain. Moreover, COF‐DIBPY achieves interaction energies comparable to COF‐IBPY at larger counterion distances (Figures S34 and S35). Consequently, its ionic migration inevitably occurs at a lower gradient of interaction strength [52], consistent with the observed faster bromide ion migration.

**FIGURE 7:**
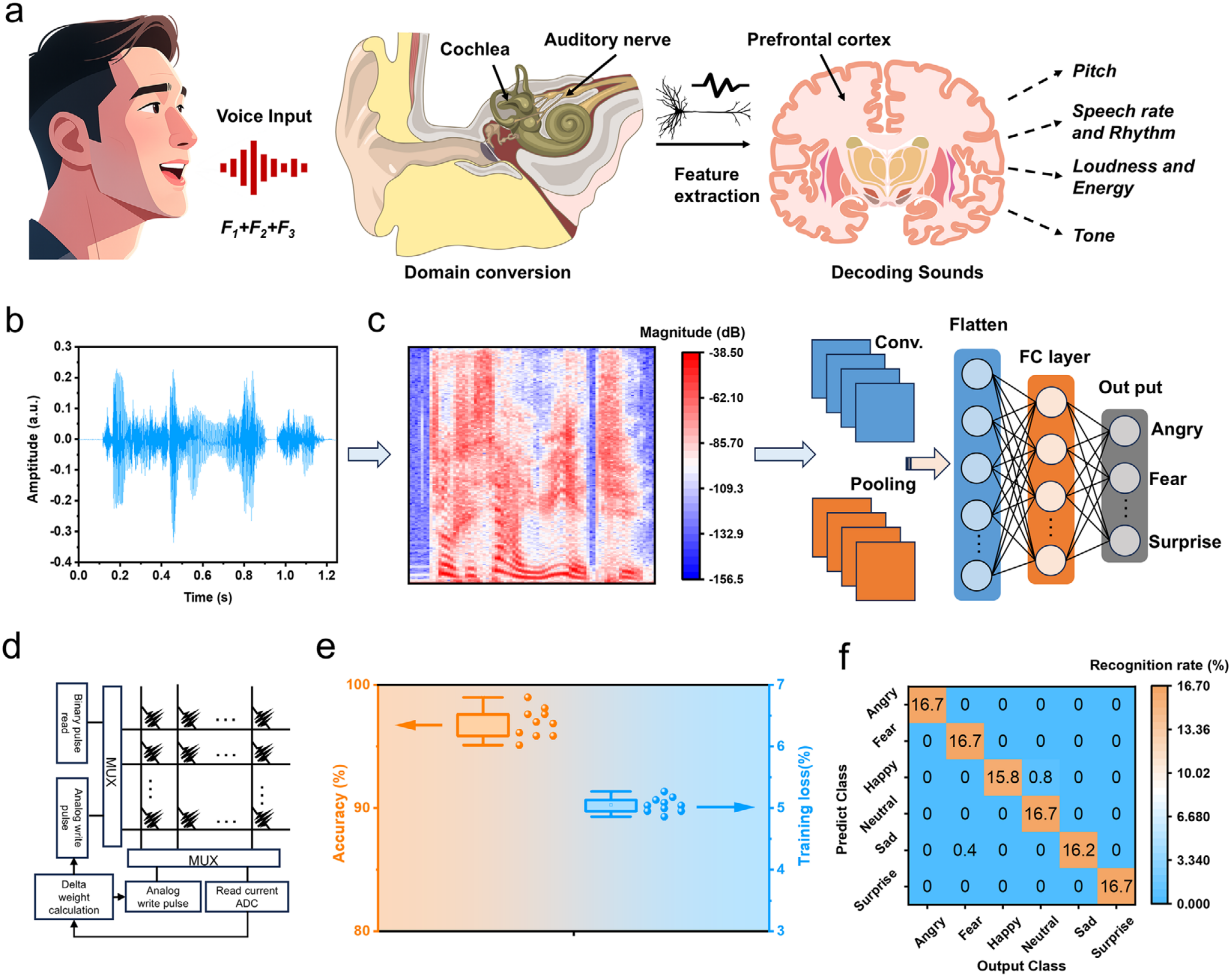
Memristor‐based speech emotion recognition system. (a) Schematic diagram of the human auditory system: Includes the conversion of acoustic signals in the ear canal into voltage‐encoded spectral information, as well as higher‐order memory, integration, and decision‐making functions mediated by the hippocampus and prefrontal cortex. (b) Spectrogram of speech signals with distinctive features. By converting the audio signal from a time‐domain spectrogram into a feature representation. (c) The Mel‐frequency cepstral coefficients (MFCCs) spectrogram and subsequently feeding this information into a convolutional neural network (CNN) to identify the corresponding speech emotion. (d) Hardware implementation circuit diagram. (e) Accuracy and loss rate changes for Al/COF‐DIBPY/Au devices after 300 training sessions. (f) Confusion matrix after 300 training sessions.

The human auditory system processes information by first decomposing sound signals into specific frequency components via hair cells on the cochlear basilar membrane [53, 54]. These signals are then transmitted as electrical impulses through the auditory nerve to the primary auditory cortex, where acoustic features such as high‐frequency tones, speech rate and rhythm, loudness and energy, and timbre are analyzed and decoded. Subsequently, the hippocampus and prefrontal cortex integrate memory and contextual information to facilitate decision‐making (Figure 7a).

Inspired by this process, we constructed a convolutional neural network (CNN) based on Al/COF/Au synapse devices and integrated it into a speech emotion recognition system. First, an emotionally rich audio signal is converted into a spectrogram via an analog‐to‐digital converter (Figure 7b). To address the feature transformation from the time domain to the frequency domain, we employ Mel‐frequency cepstral coefficient (MFCC) feature extraction. As shown in Figure 7c, the short‐time Fourier transform converts the time‐domain waveform into a frequency‐domain spectrum [55, 56]. This spectrum then passes through a Mel filter bank to simulate the nonlinear scaling of human auditory perception. Following logarithmic compression, a discrete cosine transform (DCT) extracts low‐dimensional cepstral coefficients, which are fed into the CNN for speech emotion recognition. The simulated CNN architecture comprises three convolutional layers, three pooling layers, one fully connected layer, and one SoftMax layer [57]. Nonlinear expression capability (ReLU) is incorporated to learn complex patterns, while standardizing intermediate layer outputs stabilizes and accelerates training (Table S5). The database contains speech segments representing six emotions: anger, fear, happiness, neutral, sadness, and surprise (Figure S36), with 40 samples per emotion category. Figure 7d illustrates a memristor array serving as neural network synapse weight units, with device LTP and LTD curves extracted. Input values are encoded via pulse count and normalized conductance values (see Supporting Equation and Figure S37). We evaluated hardware performance through device recognition capability. As shown in Figure 7e, the Al/COF‐DIBPY/Au device achieved 95% recognition accuracy after 300 training cycles, with only a 5% accuracy decay rate. By learning and recognizing 240 speech spectra (Figure 7f), the confusion matrix results demonstrate that the target device achieves high recognition accuracy for different types of emotional speech across 300 cycles. These results indicate the broad application prospects of Al/COF‐DIBPY/Au devices in brain‐inspired acoustic processing and human‐machine emotional interaction.

## 3 Conclusion

In summary, we have reconstructed the local electronic structure of the biaryl groups in the vinylene‐linked COF through a polarization engineering strategy to enhance the performance of memristors. Both theoretical and experimental results demonstrate that the COF embedded with diquat exhibits exceptional memristive characteristics, which can be attributed to improved electron transfer efficiency and enhanced ion migration rates. Specifically, bridged alkylation enhances local polarization effects, thereby improving interfacial carrier injection efficiency on one hand, while redox reactions centered on diquat promote efficient electron transfer and precisely regulate framework delocalization on the other, ensuring favorable conductance modulation in the memristor. Furthermore, stronger ion polarization effects between counter ions facilitate efficient bromide ion deintercalation and migration. The fabricated Al/COF‐DIBPY/Au device demonstrates a high analog switching ratio (Imax/Imin = 15.7) at a driving voltage of 0.5 V. When integrated into a convolutional neural network, the device achieves a learning recognition accuracy of up to 95% in speech emotion recognition tasks. This work establishes a new paradigm for high‐performance memristors by implementing local electronic modulation of COFs through a polarization engineering strategy.

## Author Contributions

**Lei Zhao**: software, data curation, investigation, validation, formal analysis. **Haoyuan Yao**: data curation, investigation, formal analysis. **Hao Wang**: methodology, software, data curation, investigation, formal analysis, visualization, writing – original draft, resources. **Qiongshan Zhang**: validation, data curation, investigation, software. **Fuzhen Xuan**: supervision, funding acquisition, project administration. **Bin Zhang**: conceptualization, methodology, supervision, funding acquisition, project administration, writing – review and editing.

## Conflicts of Interest

The authors declare no conflicts of interest.

## Supporting information




**Supporting File**: advs76639‐sup‐0001‐SuppMat.docx.

## Data Availability

The data that support the findings of this study are available from the corresponding author upon reasonable request.
