# Supplementary material for: Polarization Engineering in Vinylene‐Linked COFs Toward Efficient Neuromorphic Computing
Source: Adv Sci (Weinh). 2026 Jul 14:e76639. Online ahead of print. doi: 10.1002/advs.76639 (PMC13367213; doi:10.1002/advs.76639)
Supplement: Supplementary file 1 — Supporting File: advs76639‐sup‐0001‐SuppMat.docx. [file ADVS-9999-e76639-s001.docx]

**Supporting Information**

**Polarization Engineering in Vinylene-Linked COFs toward Efficient Neuromorphic Computing**

Hao Wang^1^, Lei Zhao^1^, Haoyuan Yao^1^, Qiongshan Zhang^1^, Fuzhen Xuan^2^, Bin Zhang*^1,2^

^1^Key Laboratory for Advanced Materials and Joint International Research Laboratory of Precision Chemistry and Molecular Engineering, School of Chemistry and Molecular Engineering, East China University of Science and Technology, Shanghai 200237, China

^2^Shanghai Key Laboratory of Intelligent Sensing and Detection, Institute of Intelligent Sensing and Instruments, East China University of Science and Technology, Shanghai 200237, China

All correspondence and request for materials should be addressed to: zhangbin@ecust.edu.cn (Prof. Bin Zhang).

**Materials and methods**

**Materials**

1,3,6,8-tetrabromopyrene (Py-Br), 5,5'-dimethyl-2,2'-dipyridyl, 1,2-dichlorobenzene (o-DCB), N-bromosuccinimide and azobisisobutyronitrile were purchased from Meyer, Shanghai. 4-formylphenylboronic acid, potassium carbonate (K_2_CO_3_) and Pd (PPh_3_)_4_ were purchased from Titan Shanghai. 1,4-dioxane, acetone, methanol, cesium carbonate (Cs_2_CO_3_), n-butanol, dimethyl sulfoxide and carbon tetrachloride (CCl_4_) were purchased from Adamas. ^[1,2]^ The synthesis of 4,4',4'',4'''-(pyrene-1,3,6,8-tetrayl) tetra-benzaldehyde and 2,2'-([2,2'-bipyridine]-5,5'-diyl) di-acetonitrile were based on previously reported procedures. All the operations were performed under purified argon. All chemicals were used without further purification. All electrical measurements were performed on a Keithley 4200 semiconductor parameter analyzer in ambient condition without any device encapsulation. Patterned glass substrate (5 mm × 10 mm) was pre-cleaned sequentially with water, acetone and 2-propanol in an ultrasonic bath for 15 mins, and then treated with oxygen plasma. Al top electrodes were thermally deposited on the surface of active layer through a shadow mask at 10^-7^ Torr via E-beam evaporation.

**Characterization methods**

X-ray diffraction (XRD) patterns were recorded on D/max 2500pc (Rigaku) for Cu Ka radiation (1.5406 Å). Fourier transform infrared (FTIR) spectra were recorded by Spectrum 100 spectrophotometer (Perkin Elmer, Inc., USA). Nuclear magnetic resonance (NMR) spectra were recorded on a Bruker 400 spectrometer at a resonance frequency of 400 MHz for ^1^H in deuterated solution with a tetramethylsilane (TMS) as a reference for the chemical shifts. ^13^C cross polarization/magic angle spinning solid-state nuclear magnetic resonance (CP/MAS ssNMR) experiments were performed on a Bruker AVANCE III 400 WB spectrometer operating at 100.62 MHz for ^13^C using a double resonance 4 mm MAS NMR probe and a sample spinning rate of 10 kHz. The cross-polarization time was 1 ms. The chemical shifts were referenced with adamantane. Thermogravimetric analysis of COFs powder samples was performed on a Diamond TG/DTA/DSC Thermal Analyzer System (Perkin-Elmer, USA) with heating rate of 10 °C min-1 to 800 °C N_2_ atmosphere. Field emission scanning electron microscopy (FE-SEM) images were achieved on 500-300000/Gemini SEM 500. Transmission electron microscopy (TEM) images were obtained using a JEOL-2100 (JEOL Ltd., Japan) TEM system operated at 200 kV. Electrochemical impedance spectroscopy (EIS) measurements were measured using CHI660E electrochemical work-station. Tests were performed over a frequency range of 0.1 MHz to 0.1 Hz with an oscillating voltage of 10 mV. X-ray photoelectron spectroscopy (XPS) measurements were carried out on a Kratos AXIS HSi spectrometer with a monochromatized Al Kr X-ray source (1486.6 eV photons) at a constant dwell time of 100 ms and pass energy of 40 eV. The anode voltage and current were set at 15 kV and 10 mA, respectively. The pressure in the analysis chamber was maintained at 5×10^-8^ Torr or lower during each measurement. Cyclic voltammetry (CV) measurements were conducted on the CHI660E electrochemical workstation using the standard three-electrode system, comprising a gold electrode coated with COF as the working electrode, a platinum plate as the counter electrode, and a silver/silver chloride electrode as the reference electrode. Fluorescence spectroscopy experiments were performed on the FLS1000 fluorescence spectrometer. Photoelectron Spectrometer (UPS) spectrum was recorded on an Esca lab 250Xi (Thermo Scientific). Gas sorption analyses were performed on Quantachrome Instruments surface area and pore analyzer. The samples were degassed at 120 ºC for 24 h before measurement. The bromide ion content was determined by Inductively Coupled Plasma Mass Spectrometry (ICP-MS:Agilent 7800). The Brunauer-Emmett-Teller (BET) surface area and total pore volume were calculated from the N_2_ sorption isotherms at 77 K. Atomic Force Microscope (AFM) images were obtained using Bruker Dimension Icon.

### **Computational Details**

Theoretical calculations pertaining to molecular frontier orbitals were conducted utilizing the ORCA software package, version 6.0.0. Geometry optimization was carried out within the framework of density functional theory (DFT) employing the BLYP functional. To accurately account for non-covalent interactions, particularly dispersion effects, Grimme's D3 empirical correction with Becke-Johnson damping (D3BJ) was incorporated. The def2-SVP basis set was selected for its balance between computational efficiency and accuracy, accompanied by the corresponding auxiliary def2/J basis set to accelerate the resolution of the Coulomb integral. Subsequent to optimization, single-point energy calculations were performed at the optimized geometries to refine the electronic energy description. These calculations utilized the hybrid B3LYP functional in conjunction with the def2-SVP basis set and its def2/J auxiliary counterpart, providing an improved description of molecular orbital energies and properties. For detailed analysis of the electronic structure, atom-dipole corrected Hirshfeld (ADCH) population analysis was employed to compute atomic charges, offering a robust metric for charge distribution. Furthermore, the electrostatic potential (ESP) was mapped onto the electron density isosurface to visualize regions of electrophilic and nucleophilic reactivity. All post-processing of electronic structure data, including charge analysis and ESP visualization, was performed using the Multiwfn program, with graphical representations generated using the VMD software package.

The computation of interaction energies, Gibbs free energies, and charge density distributions was carried out using the Dmol3 module within the Materials Studio software environment. Within the framework of density functional theory (DFT) under the generalized gradient approximation (GGA), the BLYP functional was employed, offering an optimal balance between computational tractability and predictive accuracy. This approach was specifically applied to investigate the interaction mechanisms between ions and the organic frameworks, reliably providing energetically optimized geometries and revealing key trends in interfacial interactions. The interaction energy between Br- and the COF ring can be defined as

$$\text{E}_{\text{inter}}\text{=}\text{E}_{\text{sum}}\text{-}\text{E}_{\text{1}}\text{-}\text{E}_{\text{2}}$$

Where E_sum_ is the total energy of the whole, E_1_ is the energy of Br^-^, and E_2_ is the energy of the COF skeleton. Model structures for the calculations were constructed based on the characteristic repeating units of various covalent organic frameworks (COFs), ensuring both representativeness and periodicity of the systems under study.

Molecular Dynamics Simulations: All-atom molecular dynamics simulations were performed with Materials Studio 2023 (BIOVIA) using the Forcite module to examine the influence of an external electric field on ion transport in COF-IBPY and COF-DIBPY single crystals. Initial 3×3×3 super-cells of both crystals were built in Visualizer and relaxed through five consecutive geometry-optimization cycles (Forcite, Compass III force field, Smart algorithm) to eliminate close contacts and unreasonable local geometries. The minimized super-cells were then used as starting configurations for 500-ps NVT production runs (T = 298 K, Nosé thermostat, 1 fs time step, frames recorded every 500 steps) in which the lattice atoms were kept fixed while a static electric field of 0.3 V Å^-1^ was applied along the -Z direction (parallel to the c vector) to accelerate ionic motion. The external electric field strength was chosen in line with values employed in previously reported computational studies. Trajectories were saved every 0.5 ps and subsequently processed with Forcite Analysis to extract solvation-structure snapshots and the mean-square displacement (MSD) of Br⁻; diffusion coefficients were obtained from the linear regime of the MSD curves. Visualization and rendering of simulation snapshots were carried out with VMD software. In the Fickian (linear) regime the MSD grows linearly with time; the self-diffusion coefficient D is extracted from the Einstein relation:

$$\text{D=}\frac{\text{1}}{\text{6}}\lim_{\text{t→∞}} \frac{\text{d}}{\text{dt}}\text{MSD(t)}$$

The aforementioned formula is applicable solely for calculating the mean square displacement (MSD) in three-dimensional space. ^[3]^ The electrophoretic mobility in the Z-direction (electric field direction) within the molecular dynamics’ simulation box is calculated using the following formula:

$$\text{MSD}_{\text{Z}}\text{ = }{\text{D}_{\text{z}}}^{\text{2}}\text{+ 2}\text{D}_{\text{z}}\text{D}_{\text{E}}\text{+}{\text{D}_{\text{E}}}^{\text{2}}$$

$$\text{D}_{\text{Z}}\text{ =} \text{V}_{\text{d}}\text{*E*t}$$

where, D_z_ is the free diffusion distance in the z-direction, D_E_ is the migration distance under electric field drive, V_d_ is the electrical mobility, E is the electric field intensity, and t is time. Since D_z_ is much smaller than D_E_, the terms D_Z_^2^ and D_E_D_Z_ can be neglected. Thus, the formula can be simplified to the following expression:

$$\text{MSD}_{\text{Z}}\text{=} {\text{(}\text{V}_{\text{d}}\text{*E*t)}}^{\text{2}}$$

Finally, V_d_ was calculated by the above formula.

**Supplementary Equations**

The optical bandgap of COF was analyzed using the tau-c method, and the calculation formula is as follows:

$\text{(αhν)}^{\text{2}}$=$\text{A(hν-}\text{E}_{\text{g}}\text{)}$

Where α is the absorption index, h is Planck's constant, $\nu$ is the frequency, E_g_ is the semiconductor bandgap width, and A is a constant.

The UPS can provide an energy estimate highest valence band energy level and Fermi level (E_F_) relative to the vacuum energy level can be determined. The calculation formula is as follows:

$$\text{E}_{\text{VBM}}\text{=-hv+}\text{E}_{\text{Cutoff}}\text{-}\text{E}_{\text{onset}}$$

$$\text{E}_{\text{F}}\text{=-ϕ=-hv+}\text{E}_{\text{cutoff}}$$

Where $\text{E}_{\text{onset}}$ denotes the valence band onset and $\text{E}_{\text{Cutoff}}$ represents the cutoff energy. *hν, E_F_, and* $\phi$ represent the energy of the incident photon (*He-I, 21.22 eV*), the Fermi level, and the work function, respectively. Combined with the experimentally measured optical bandgap, the energy value of the conduction band energy level (E_CBM_) relative to the vacuum level can then be derived.

Modify the normalized conductivity value of the memristor using the following formula: ^[4]^

$$\text{G}^{\text{-}}\text{=}\text{G}_{\text{max}}^{\text{-}}\text{+}\frac{\text{G}_{\text{max}}^{\text{-}} \text{-}\text{G}_{\text{min}}^{\text{+}}}{\text{1-}\text{e}^{\text{-ν}^{\text{-}}\text{∙}\text{P}_{\text{max}}}}\left( \text{1-}\text{e}^{\text{ν}^{\text{-}}\text{⋅}\text{(P-}\text{P}_{\text{max}}} \right)$$

$$\text{G}^{\text{+}}\text{=}\text{G}_{\text{min}}^{\text{+}}\text{+}\frac{\text{G}_{\text{max}}^{\text{-}} \text{-}\text{G}_{\text{min}}^{\text{+}}}{\text{1-}\text{e}^{\text{-ν}^{\text{+}}\text{∙}\text{P}_{\text{max}}}}\left( \text{1-}\text{e}^{\text{-}\text{ν}^{\text{+}}\text{⋅}\text{P}} \right)$$

where G^+^ and G^-^ denote the normalized conductance for LTP and LTD respectively. ν^+^ and ν^-^ represent the nonlinear fitting coefficients for LTP and LTD respectively. P denotes the number of pulses.

**Synthesis methods**

**Synthesis of** **4,4',4'',4'''-(pyrene-1,3,6,8-tetrayl) tetra-benzaldehyde (PY-CHO).**

First, 1,4-dioxane (80 ml) and DI water (20 ml) were mixed and aerated under argon for 30 min. Then 1,3,6,8-tetrabromopyrene (3.0 g, 5.79 mmol), 4-formylphenylboronic acid (3.91 g, 26.06 mmol), K_2_CO_3_ (4.5 g, 32.835 mmol) and Pd (PPh_3_)_4_ (669.1 mg, 0.58 mmol) were dissolved in the above solution. The mixture was stirred at 90 ℃ for 24 h. After cooling to room temperature, the reaction mixture was filtered and washed several times with DI water, acetone and methanol, and finally dried in a vacuum oven at 50 ℃ for 24 h to obtain yellow solid (Yield: 85.6%). ^1^H NMR (400 MHz, CDCl_3_): δ = 10.13 (s, 4 H), 8.16 (s, 4 H), 8.10 (d, 8 H), 8.04 (s, 2 H), 7.86 (d, 8 H).

**Synthesis of** **5,5'-bis(bromomethyl)-2,2'-bipyridyl.**

5,5'-dimethyl-2,2'-dipyridyl (3 g, 10.8 mmol), N-bromosuccinimide (3.9 g, 21.8 mmol) and azobisisobutyronitrile (40 mg, 0.24 mmol) were added to a round-bottomed flask under an argon atmosphere, followed by 120 ml of CCl_4_. The reaction mixture was stirred overnight at 85 °C and filtered hot. After concentration of the filtrate under vacuum, the crude solid was purified in methanol by sonication to give white powder (yield: 55%). 1H NMR (CDCl3, 400 MHz): δ 8.61 (d, J = 2.1 Hz, 2H), 8.33 (d, J = 8.2 Hz, 2H), 7.79 (dd, J = 8.2, 2.3 Hz, 2H), 4.47 (s, 4H).

**Synthesis of** **2,2'-([2,2'-bipyridine]-5,5'-diyl) di-acetonitrile (Py-CN).**

5,5'-bis(bromomethyl)-2,2'-bipyridyl (1.5 g, 4.39 mmol) and sodium cyanide (1.35 g, 27.55 mmol) were added to a round-bottomed flask under an argon atmosphere, followed by dimethyl sulfoxide (80 mL). The reaction mixture is stirred at 45°C for 6 h and then at room temperature for 12 h. The solution was heated to 80 ℃and poured without cooling into DI water (100 mL) filtered, and washed with DI water several times. After the crude product was dried in a 50 ºC oven, it was dissolved in methylene chloride and filtered using celite. The filtrate was concentrated and purified by silica gel column chromatography using methylene dichloromethane as eluent to obtain white solid (yield:56.1 %). ^1^H NMR (400 MHz, CDCl_3_): δ 8.67 (s, 2H), 8.49 (d, J = 8.2 Hz, 2H), 7.88 (dd, J = 8.2, 2.3 Hz, 2H), 3.87 (s, 4H).

**Synthesis of COF-PY Powder and Film.**

Dissolve Py-CHO (12.4 mg, 0.02 mmol), By-CN (9.36 mg, 0.04 mmol) and Cs₂CO₃ (81.26 mg, 0.25 mmol) in o-DCB (0.5 ml) and n-BuOH (0.5 ml), then transfer the mixture to a Pyrex tube. After sonication for 10 minutes, add the mixture to a patterned glass substrate, which was then rapidly cooled to 77 K in a liquid nitrogen bath and sealed under vacuum. The reaction mixture was then heated at 120 °C for three days. The resulting precipitate was successively washed with H₂O, THF, DCM and DMF, and then dried under vacuum at 50 °C overnight to yield an orange powder and film. Yield: 86.5%.

**Synthesis of COF-BPY Powder and Film.**

Dissolve Py-CHO (12.4 mg, 0.02 mmol), BPy-CN (9.4 mg, 0.04 mmol) and Cs₂CO₃ (81.26 mg, 0.25 mmol) in o-DCB (0.5 ml) and n-BuOH (0.5 ml), then transfer the mixture to a Pyrex tube. After sonication for 10 minutes, add the mixture to a patterned glass substrate, which was then rapidly cooled to 77 K in a liquid nitrogen bath and sealed under vacuum. The reaction mixture was then heated at 120 °C for three days. The resulting precipitate was successively washed with H₂O, THF, DCM and DMF, and then dried under vacuum at 50 °C overnight to yield an orange powder and film. Yield: 81.1%.

**Synthesis of COF-IBPY Powder and Film.**

COF-BPY (49 mg) was dispersed in a mixture of DMF (2 mL) and bromoethane (18 mg, 0.096 mmol). A glass substrate coated with thin film was then added. The reaction mixture was heated at 135°C for 24 hours. The resulting precipitate was sequentially washed with H₂O, THF, DCM, and DMF, and dried under vacuum at 50°C overnight, yielding brown powder and film.

**Synthesis of COF-DIBPY Powder and Film.**

COF-BPY (49 mg) was dispersed in a mixture of DMF (2 mL), dibromoethane (18 mg, 0.096 mmol), and acetic acid (0.5 mL). A glass substrate coated with a thin film was then added. The reaction mixture was heated at 135°C for 24 hours. The resulting precipitate was sequentially washed with H₂O, THF, DCM, and DMF, and dried under vacuum at 50°C overnight, yielding brown powder and film.

**Device Fabrication**

Initially patterned Au glass substrates (1 cm × 0.5 cm) were successfully deposited with COF film on the substrate. Then, an Al top electrode (100 nm) was deposited on the active layer using vacuum thermal evaporation with a shadow mask, maintaining a chamber vacuum of 10-7 Torr. The electrode spacing and electrode width were both 0.2 mm.


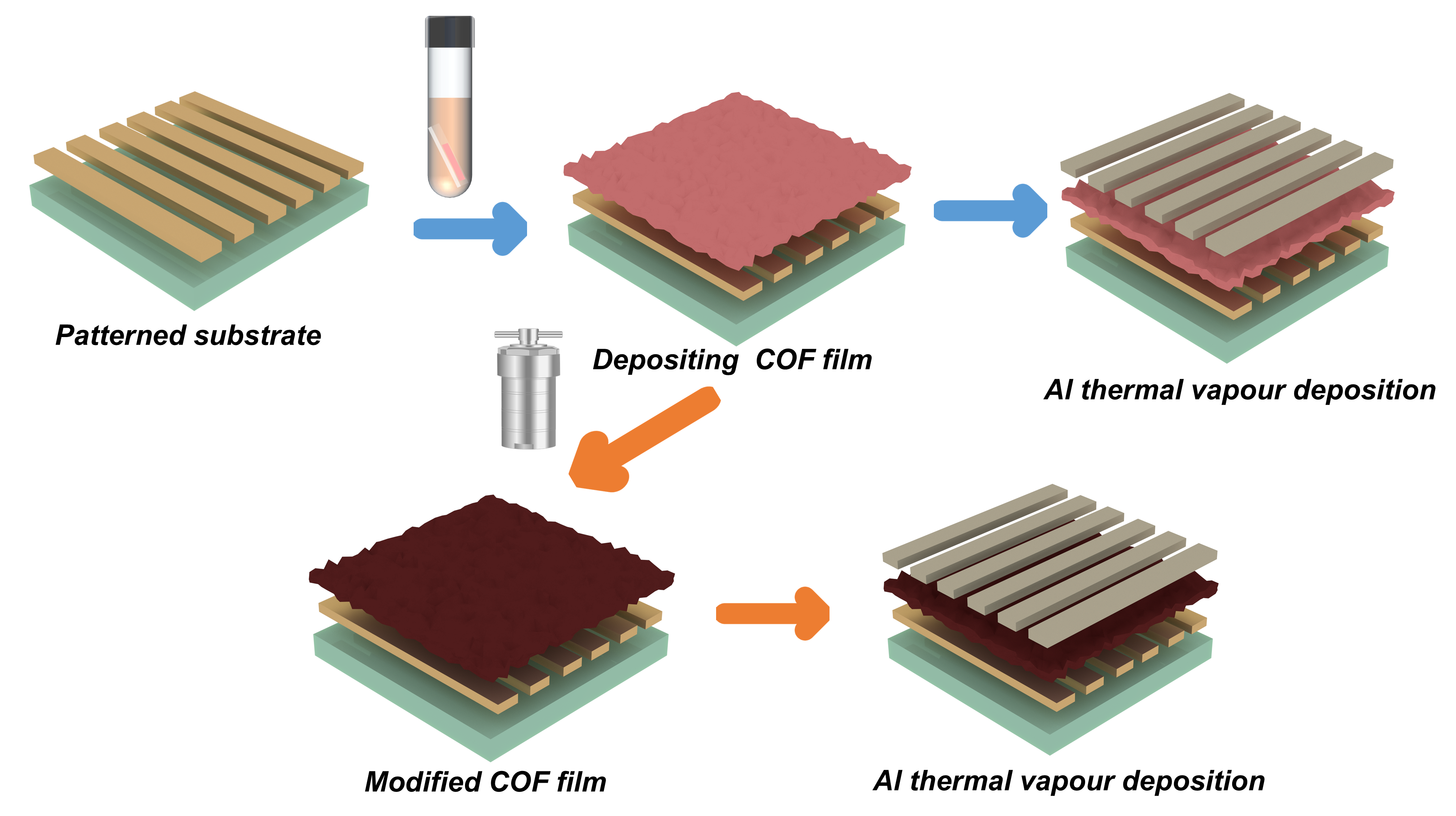


**Scheme S1**. Device fabrication process diagram.

**Characterization**


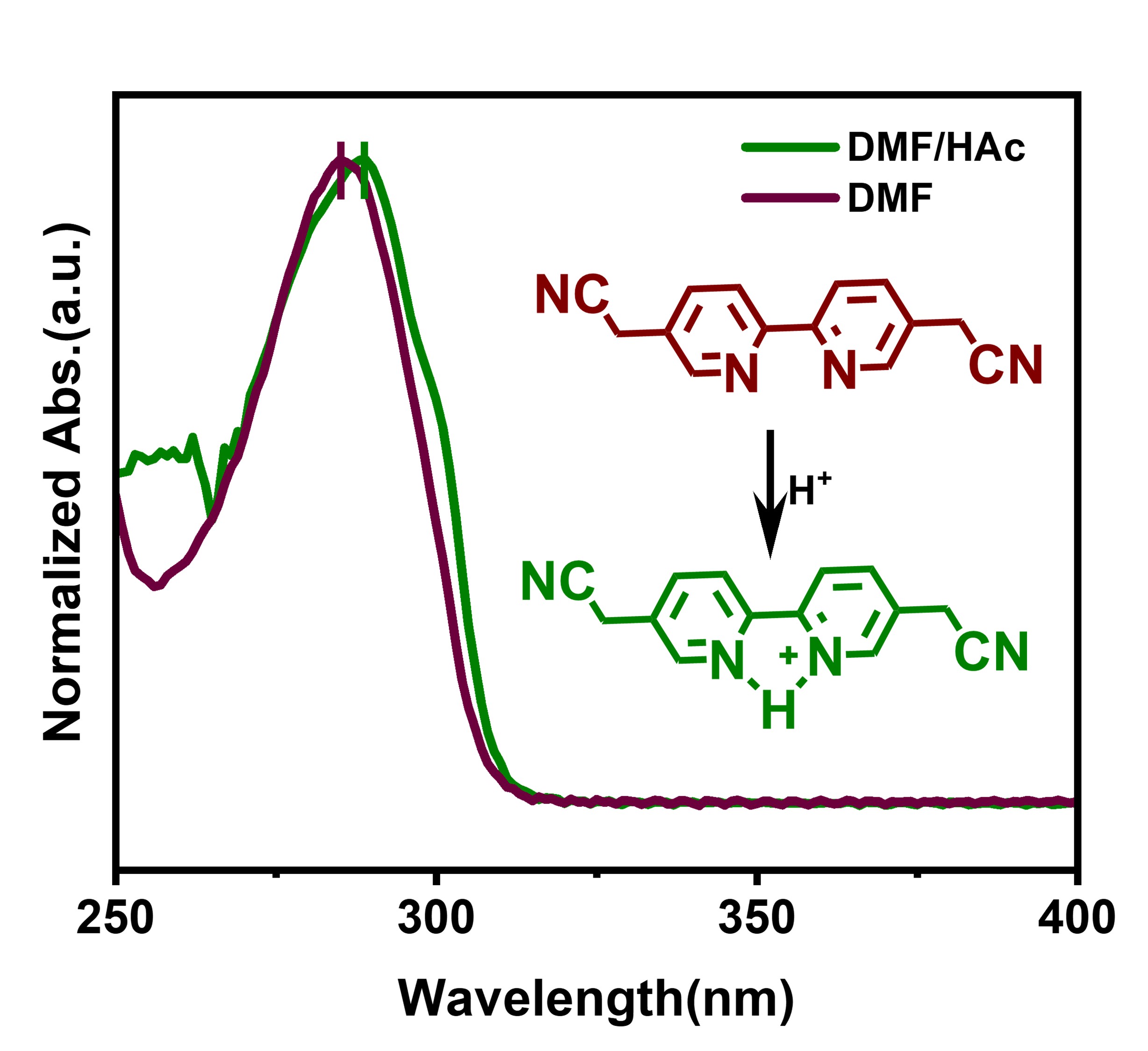


**Figure S1**. UV-visible spectra of BPy-CN before and after the addition of 6 M HAc.


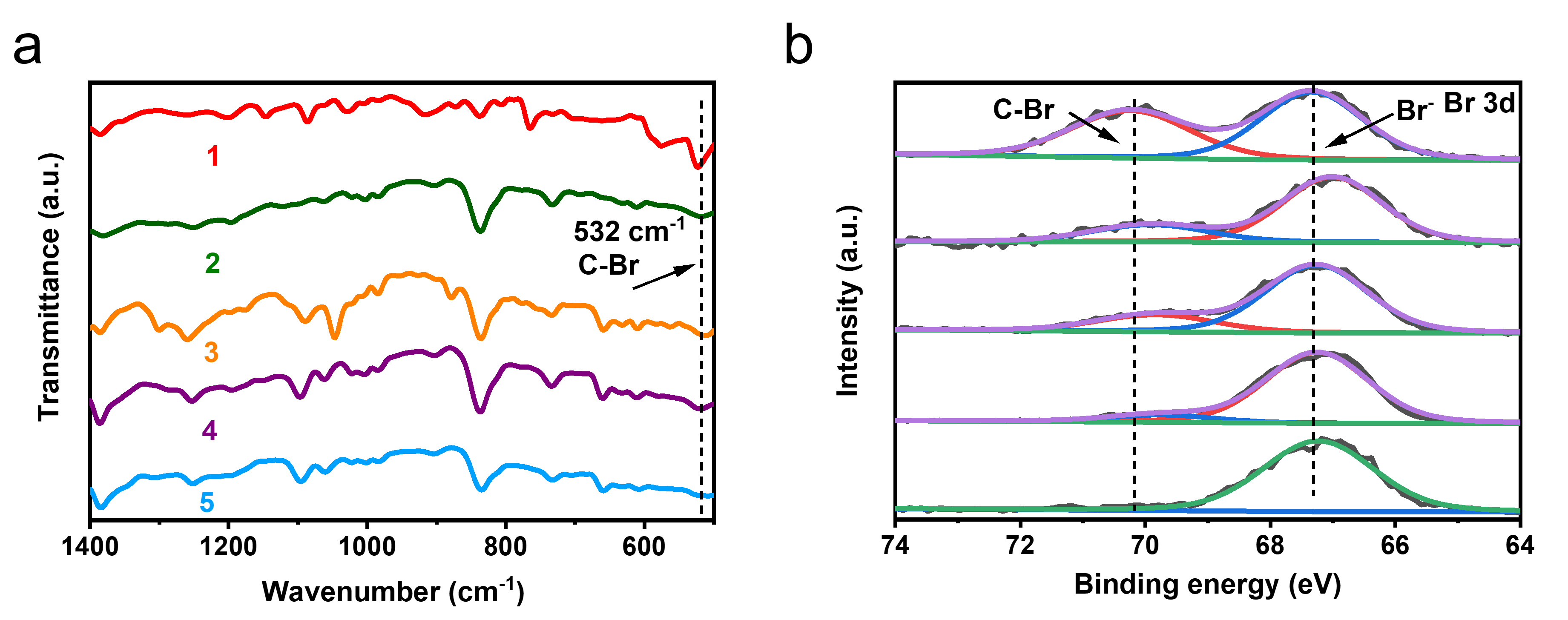


**Figure S2**. (a) FTIR and (b) XPS of COF-DIBPY after reactions under different conditions. See **Table S1** for detailed reaction conditions.

**Note**: In the infrared spectrum, the characteristic peak at 532 cm^-1^ is attributed to the characteristic vibration peak of C-Br. Its weakening indicates that the quaternized material does not contain a pendant alkyl chain structure. In the XPS spectrum, the binding energies at 67.2 eV and 70.3 eV correspond to the 3d orbital peaks of Br^-^ and C-Br, respectively. ^[5]^ The disappearance of the characteristic peak at 70.3 eV indicates that the quaternization ring formation has successfully taken the dominant position.


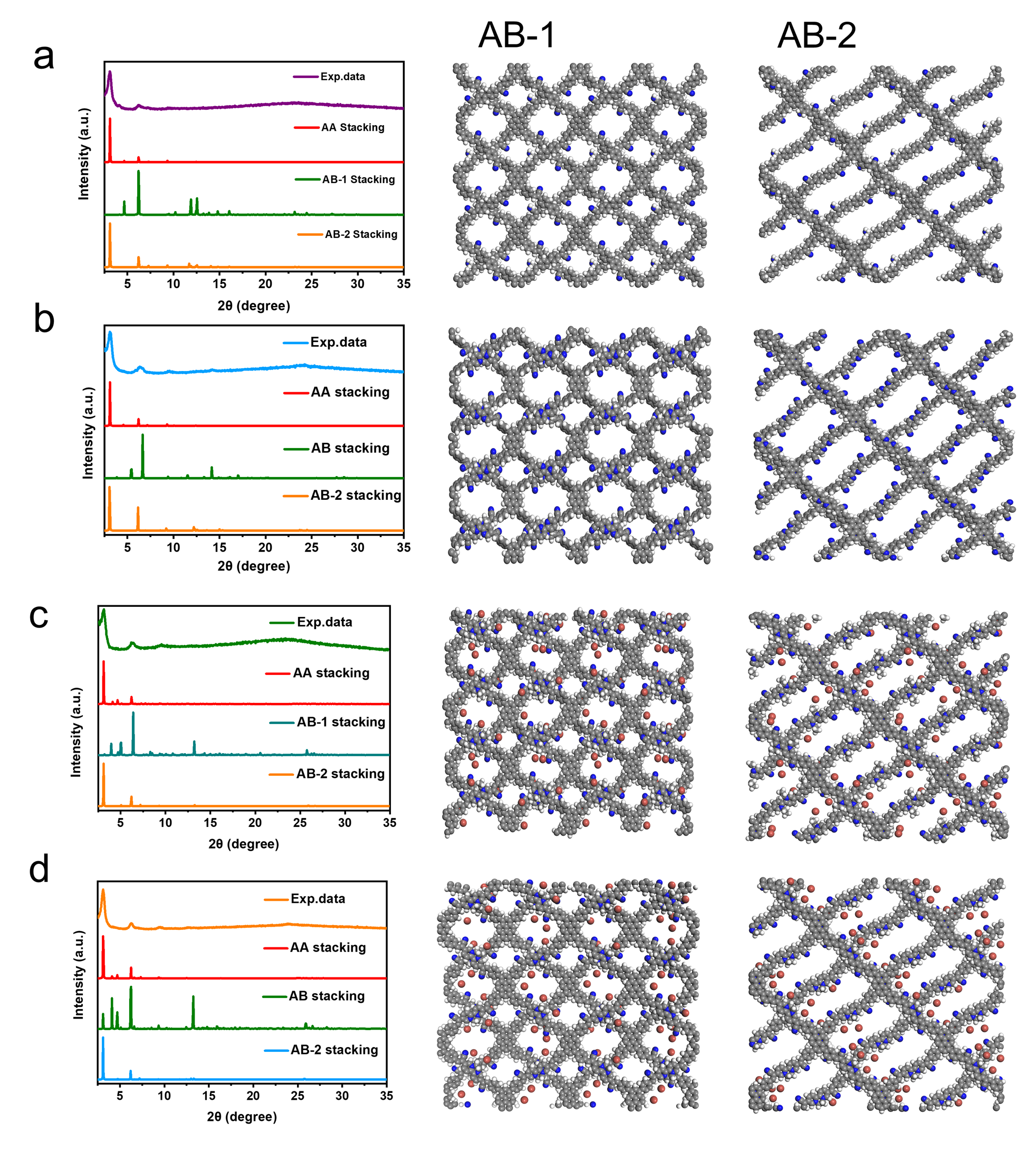


**Figure S3**. (a) XRD diffraction patterns of COF-PY, (b) COF-BPY, (c) COF-IBPY, and (d) COF-DIBPY, along with corresponding AB-1 and AB-2 stacking model diagrams.


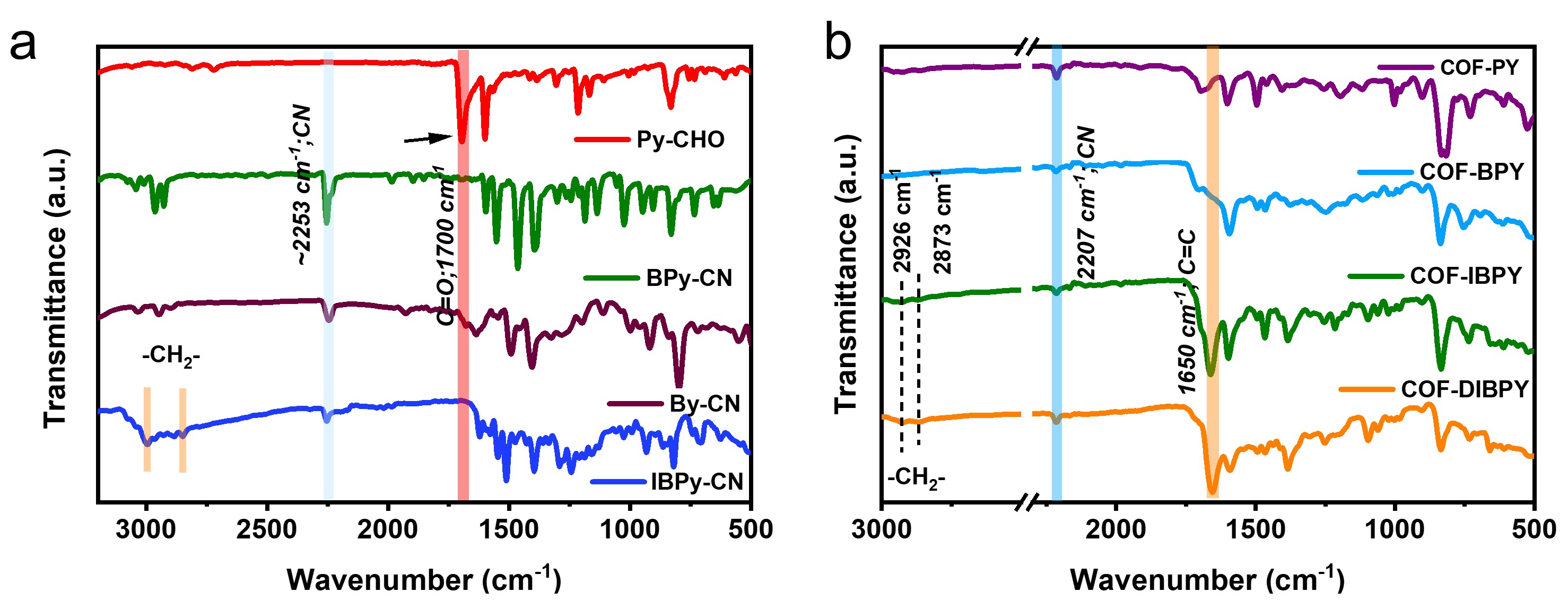


**Figure S4**. (a) Infrared spectra of PY-CHO, BY-CN, PY-CN, BPY-CN, and IBPY-CN, (b) Infrared spectra of COF-PY, COF-BPY, COF-IBPY, and COF-DIBPY.


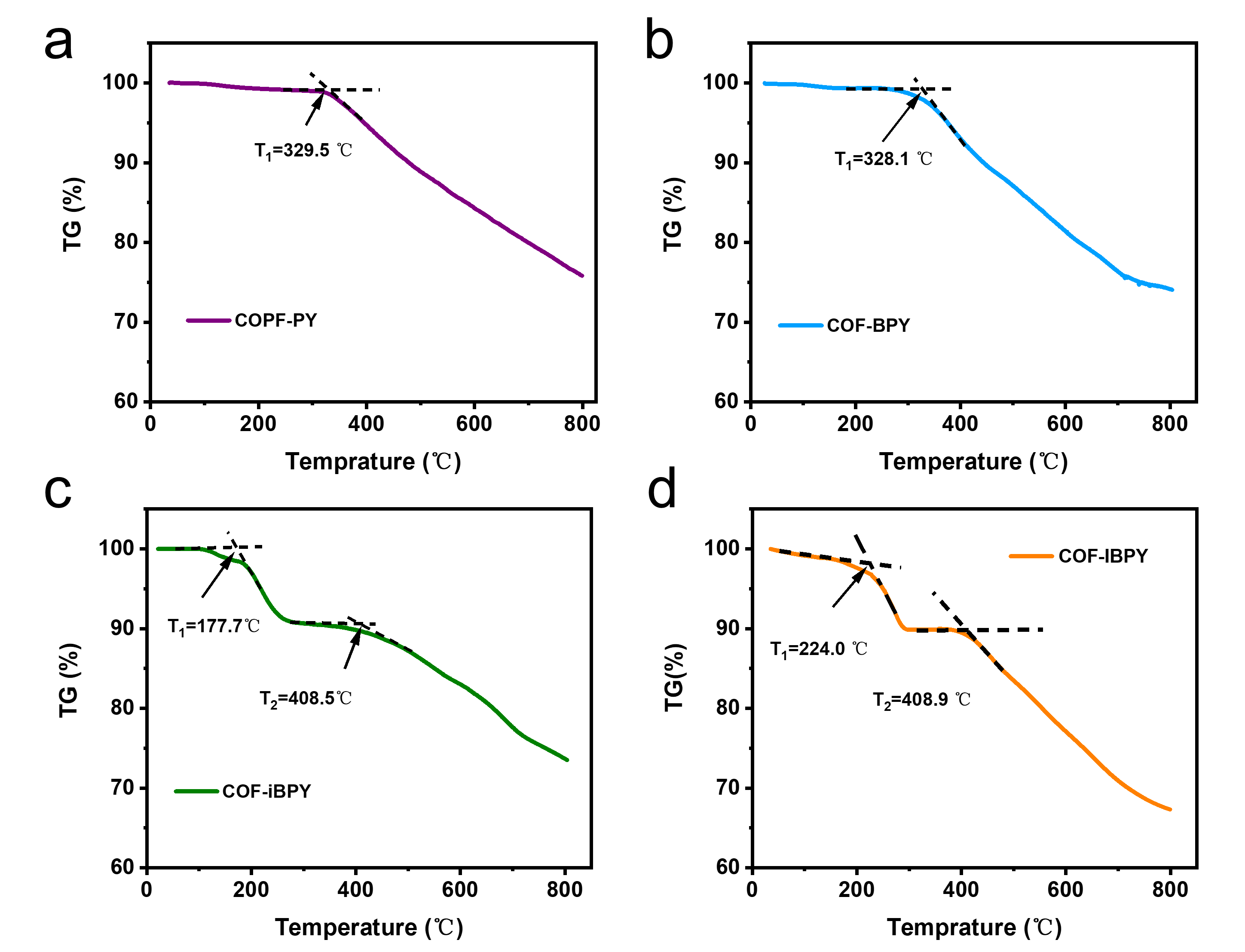


**Figure S5**. Thermal gravimetric analysis (TGA) of COF-PY, COF-BPY, COF-IBPY and COF-DIBPY.


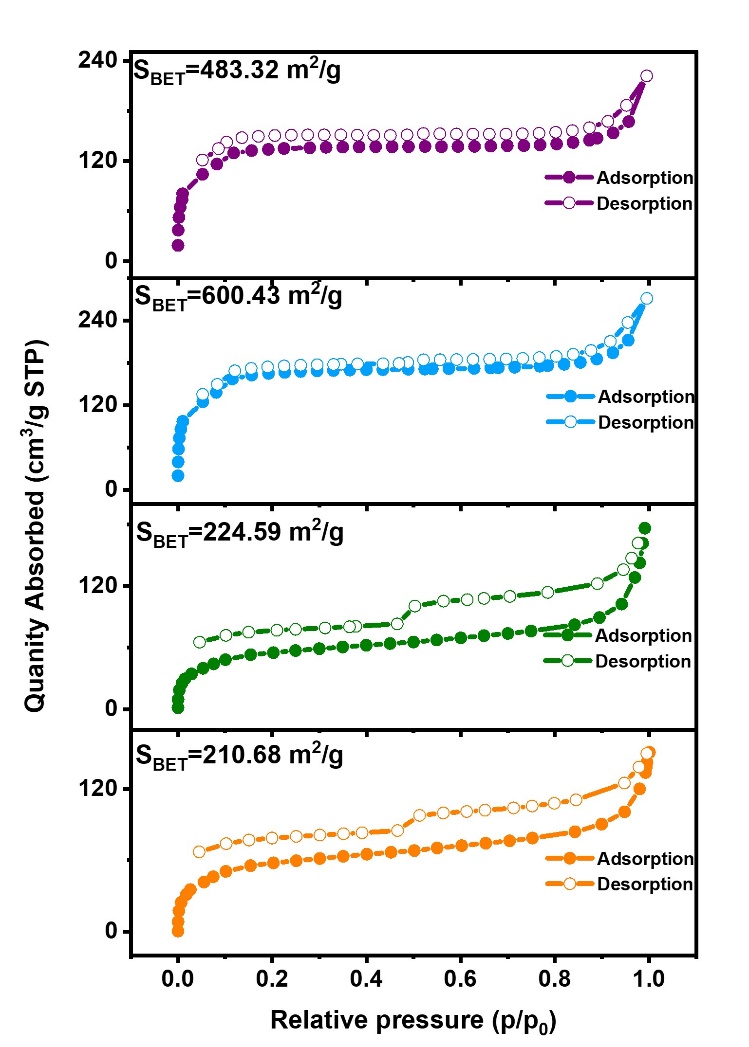


**Figure S6**. Nitrogen adsorption−desorption isotherms of four COFs.


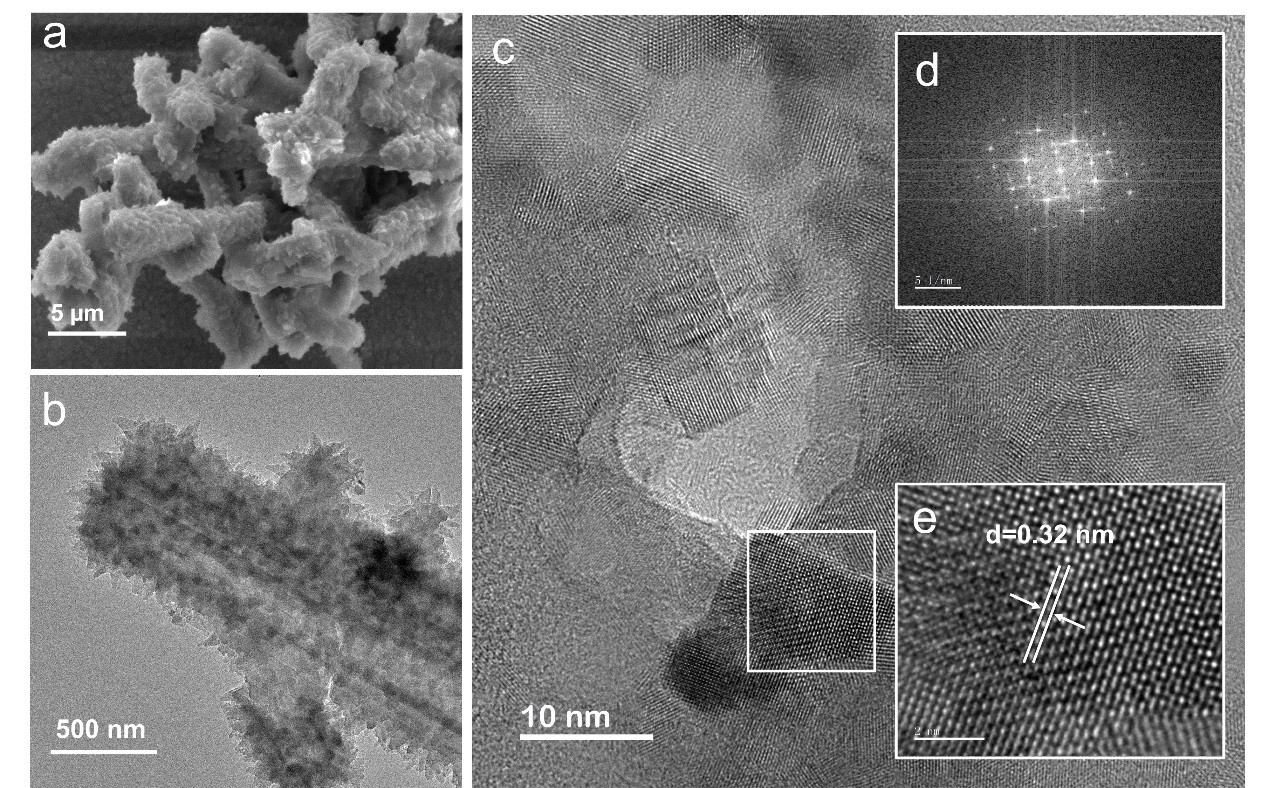


**Figure S7**. (a) SEM and (b) TEM images of COF-PY and (c) high-resolution lattice images. The inset shows the (d) Fourier transform pattern diagram and (e) Fourier filter image of the selected area.


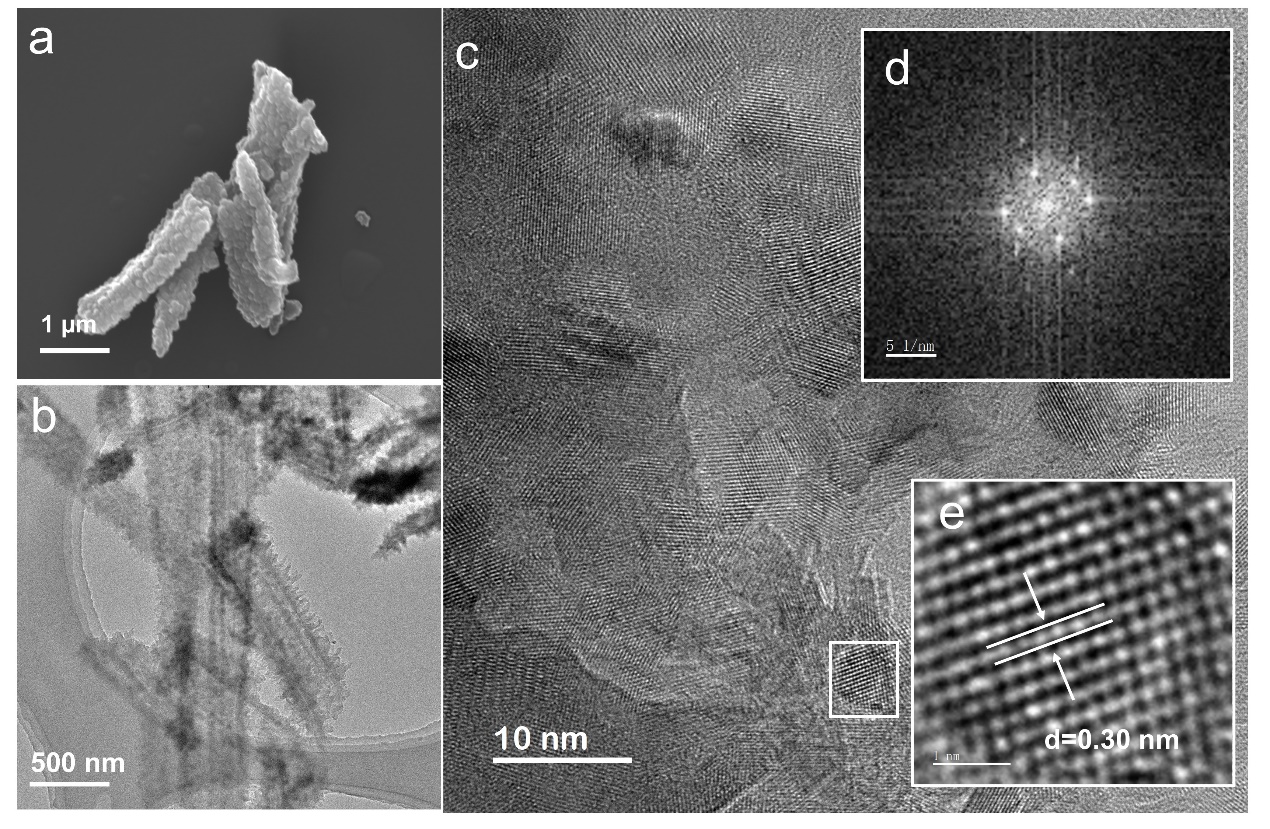


**Figure S8**. (a) SEM and (b) TEM images of COF-BPY and (c) high-resolution lattice images. The inset shows the (d) Fourier transform pattern diagram and (e) Fourier filter image of the selected area.


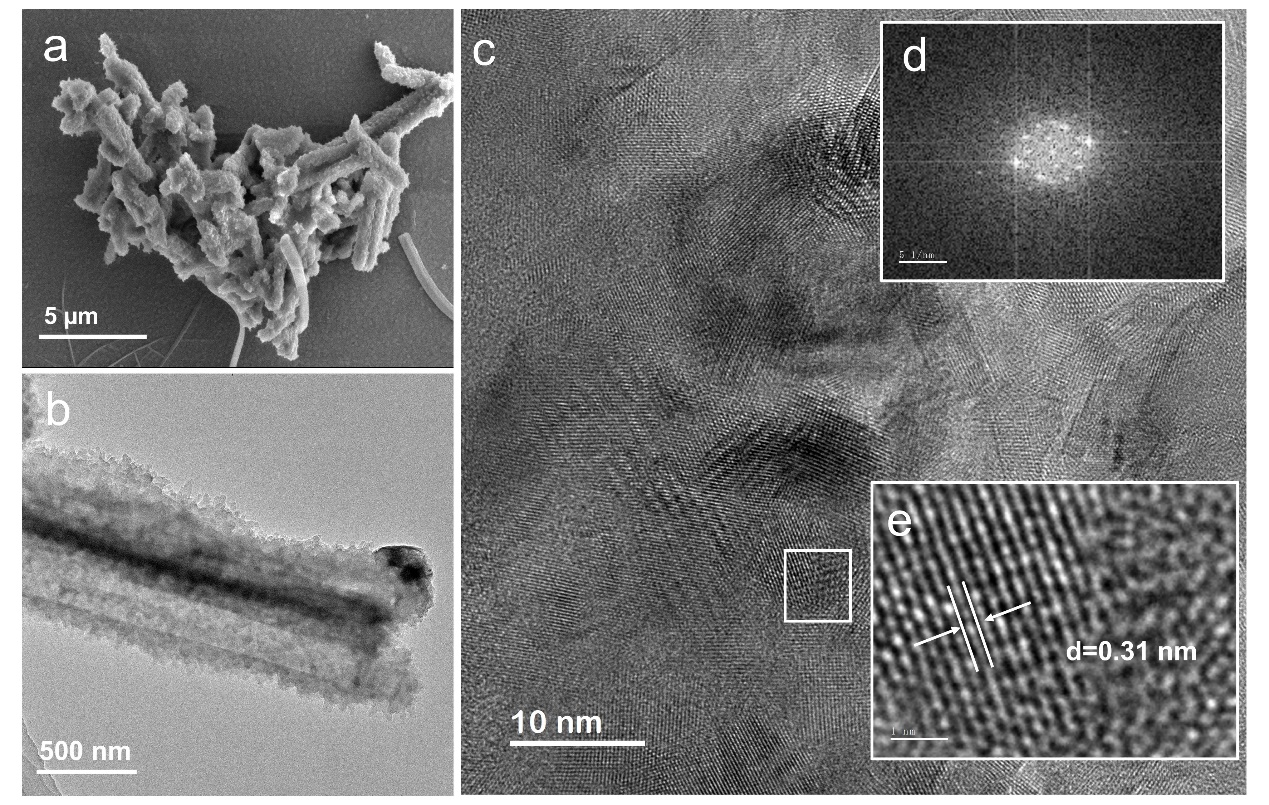


**Figure S9**. (a) SEM and (b) TEM images of COF-IBPY and (c) high-resolution lattice images. The inset shows the (d) Fourier transform pattern diagram and (e) Fourier filter image of the selected area.


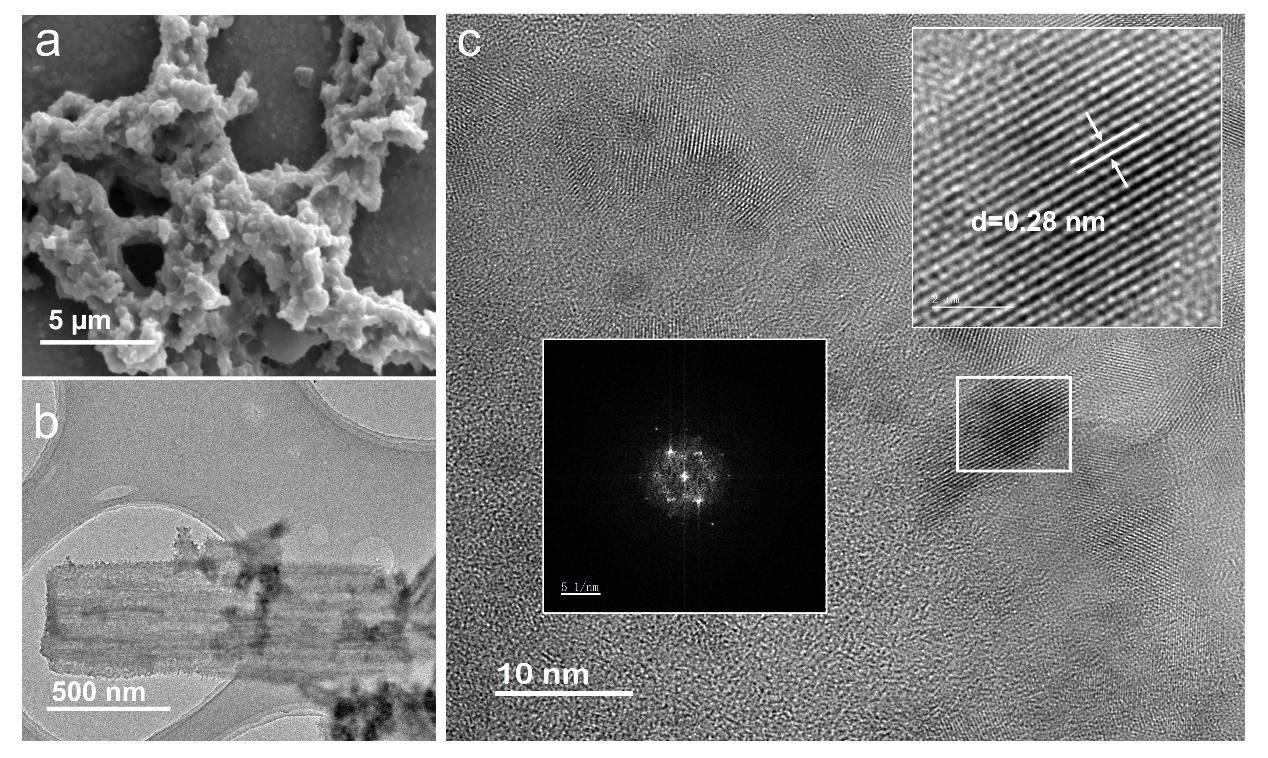


**Figure S10**. (a) SEM and (b) TEM images of COF-DIBPY and (c) high-resolution lattice images. The inset shows the (d) Fourier transform pattern diagram and (e) Fourier filter image of the selected area.


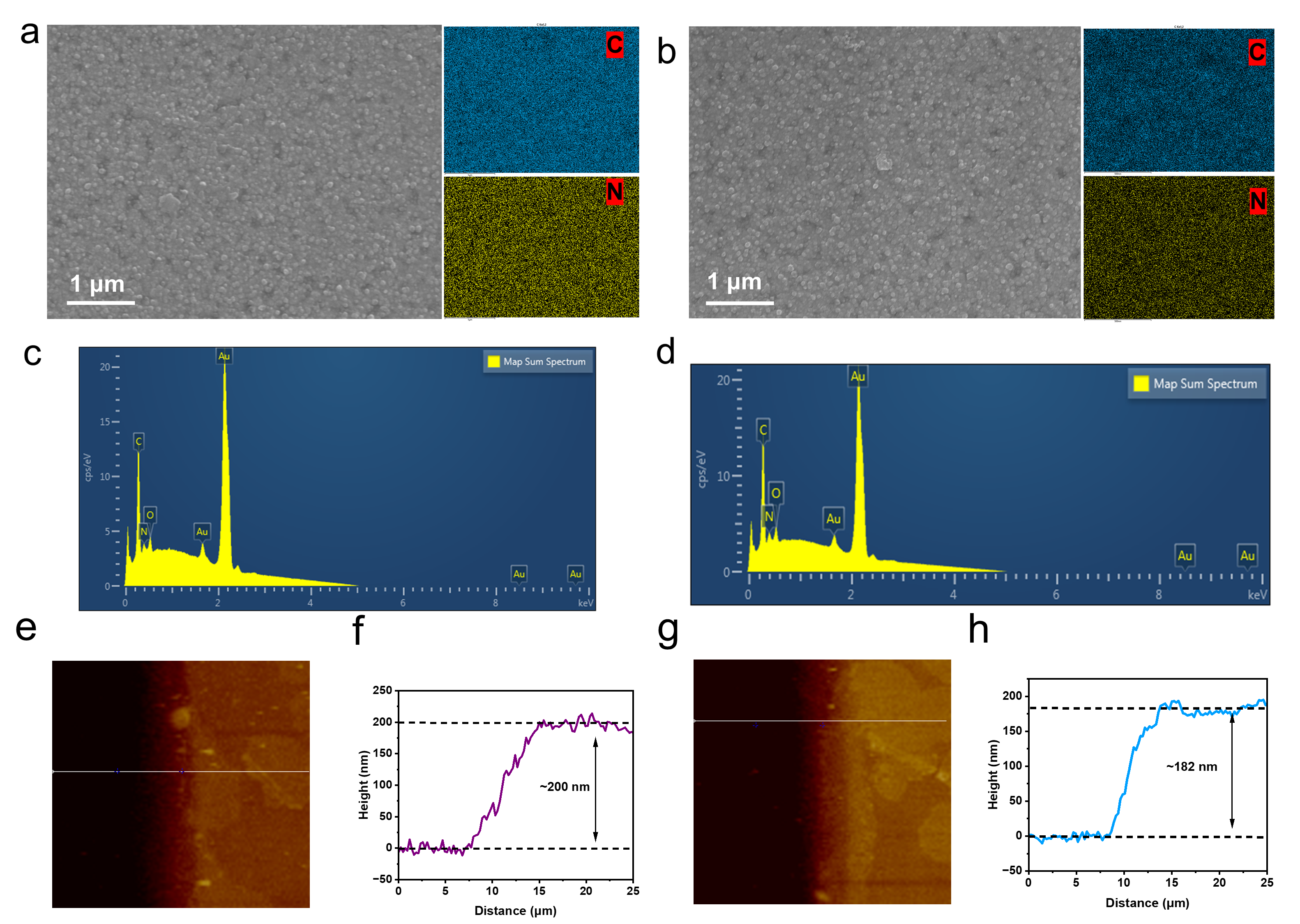


**Figure S11.** SEM images of the thin film surfaces of (a) COF-PY and (b) COF-BPY, as well as the corresponding EDS mapping images and (c and d) map sum spectra. AFM images of (e and f) COF-PY and (g and h) COF-BPY films and their corresponding height maps.


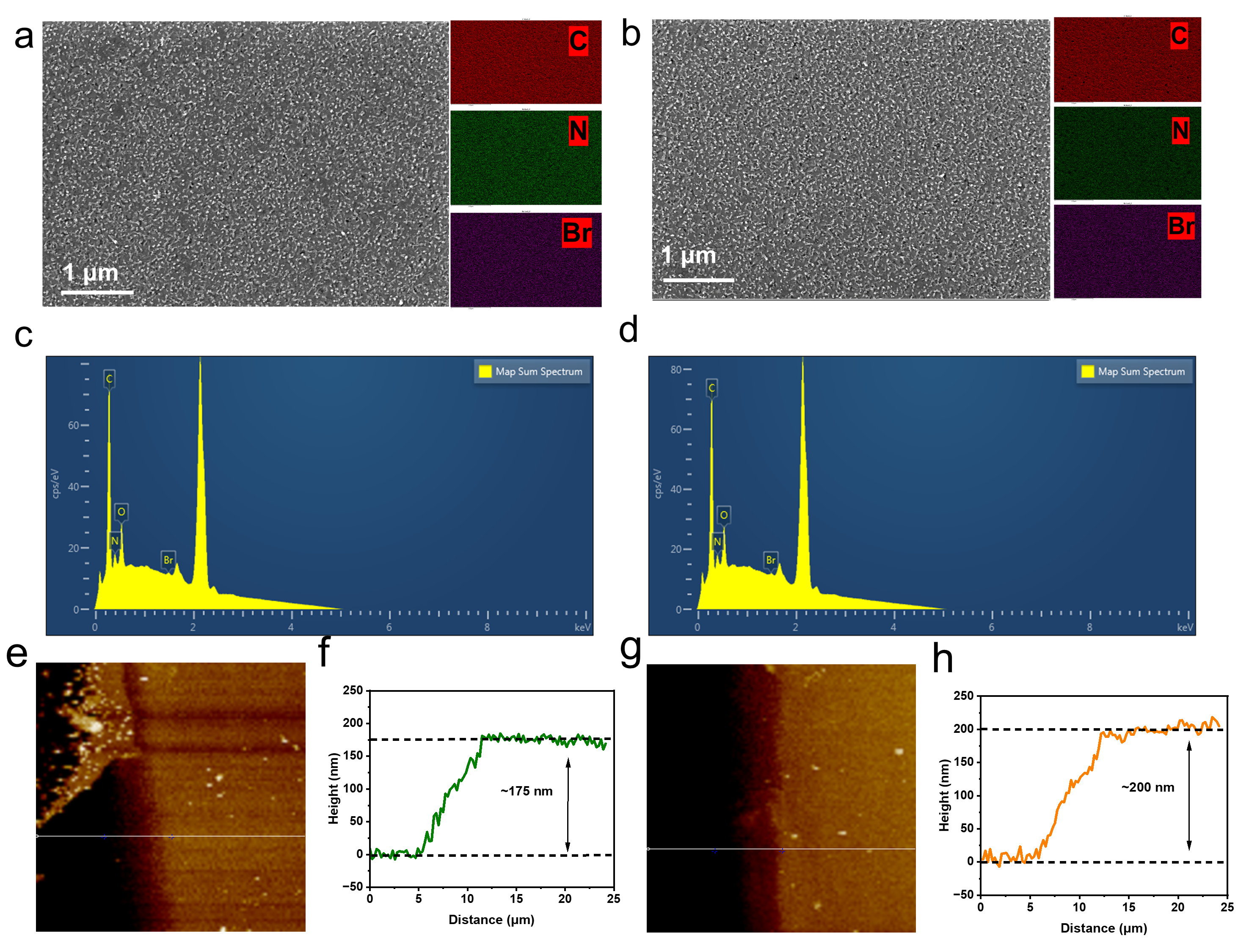


**Figure S12**. SEM images of the thin film surfaces of (a) COF-IBPY and (b) COF-DIBPY, as well as the corresponding EDS mapping images and (c and d) map sum spectra. AFM images of (e and f) COF-IBPY and (g and h) COF-DIBPY films and their corresponding height maps.


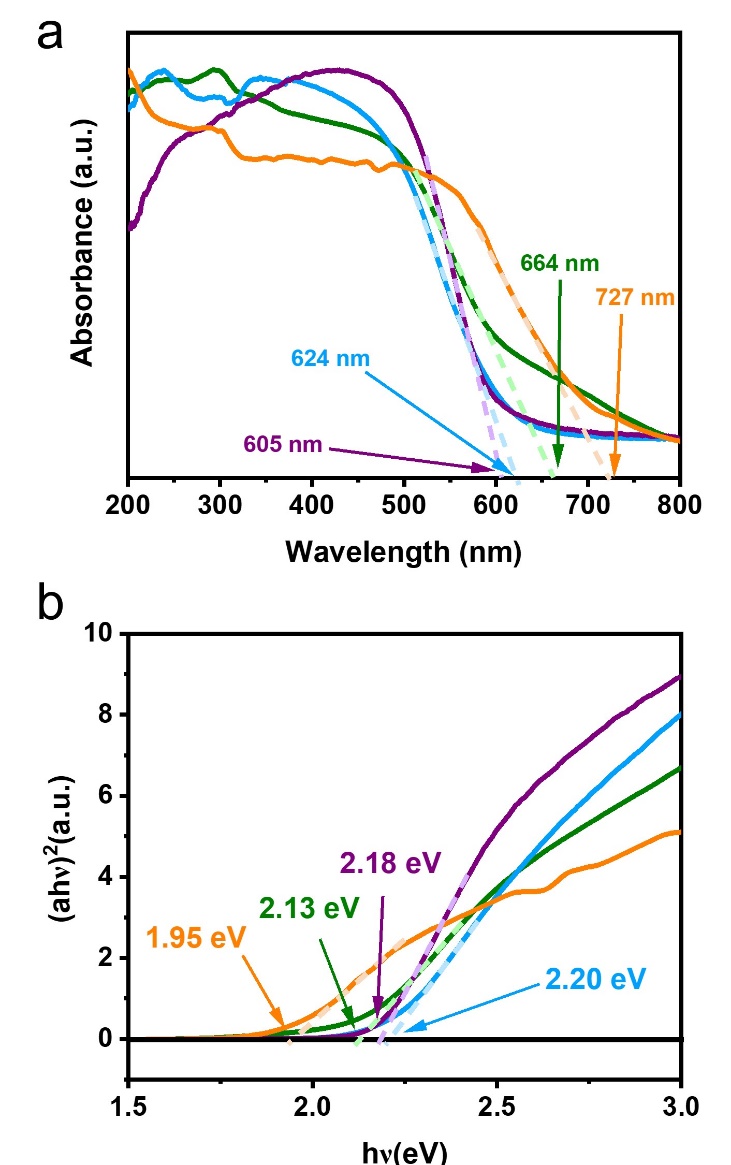


**Figure S13**. (a) UV/vis diffuse reflectance spectra. (b) Tau-c plot in the form of (αhυ)^2^ versus hυ. The purple curve represents COF-PY, the blue curve represents COF-BPY, the green curve represents COF-IBPY, and the orange curve represents COF-DIBPY.


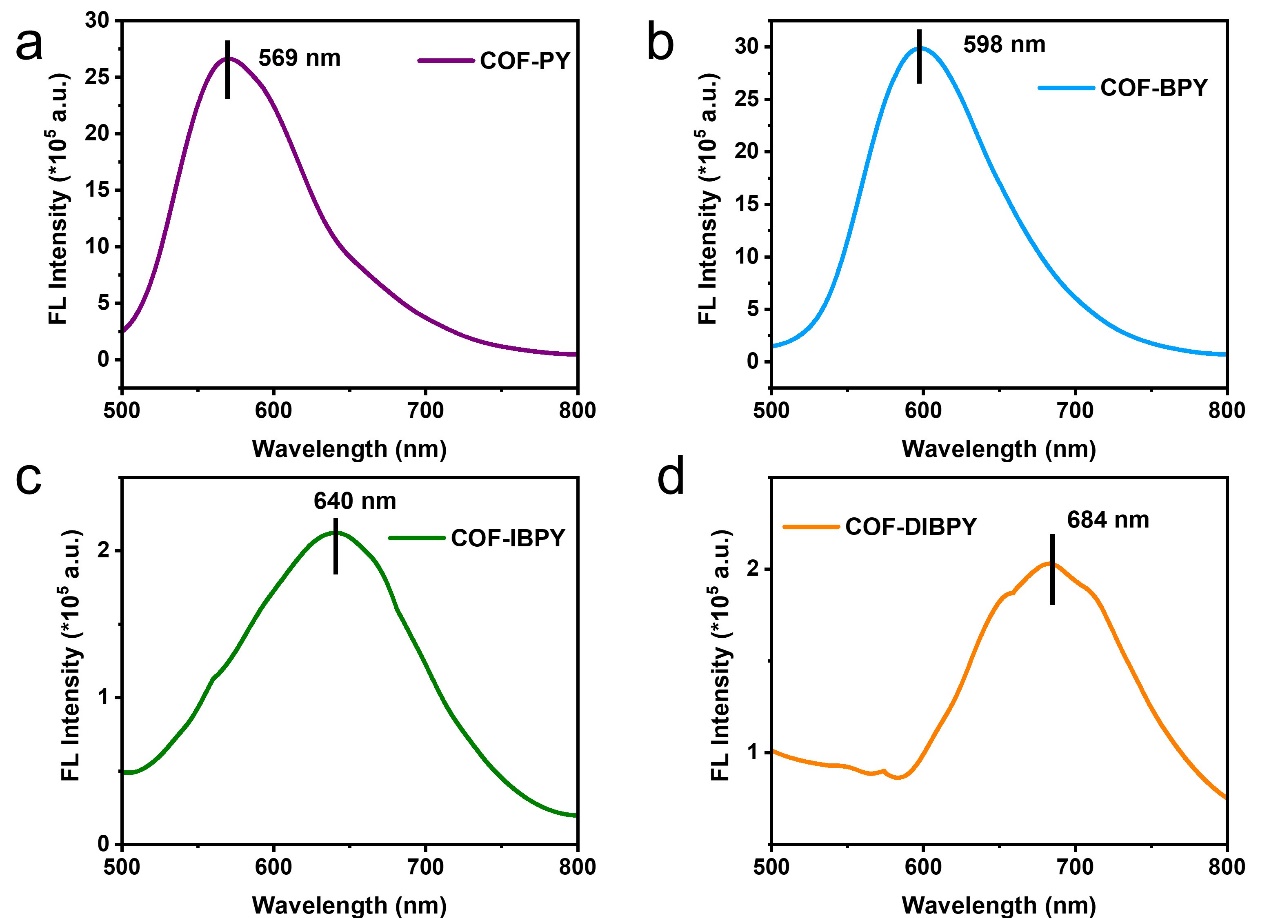


**Figure S14**. Fluorescence spectrum. Fluorescence emission spectra were measured in an ethanol dispersion solution at a concentration of 0.05 mg/ml with an emission wavelength of 350 nm.


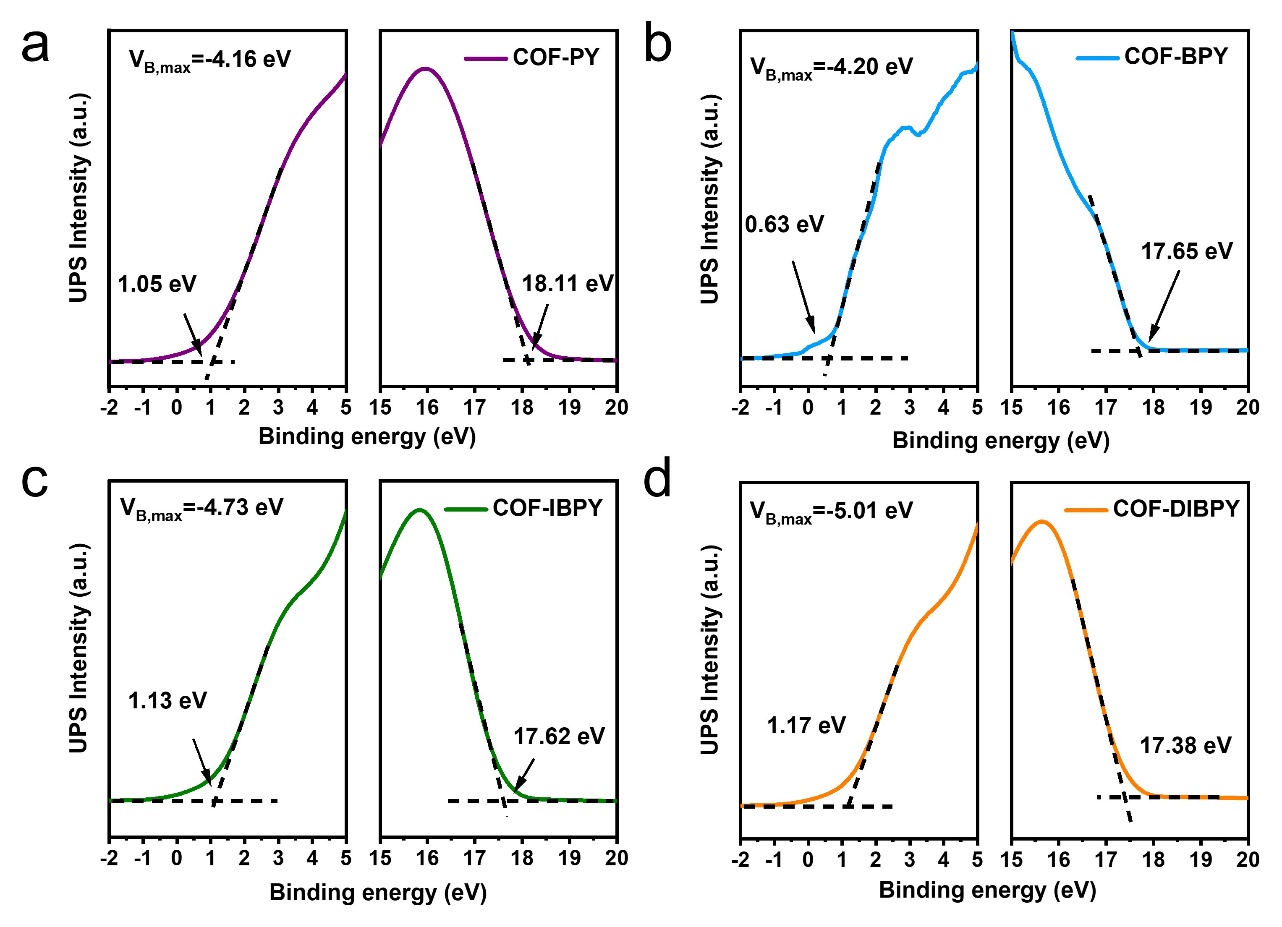


**Figure S15.** UPS spectra of the (a) COF-PY, (b) COF-BPY, (c) COF-IBPY and (d) COF-DIBPY.


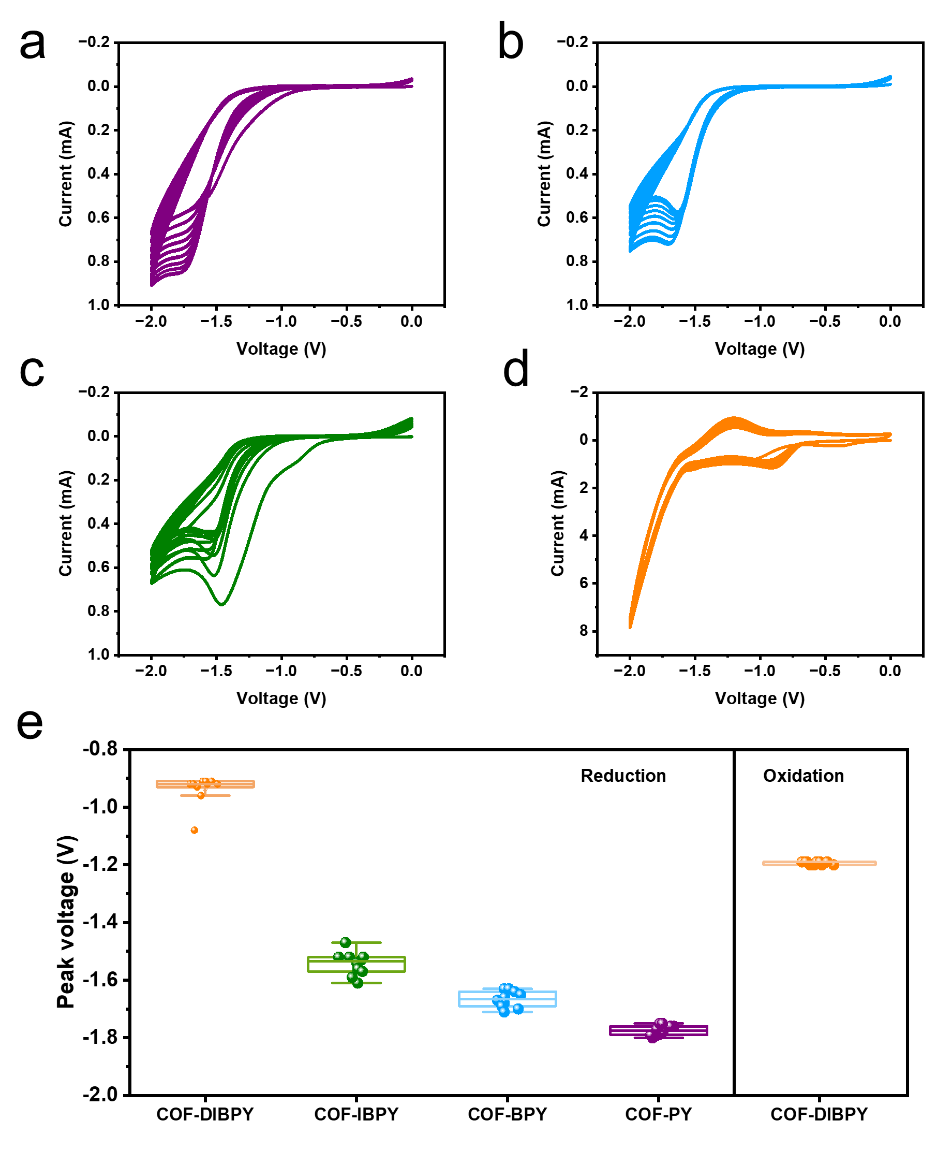


**Figure S16.** Cyclic voltammetry scan diagrams of (a) COF-PY, (b) COF-BPY, (c) COF-IBPY, and (d) COF-DIBPY, (e) with their peak potentials recorded from a, b, c, and d. The cyclic voltammogram (CV) of the four COFs at different scan rates in MeCN solution using tetrabutylammonium hexafluorophosphate (0.1 mol L^-1^) as supporting electrolyte.


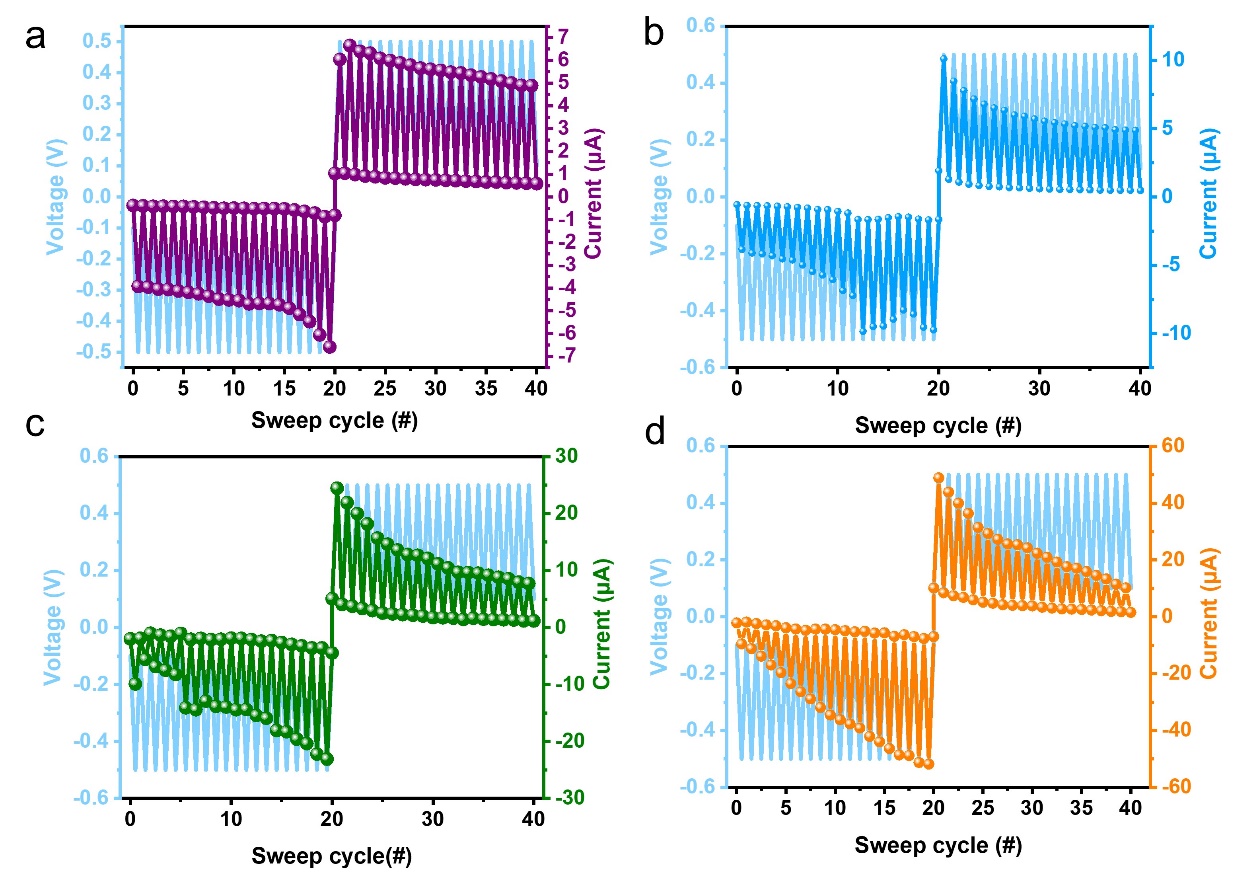


**Figure S17**. The tip current versus scan counts characteristic curves of memristors based on (a) COF-PY, (b) COF-BPY, (c) COF-IBPY, and (d) COF-DIBPY under continuous positive-negative voltage scanning (0 → 0.5 (-0.5) → 0), excerpted from Figures 3a-d.


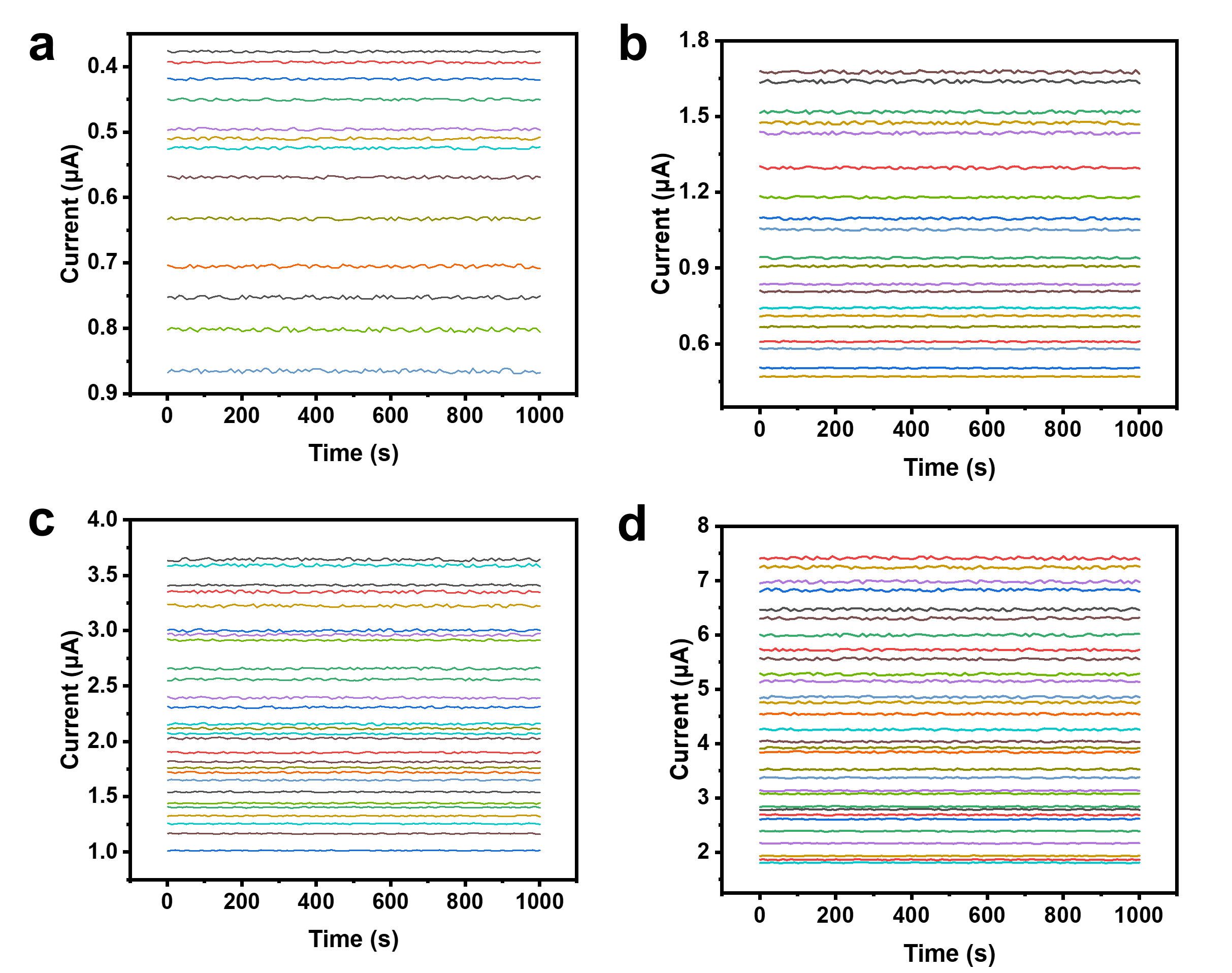


**Figure 18**. Stability of the devices (a) Al/COF-PY/Au,(b) Al/COF-BPY/Au, (c) Al/COF-IBPY/Au, and (d) Al/COF-DIBPY/Au after I–V scanning.(read at 0.1V)


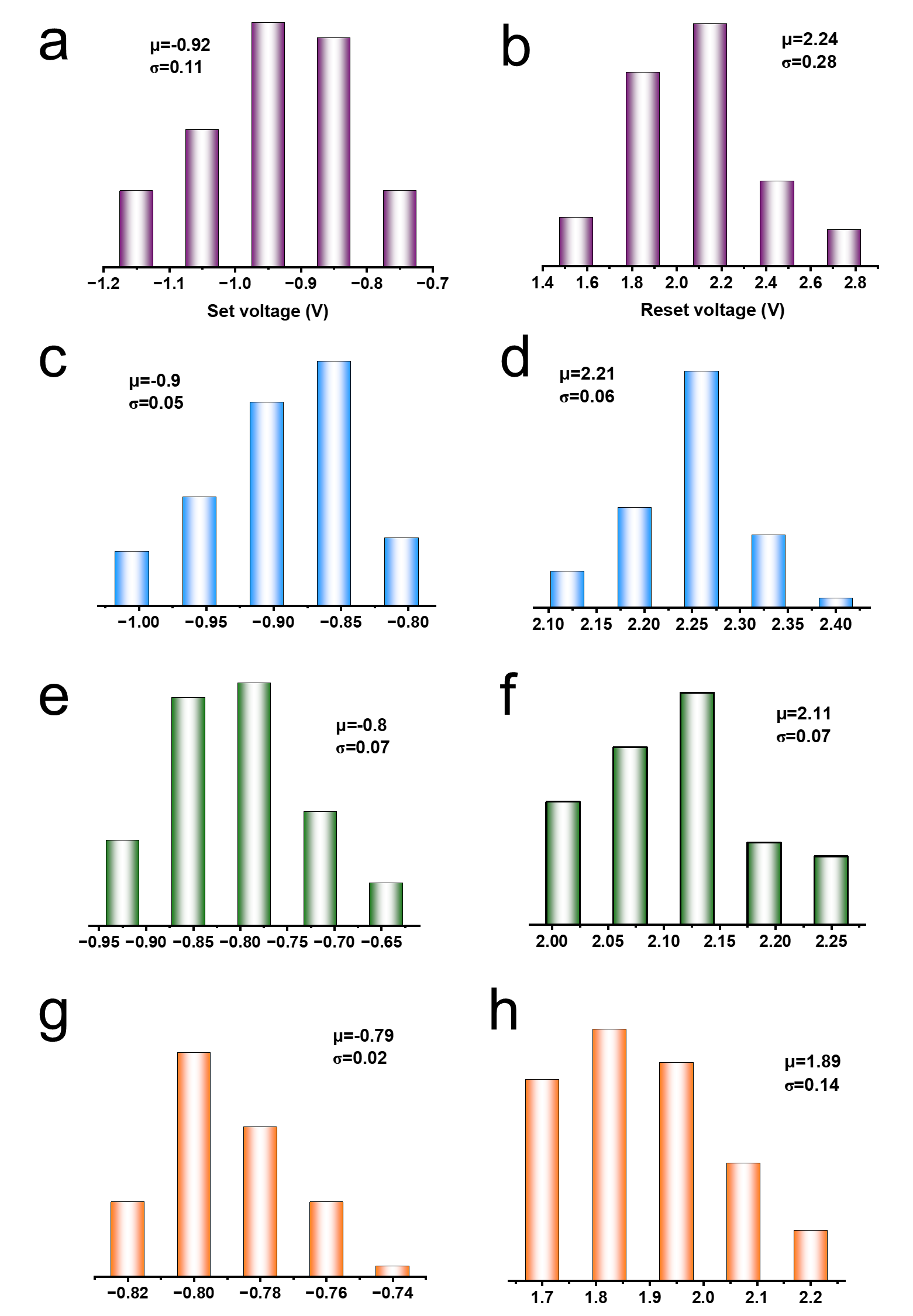


**Figure S19**. Statistical distribution of the V_set_ and V_reset_ based on (a) (b)Al/COF-PY/Au, (c) (d) Al/COF-BPY/Au, (e) (f) Al/COF-IBPY/Au, and (g) (h) Al/COF-DIBPY/Au.


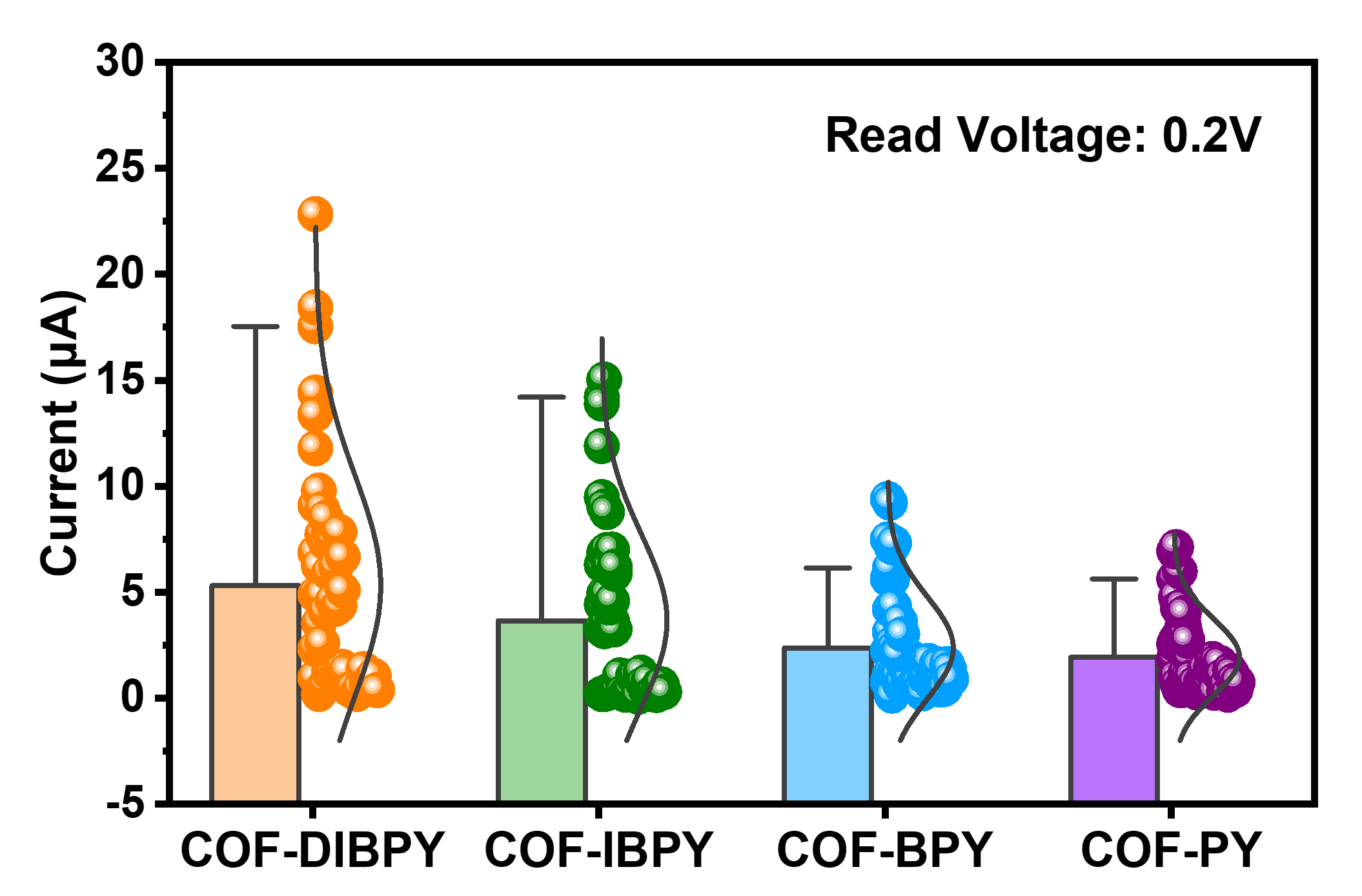


**Figure S20**. The readout current after each switch-off cycle.


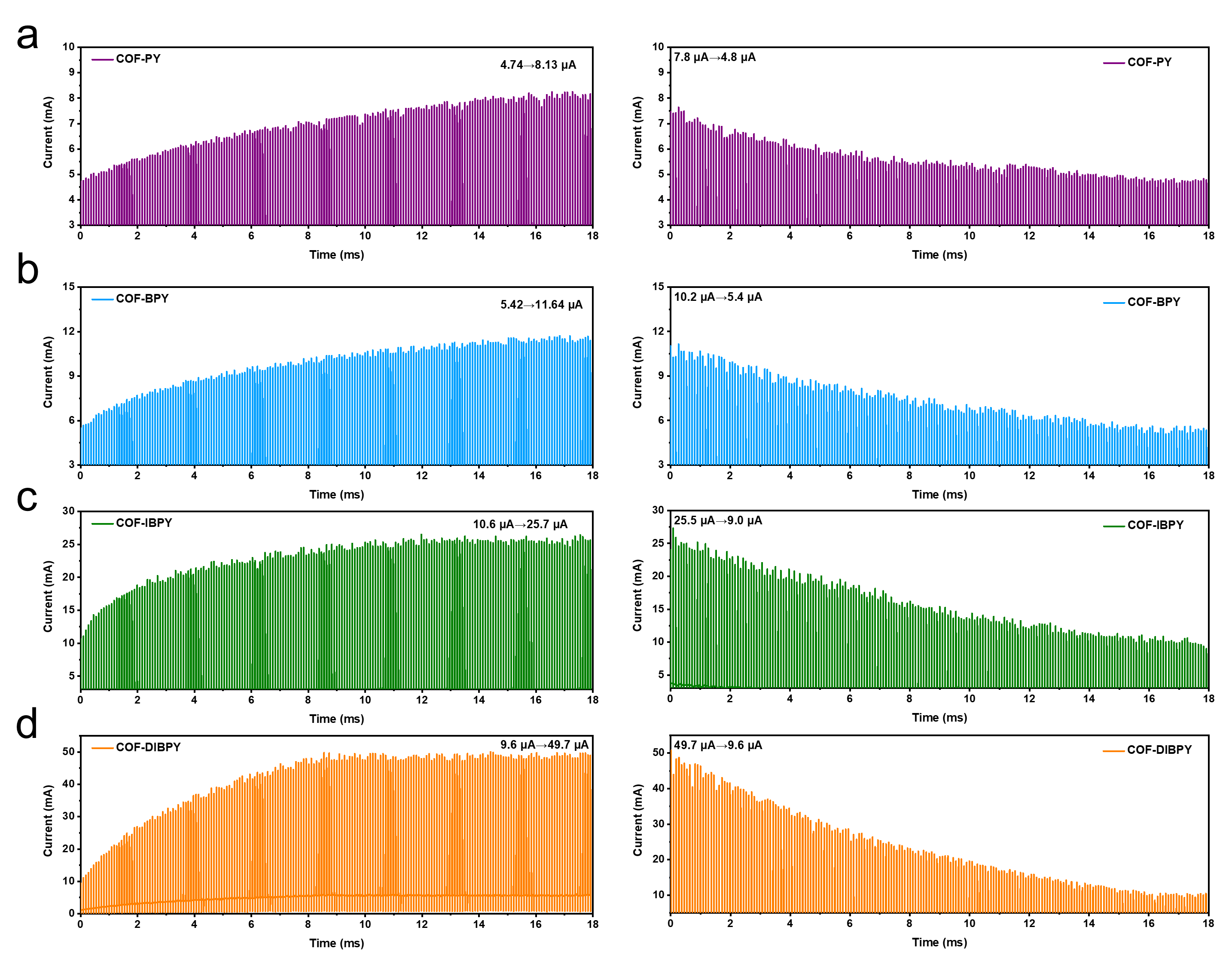


**Figure S21**. Negative voltage pulse stimulation and positive voltage pulse stimulation (pulse amplitude ±0.5 V, pulse width 3 µs) based on (a) Al/COF-PY/Au, (b) Al/COF-BPY/Au, (c) Al/COF-IBPY/Au, and (d) Al/COF-DIBPY/Au devices.


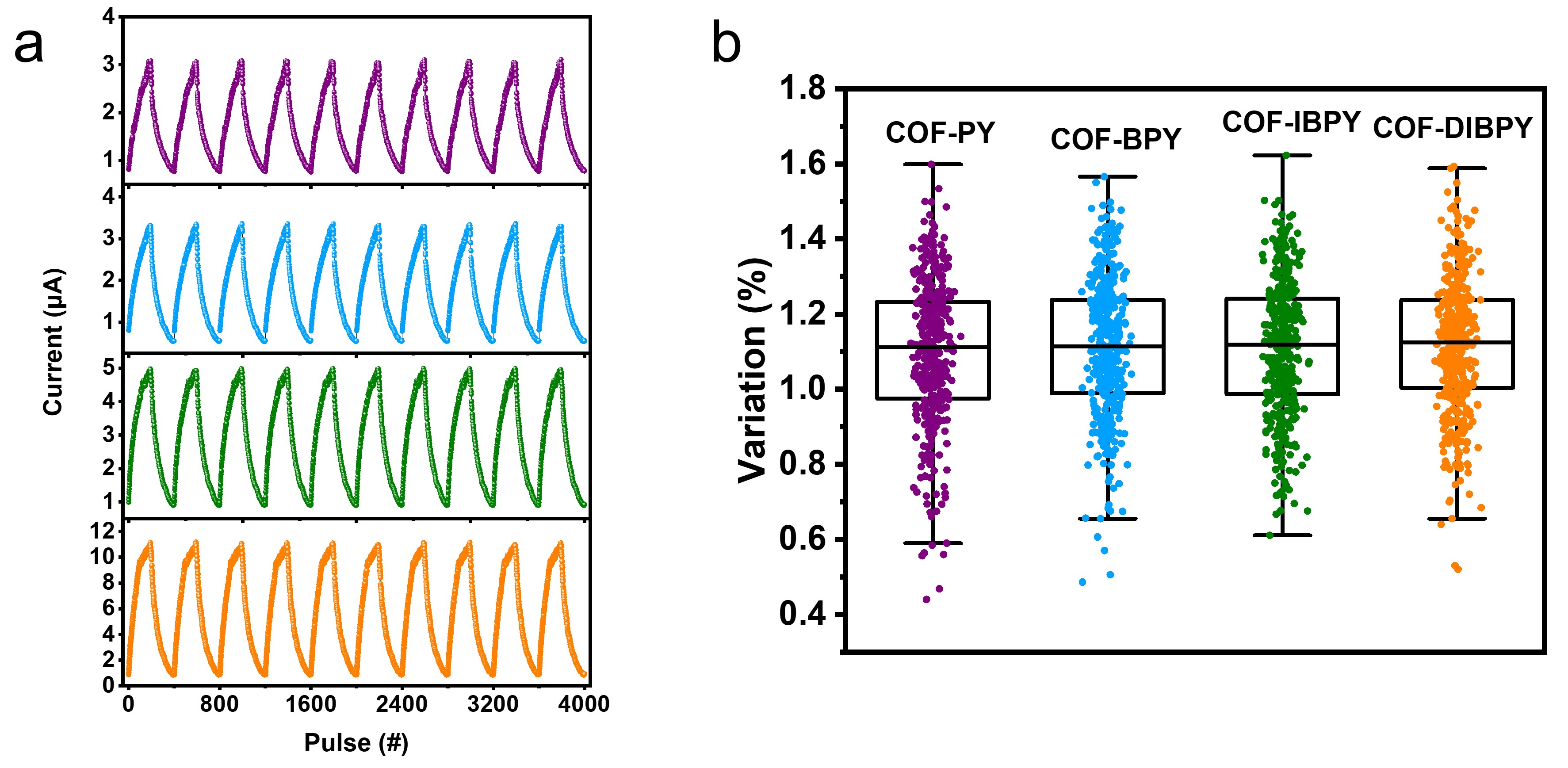


**Figure S22**. (a) Simulation of a 10-cycle repetitive process of long-term potentiation (LTP) and long-term depression (LTD) in synapses at a read voltage of 0.1 V. (b) Noise tolerance distribution of the response device under 400 pulses, calculated as the ratio of the mean to the standard deviation of the conductance state for the same number of pulses within each cycle.


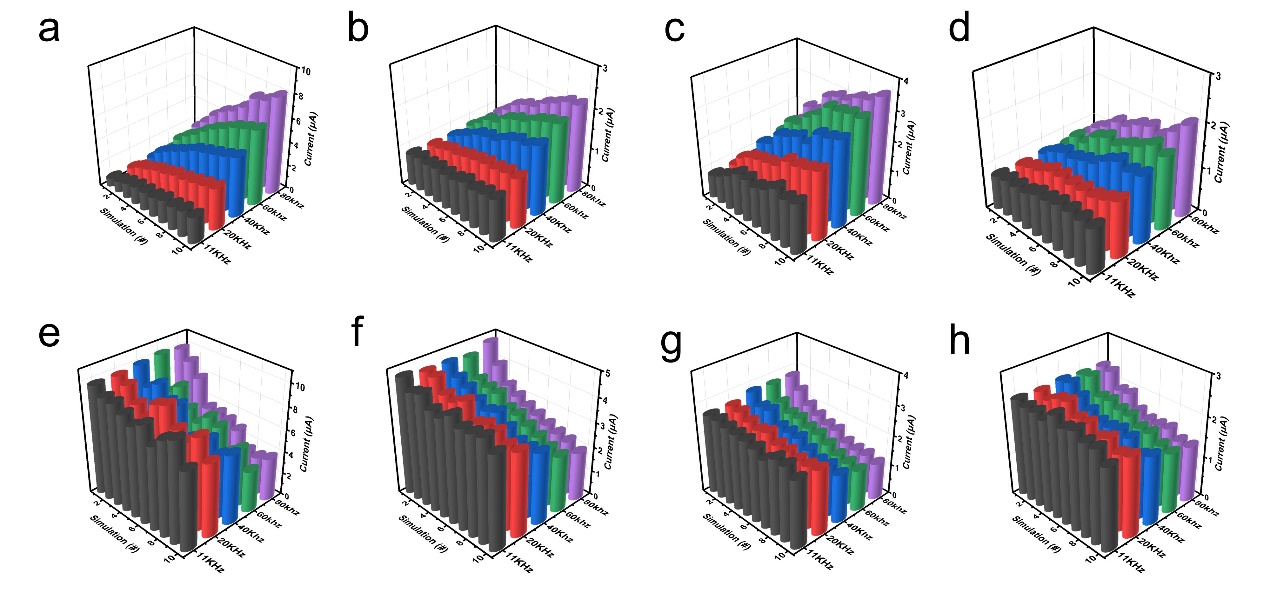


**Figure S23**. Frequency dependence of synaptic currents based on (a, e) Al/COF-DIBPY/Au, (b, f) Al/COF-IBPY/Au, (c, g) Al/COF-BPY/Au and (d, h) Al/COF-PY/Au memristors reinforce and inhibitory.


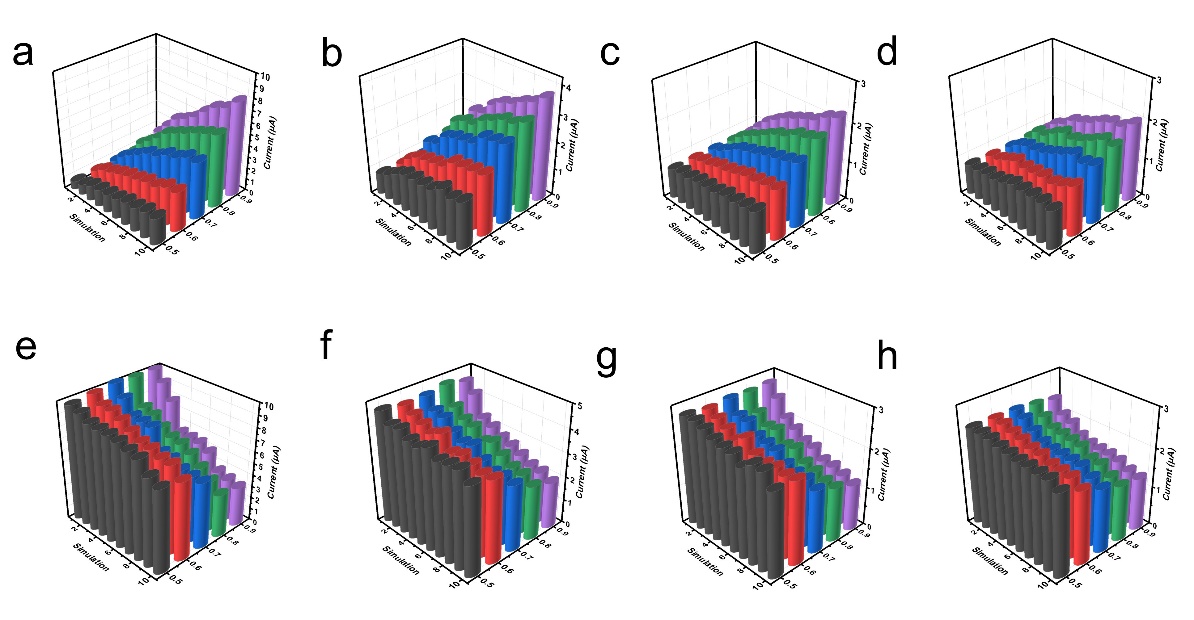


**Figure S24**. Voltage dependence of synaptic currents based on (a, e) Al/COF-DIBPY/Au, (b, f) Al/COF-IBPY/Au, (c, g) Al/COF-BPY/Au and (d, h) Al/COF-PY/Au memristors reinforce and inhibitory.


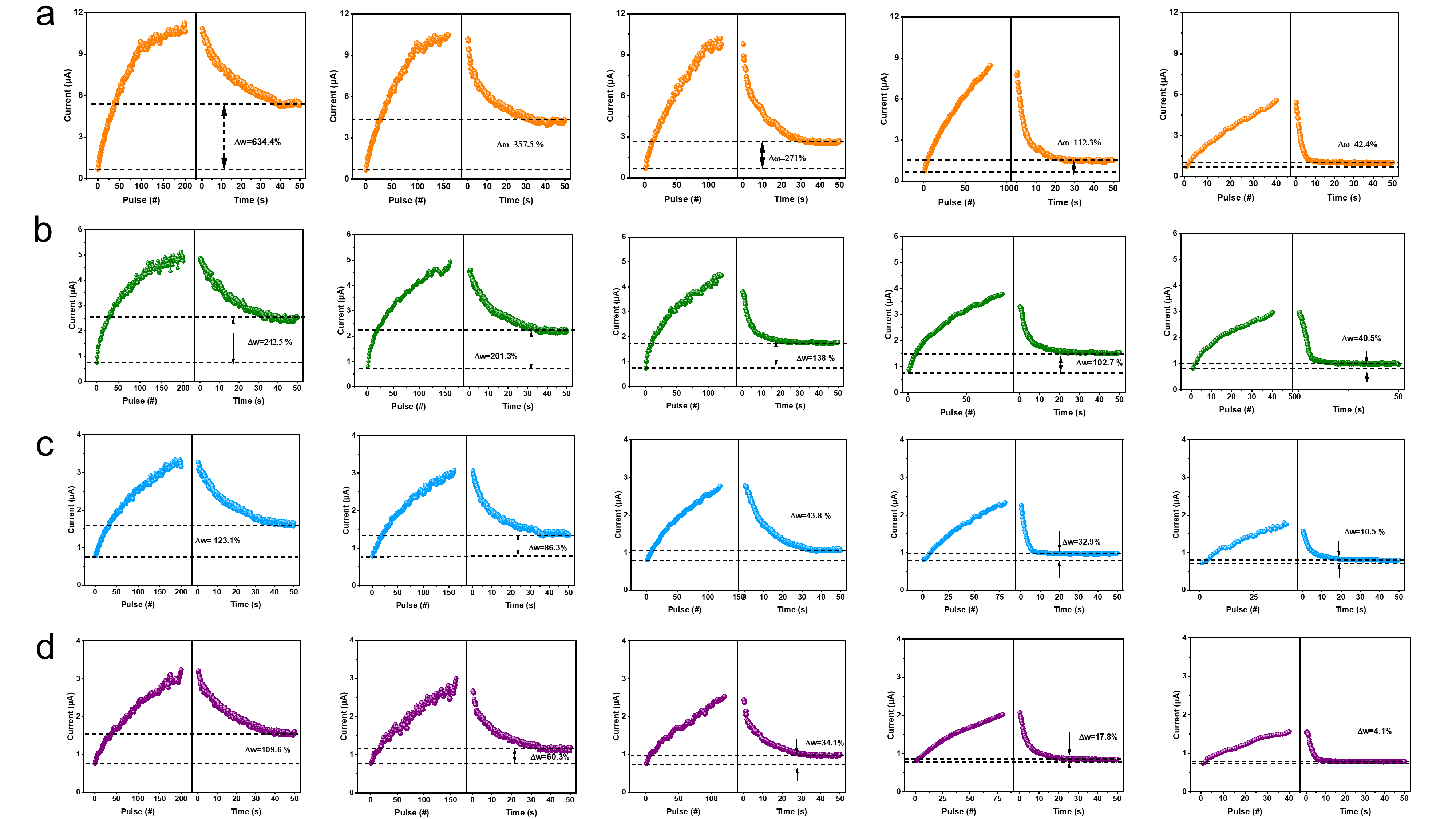


**Figure S25.** Changes in postsynaptic current in (a) Al/COF-DIBPY/Au, (b) Al/COF-IBPY/Au, (c) Al/COF-BPY/Au, and (d) Al/COF-PY/Au devices under different pulse numbers.


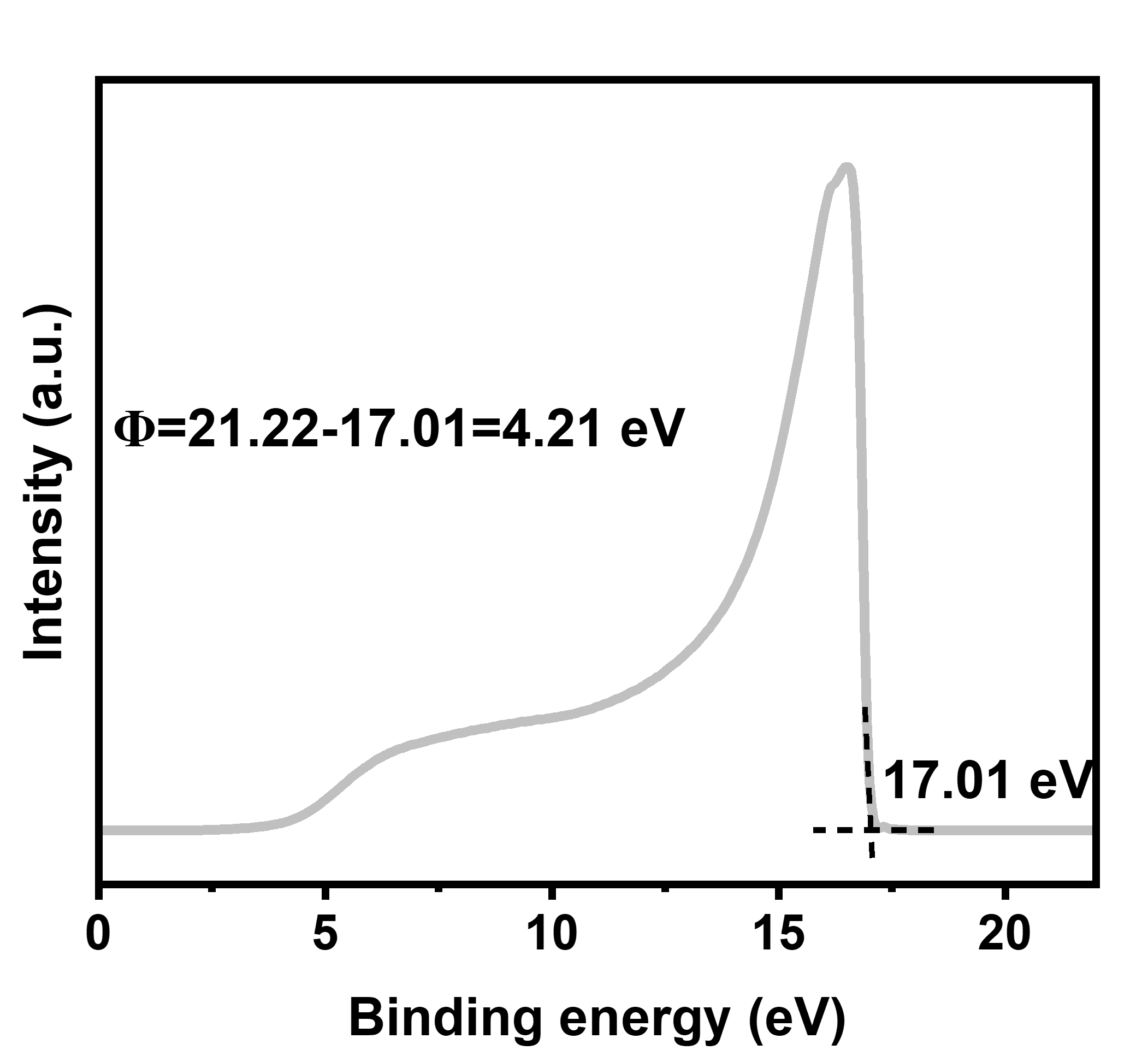


**Figure S26.** Work Function of Aluminum Based on Thermal Evaporation


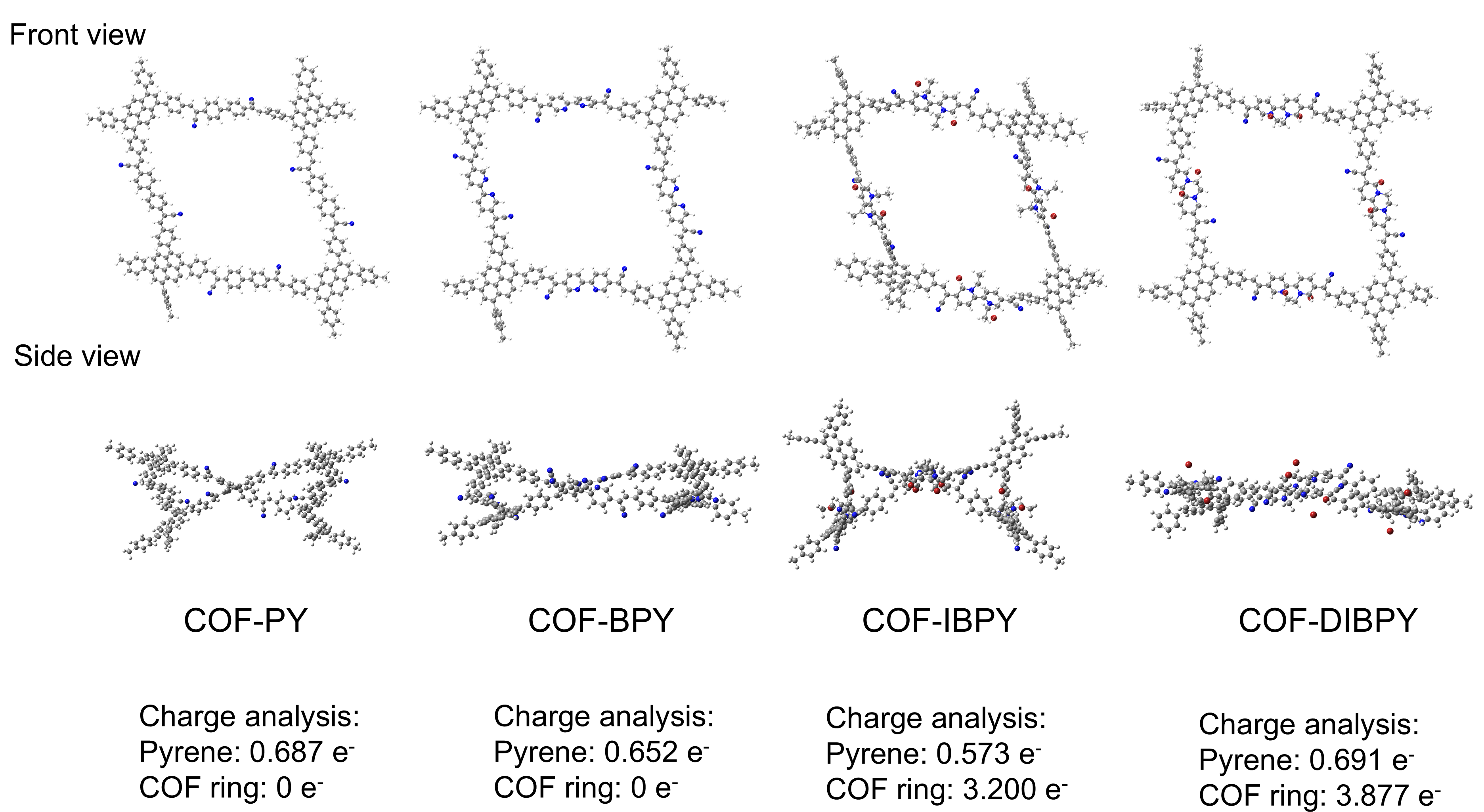


**Figure S27**. Atomic Dipole Corrected Hirshfeld charge analysis of four COFs.


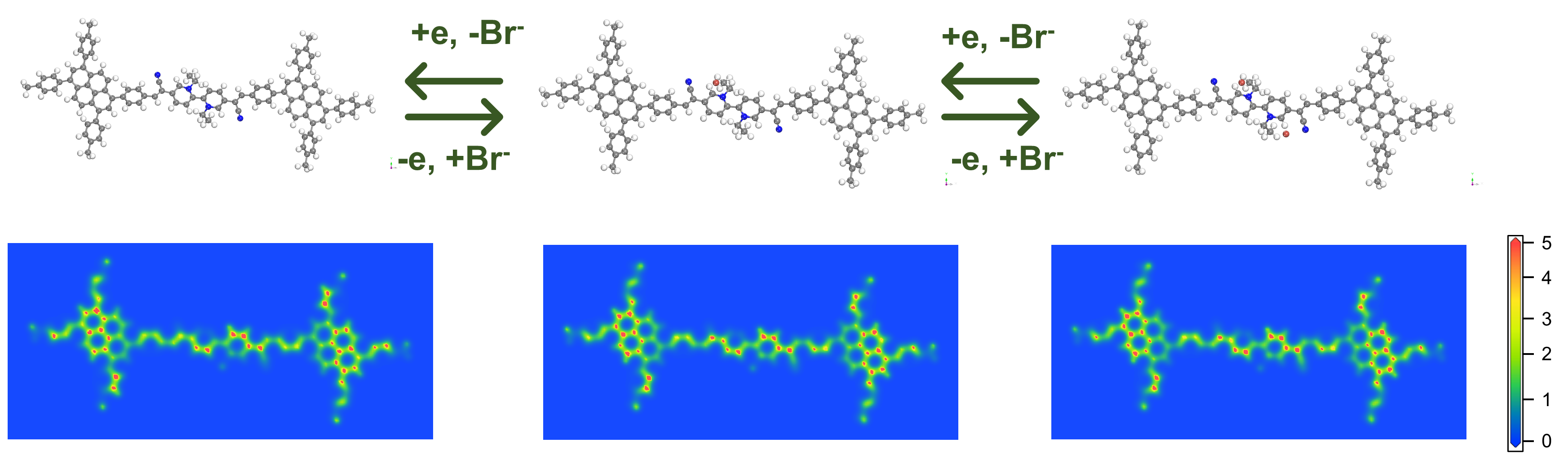


**Figure S28**. Structural evolution during redox reactions based on COF-IBPY repeating units, corresponding electron density maps.


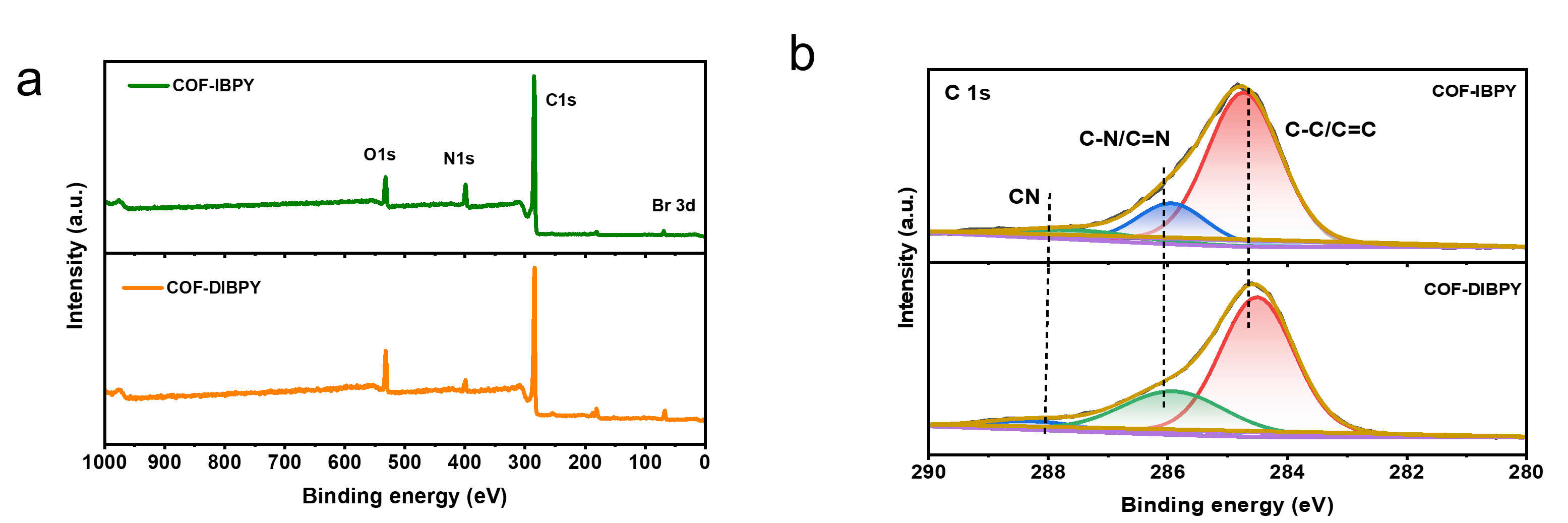


**Figure S29**. (a) XPS survey spectrum and (b) C1s XPS spectrum of two ICOFs.


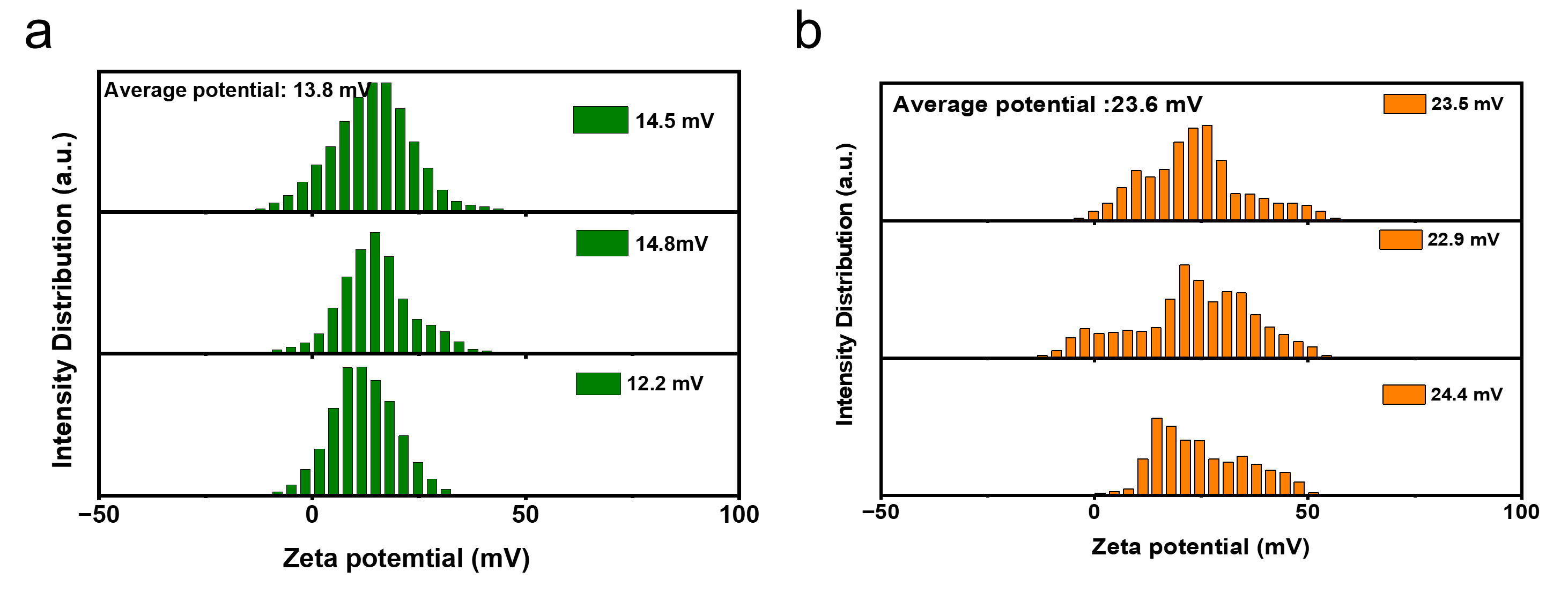


**Figure S30**. Surface zeta potential of (a) COF-IBPY and (b) COF-DIBPY.


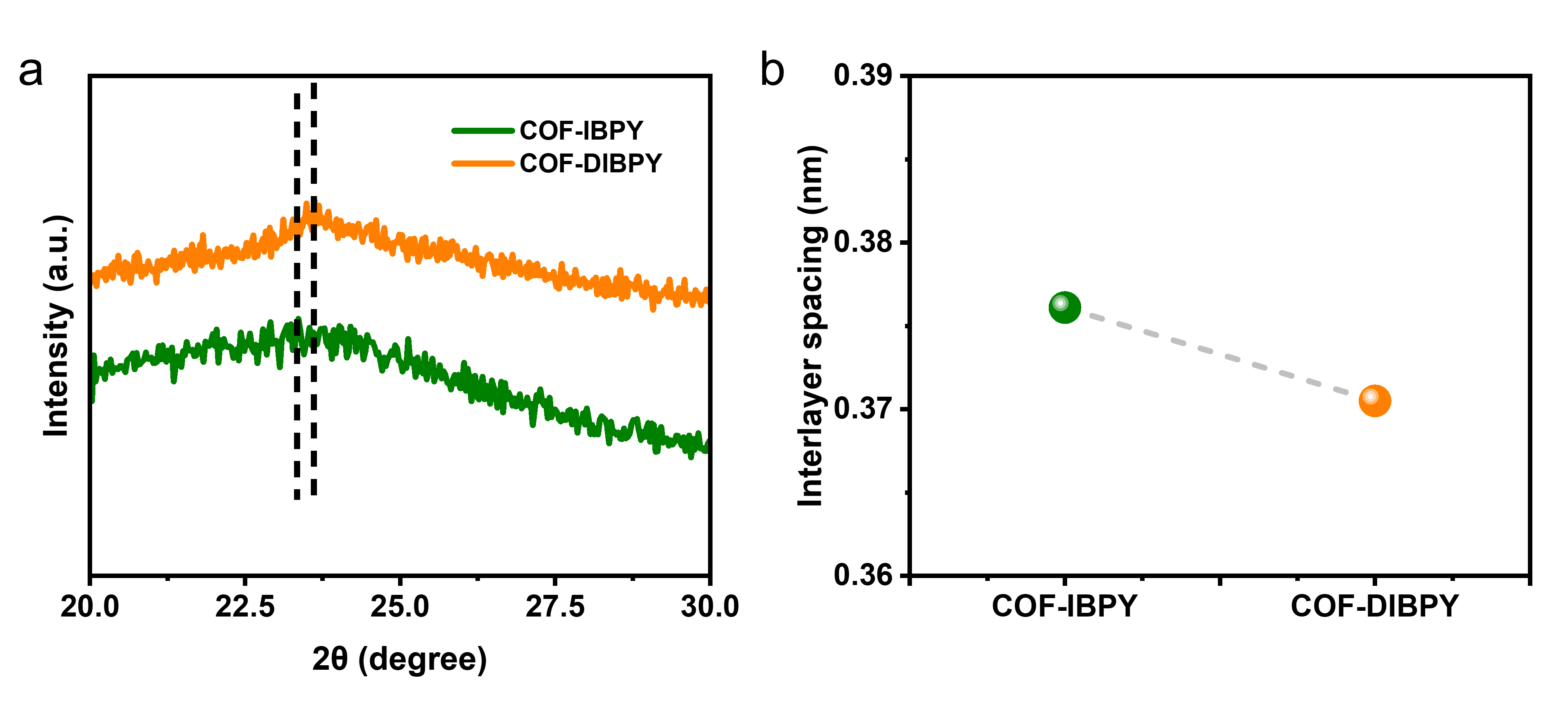


**Figure S31**. (a) X-ray diffraction pattern at the 001-crystal plane position. (b) Interlayer spacing relationships derived from XRD patterns.


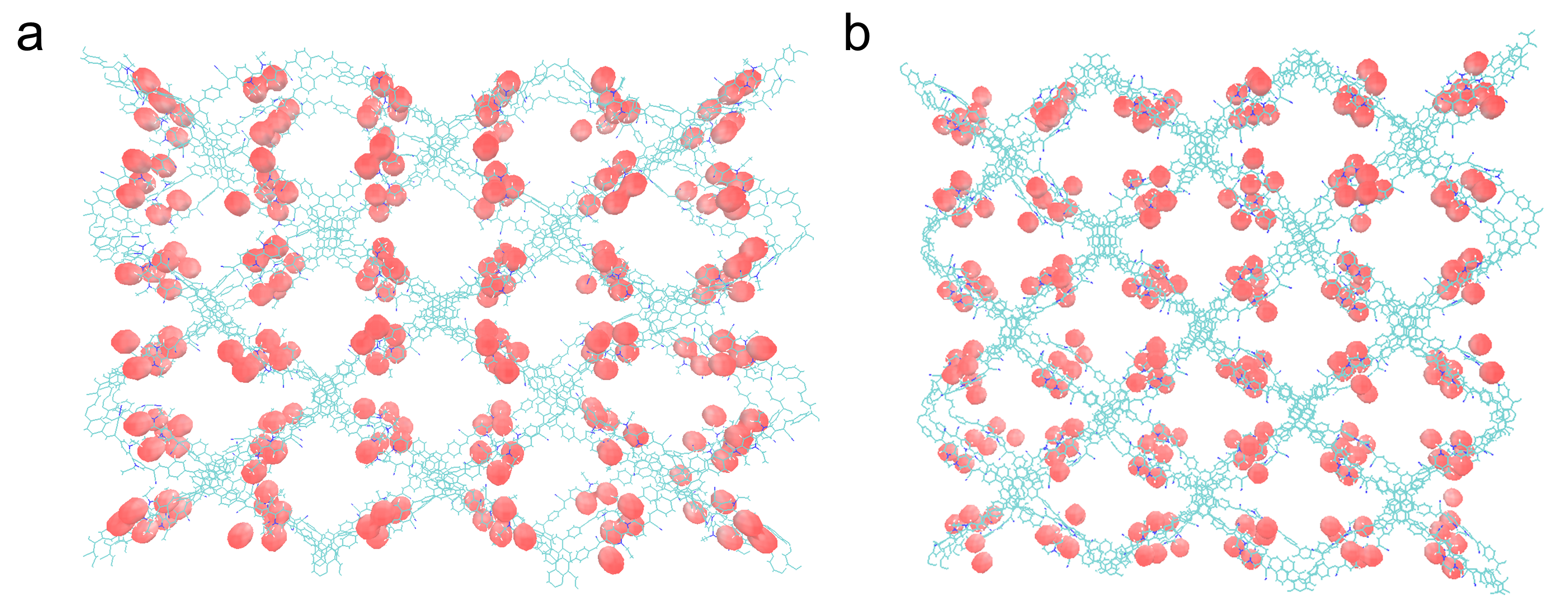


**Figure S32**. Two-dimensional density distribution of (a) COF-IBPY and (b) COF-DIBPY.

**Note**: The number density distribution serves as a vital tool in statistical mechanics for characterizing the spatial distribution of particles. By partitioning the simulated system into minute regions along a specific direction and quantifying the frequency of particle occurrence within each region, it yields a curve depicting the variation of particle density with spatial position. This distribution provides an intuitive representation of particle aggregation, dispersion, or stratification within the system, revealing microscopic insights such as solvation structures, interfacial adsorption, and channel selectivity. In molecular dynamics simulations, the number density distribution is frequently employed to analyze the distribution patterns of ions or molecules within electric fields, interfaces, or channel environments, providing quantitative evidence for understanding diffusion mechanisms and transport pathways. As evident from Figures S30(a) and S30(b), bromide anions predominantly distribute within tunnels formed by framework molecules, indicating that this ion species primarily diffuses and transfers along these tunnels.


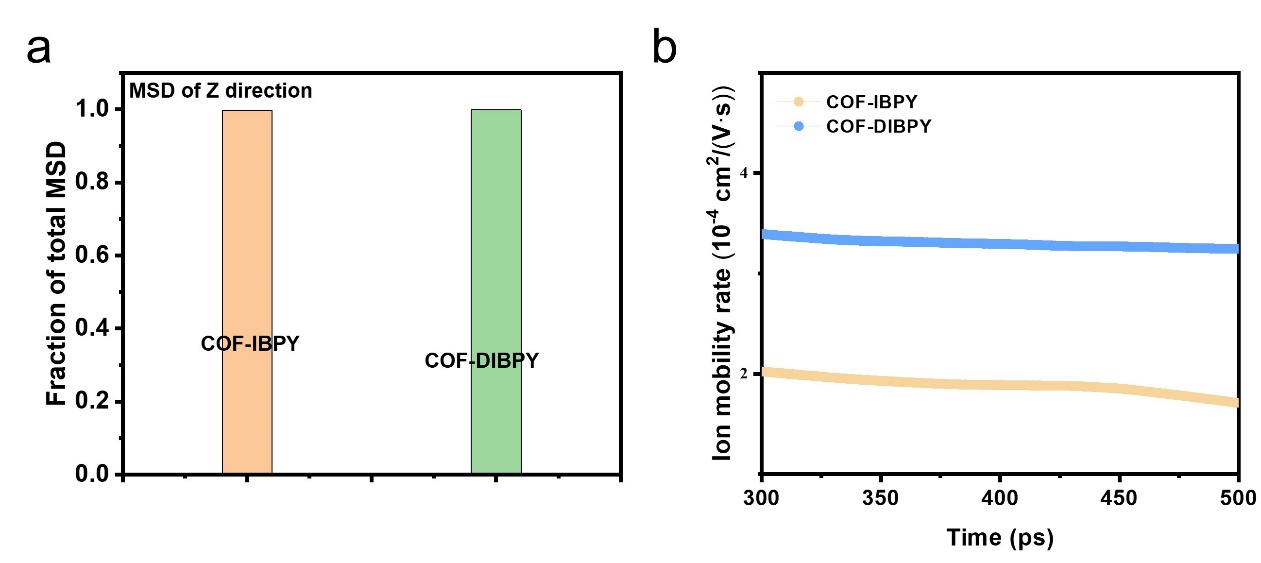


**Figure S33**. (a) The fraction of MSD in the Z-direction relative to the total MSD. (b) Data for calculating the migration rate of bromide ions under the influence of an electric field.

**Note**: To investigate the movement patterns of Br⁻ anions in an electric field, we separately quantified their diffusion data along the x, y, and z axes. At an electric field strength of 0.3 V/Å, the diffusion distance of Br⁻ anions in IBPY and DIBPY accounted for over 99.9% of the total mean square displacement (MSD). This indicates that the distance generated by electric field-driven ionic migration constitutes the primary component of the simulated MSD. The distance produced by free diffusion in the z-direction can be disregarded; it is approximated that once Br⁻ anions undergo directed migration, the entire MSD is attributable to ionic migration.


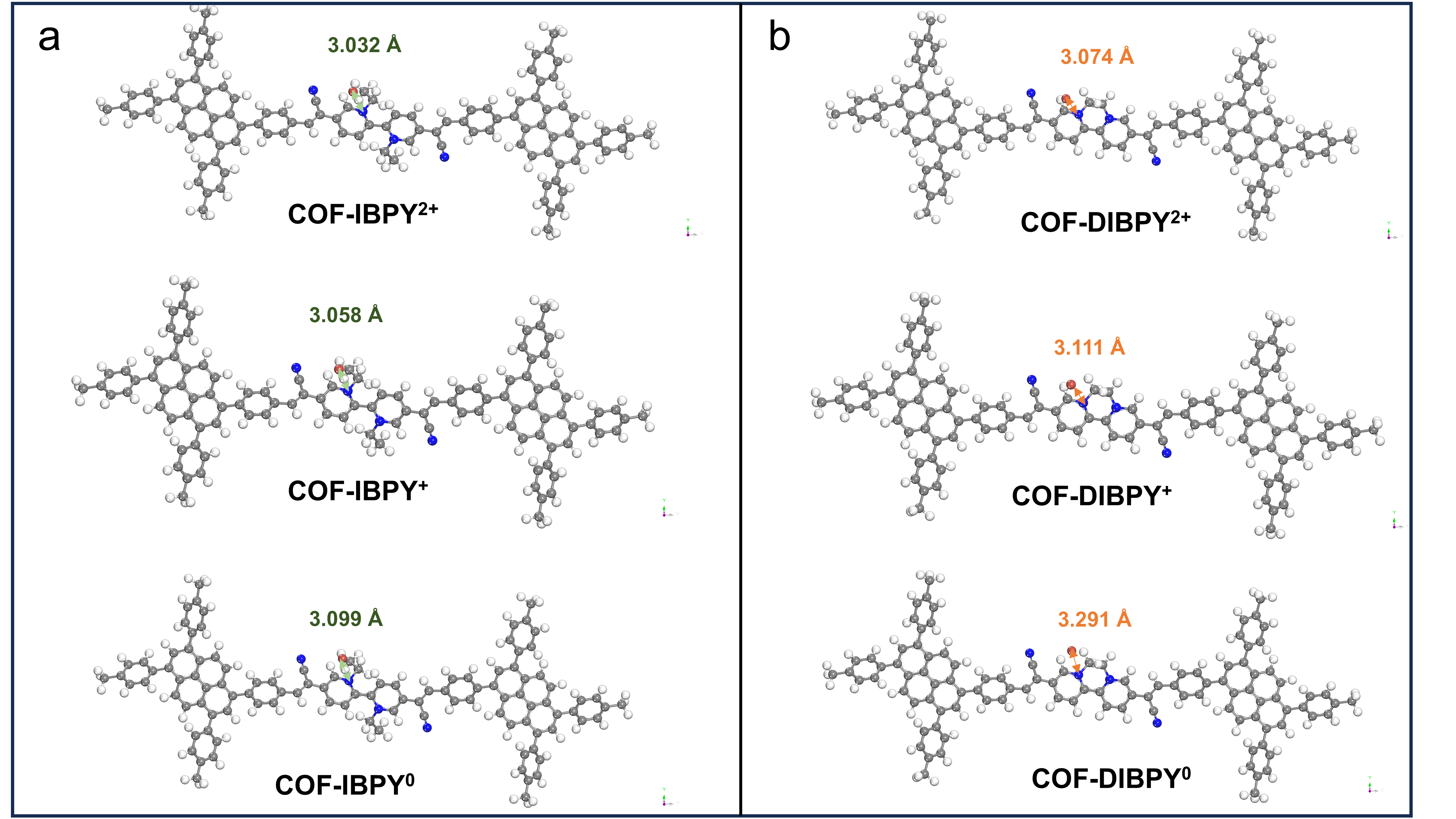


**Figure S34**. Interionic Distance in COF-IBPY and COF-DIBPY at Different Redox States.


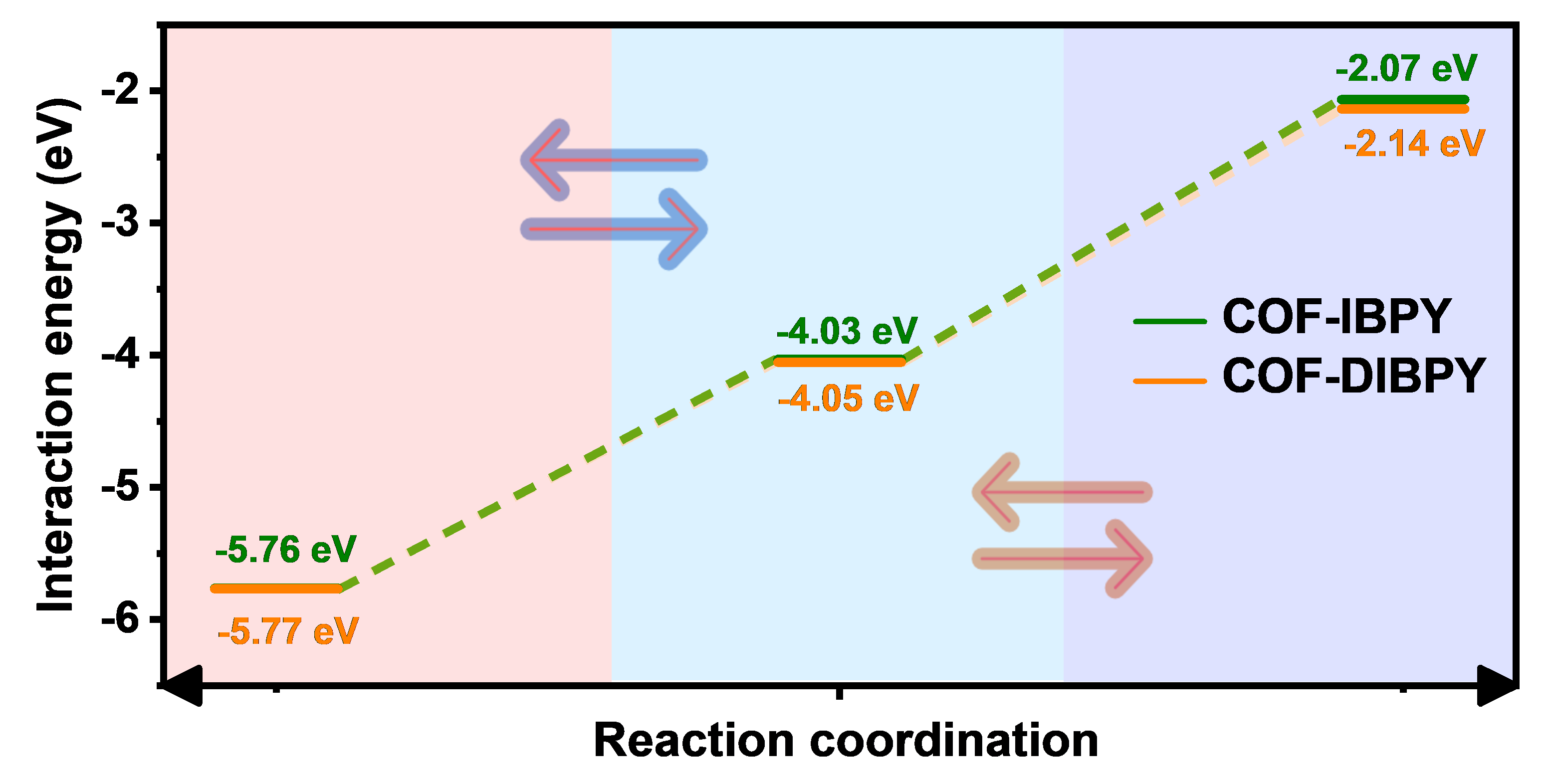


**Figure S35**. Interaction energy calculated between the faction of two COFs skeleton and Br^-^.


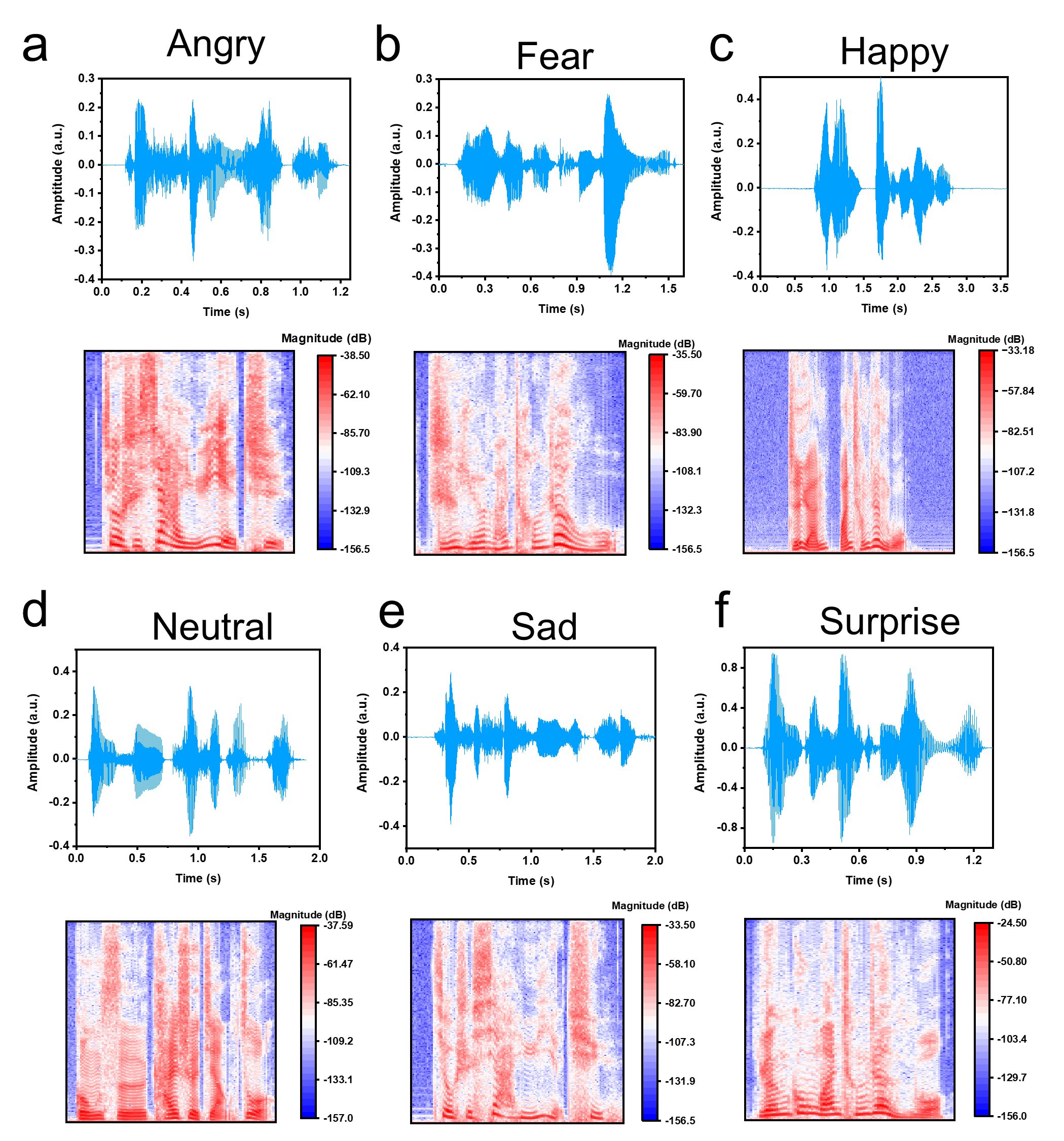


**Figure S36**. Spectral diagrams of six distinct types of emotional speech waveforms and their corresponding Mel-frequency cepstral coefficient (MFCC) spectra.


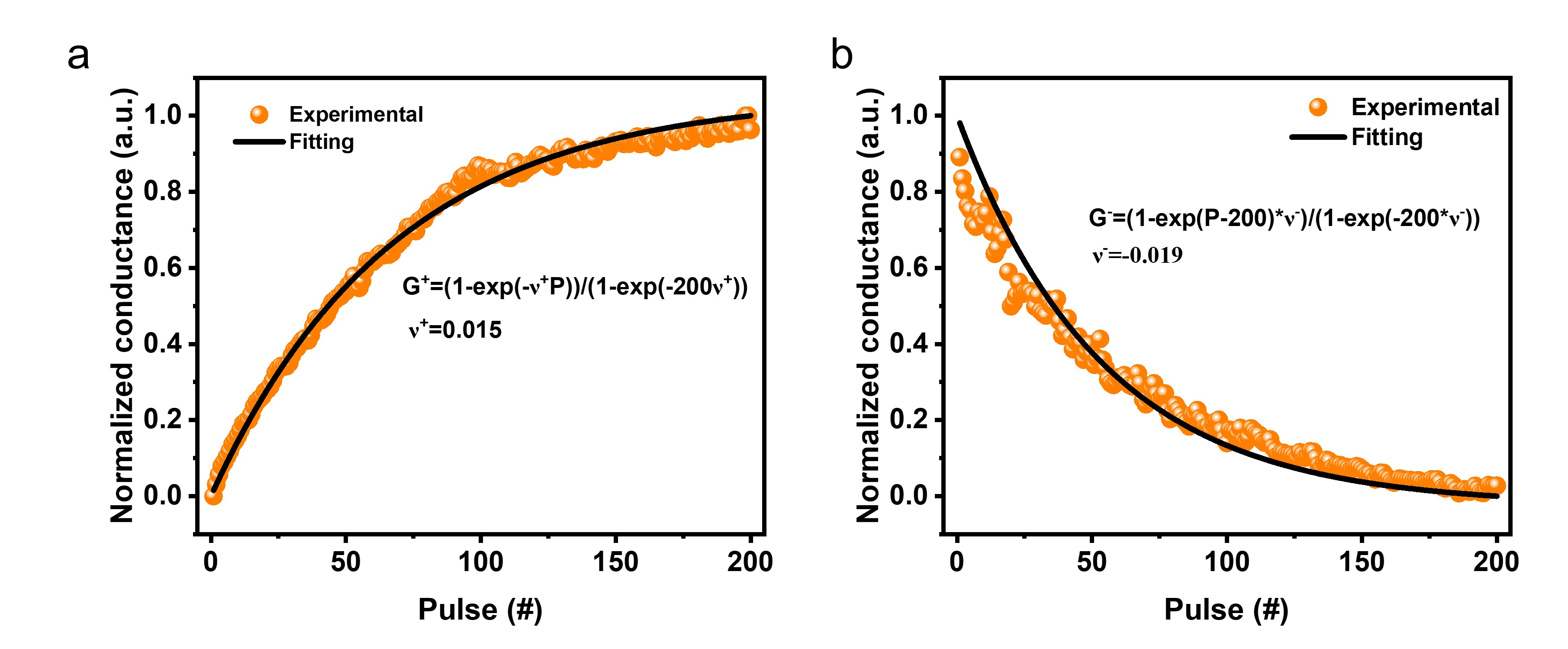


**Figure S37**. Normalized conductance for LTP and LTD in the Al/COF-DIBPY/Au device. For details on the fitted curve, see the supporting equations.

**Table S1**. Reaction conditions for quaternization of COF-BPY under different reaction conditions.

| **No.** | **Solvent** | **T (℃)** | **HAc content(ml)** | **Br^–^**  **W (%)** | **Quaternization conversion (%)** |
| --- | --- | --- | --- | --- | --- |
| 1 | Ethylene dibromide | 130 | / | / | / |
| 2 | DMF | 135 | / | / | / |
| 3 |  |  | 0.1 | / | / |
| 4 |  |  | 0.2 | / | / |
| 5 |  |  | 0.5 | 16.0 | 70.2 |
| 6 | Bromoethane | 135 | / | 16.1 | 73.5 |

**Note**: The yield of COF-DIBPY’s quaternary ammonium cyclization to diquat was monitored by adjusting the type of solvent and protonation concentration, as shown in Figure S1 (with conditions indicated by the corresponding numbers). The bromide ion content in COF-IBPY and COF-DIBPY was determined by ICP-MS to calculate the grafting yield in the products. The theoretical bromide ion contents of COF-IBPY and COF-DIBPY were 21.9% and 22.8% (w), respectively; the quaternary ammonium conversion yield was calculated based on the experimentally measured ion content.

**Table S2**. The related lattice parameters of four COFs.

|  | **COF-PY** | **COF-BPY** | **COF-IBPY** | **COF-DIBPY** |
| --- | --- | --- | --- | --- |
| **Crystal system** | Triclinic | Triclinic | Triclinic | Triclinic |
| **Space group** | P1 | P1 | P1 | P1 |
| **a (Å)** | 42.8223 | 42.1268 | 42.9908 | 42.8910 |
| **b (Å)** | 37.9980 | 42.1268 | 37.9018 | 37.6669 |
| **c (Å)** | 3.8578 | 3.7949 | 3.6844 | 3.5907 |
| **α (°)** | 90 | 90 | 90 | 90 |
| **β (°)** | 90 | 90 | 90 | 90 |
| **γ (°)** | 90 | 90 | 90 | 90 |
|  |  |  |  |  |
|  |  |  |  |  |

**Table S3.** Band properties of four COFs calculated using UPS in combination with the Tau-c method.

|  | **VBM (eV)** | **CBM (eV)** | **Φ_S_ (eV)** | **E_F_ (eV)** | **Φ_Al_-Φ_S_** |
| --- | --- | --- | --- | --- | --- |
| COF-PY | -4.16 | -1.98 | 3.11 | -3.11 | 1.1 |
| COF-BPY | -4.20 | -2.00 | 3.57 | -3.57 | 0.64 |
| COF-IBPY | -4.73 | -2.60 | 3.60 | -3.60 | 0.61 |
| COF-DIBPY | -5.01 | -3.06 | 3.84 | -3.84 | 0.37 |

**Note**: Valence Band Maximum (VBM), the work function (Φ_S_), and the Fermi level (E_F_) were calculated using UPS; CBM was obtained by plotting using the tau-c method. For detailed calculation formulas, see the **supporting equations**.

**Table S4.** Comparison with the performance of memristors recently reported.

| No. | Drving voltage(V) | Current variation | Frequency (Hz) | Pulse width | Materials | Ref. |  |
| --- | --- | --- | --- | --- | --- | --- | --- |
| 1 | 0.5 | ~0.7-11 μA | 11000 | 3 μs | COF-DIBPY | This work |  |
| 2 | 2 | ~0.3-0.8 mA | 100 | 1 ms | PTDZ-DPP | [6] |  |
| 3 | 7 | ~300-1100 μA | 8 | 1.72 s | ClCuPc | [7] |  |
| 4 | 8 | 1.0-2.5 mA | 10 | 0.1 s | MDMO-PPV | [8] |  |
| 5 | 0.5 | 150-210μA | - | 1 μs | TpDb | [9] |  |
| 6 | 0.3 | 68-82 pA | 29411 | 100 ms | TPPS | [10] |  |
| 7 | 1 | 5-75 μA | - | 20 ms | COF-Azu | [11] |  |
| 8 | 0.3 | 0.5-2.25 mA | - | - | N-GOQD | [12] | |
| 9 | 5 | 1.5-15 nA | 1000000 | - | APP | [13] |  |
| 10 | 1 | 5-25μA | 3 | 0.33 s | C-OIHP | [14] |  |
| 11 | 1 | 80-300μA | 77000 | 10 μs | Ta-Cu_3_ COF | [15] |  |
| 12 | 1.85 | 85-97.0 μA | 2000000 | 500 ns | HOF-FJU-52 | [16] |  |
| 13 | 0.04 | 0.05-1 | - | 100ms | Zn-TCPP | [17] |  |
| 14 | 1 | 0.1-2 μA | - | 1 ms | F-2DP | [18] |  |
| 15 | - | 0.45-1.2 mA | - | - | COF_TP-TD_ | [19] |  |
| 16 | 1 | 0.3-0.6 μA | - | 5 ms | polyimide micropores | [20] |  |
| 17 | 0.8 | - | / | / | TT-COF | [21] |  |
| 18 | 2 | - | - | 0.02 | 1D H-Py-BT COF | [22] |  |

**Table S5.** Convolutional Neural Network (CNN) Training Parameters.

| No. | Name | Type | Activate | Learnable parameter attributes |
| --- | --- | --- | --- | --- |
| 1 | 224*224*3 image | Image input | 224*224*3*1 | - |
| 2 | 4 5*5  Stride Length [1 1] | Two-dimensional convolution | 220*220*4*1 | Weight 5*5*3*4  Bias 1*1*4 |
| 3 | ReLU | ReLU | 220*220*4*1 | - |
| 4 | Norm 1 | Batch normalization | 220*220*4*1 | Offset 1*1*4  Scale 1*1*4 |
| 5 | 5*5  Stride Length [3 3] | Max pooling | 72*72*4*1 | - |
| 6 | 16 3*3  Stride Length [1 1] | Two-dimensional convolution | 70*70*16*1 | Weight 3*3*4*16  Bias 1*1*16 |
| 7 | ReLU | ReLU | 70*70*16*1 | - |
| 8 | Norm 2 | Batch normalization | 70*70*16*1 | Offset 1*1*16  Scale 1*1*16 |
| 9 | 5*5  Stride Length [3 3] | Max pooling | 22*22*16*1 | - |
| 10 | 8 3*3  Stride Length [1 1] | Two-dimensional convolution | 20*20*8*1 | Weight 3*3*16*8  Bias 1*1*8 |
| 11 | ReLU | ReLU | 20*20*8*1 | - |
| 12 | Norm 3 | Batch normalization | 20*20*8*1 | Offset 1*1*8  Scale 1*1*8 |
| 13 | 3*3  Stride Length [2 2] | Max pooling | 9*9*8*1 | - |
| 14 | Flatten | Flatten | 648*1 | - |
| 15 | LSTM: 50 hidden neurons | LSTM | 50*1 | Input weights 200*648  Recurrent weights 200*50  Bias 200*1 |
| 16 | Fully connected layer 6 | Fully connected layer | 6*1 | Weights 6*50  Bias 6*1 |
| 17 | Soft max | Soft max | 6*1 | - |
| 18 | Class output | Class output | 6*1 | - |

**Reference**

1. C. Jun, W. Yuting, Z. Wei, Z. Jie, W. Lei, Z. Meng, F. Fengtao, W. Xiaojun, X. Hangxun, *Adv. Mater.* **2023**, 36, 2305313.
2. H. X. Li, Q. X. Li, F. Z. Li, J. P. Liu, G. D. Gong, Y. Q. Zhang, Y. B. Leng, T. Sun, Y. Zhou, S. T. Han, *Adv. Mater.* **2023**, 36, 2308153.
3. X. Li, G. Jiang, M. Jian, C. Zhao, J. Hou, A. W. Thornton, X. Zhang, J. Z. Liu, B. D. Freeman, H. Wang, L. Jiang, H. Zhang, *Nat. Commun.* **2023**, 14, 286.
4. X. Dai, Q. Hua, C. Jiang, Y. Long, Z. Dong, Y. Shi, T. Huang, H. Li, H. Meng, Y. Yang, R. Wei, G. Shen, W. Hu, *Nano Energy* **2024**, 124, 109473.
5. Y. Tao, H. Liu, H. Y. Kong, T. X. Wang, H. Sun, Y. J. Li, X. Ding, L. Sun, B. H. Han, *Angew. Chem. Int. Ed. Engl.* **2022**, 61, e202205796.
6. Z. Lv, M. H. Jiang, H. Y. Liu, Q. X. Li, T. Xie, J. Yang, Y. Wang, Y. Zhai, G. Ding, S. Zhu, J. H. Li, M. Zhang, Y. Zhou, B. Tian, W. Y. Wong, S. T. Han, *Adv. Funct. Mater.* **2025**, 35, 2424382.
7. J. Zhou, W. Li, Y. Chen, Y. H. Lin, M. Yi, J. Li, Y. Qian, Y. Guo, K. Cao, L. Xie, H. Ling, Z. Ren, J. Xu, J. Zhu, S. Yan, W. Huang, *Adv. Mater.* **2020**, 33, 2006201.
8. J. Li, Y. Qian, W. Li, S. Yu, Y. Ke, H. Qian, Y. H. Lin, C. H. Hou, J. J. Shyue, J. Zhou, Y. Chen, J. Xu, J. Zhu, M. Yi, W. Huang, *Adv. Mater.* **2023**, 35, e2209728.
9. P. Pal, H. Li, R. Al‐Ajeil, A. K. Mohammed, A. Rezk, G. Melinte, A. Nayfeh, D. Shetty, N. El‐Atab, *Adv. Sci.* **2024**, 11.2408648.
10. S. Liu, Z. He, B. Zhang, X. Zhong, B. Guo, W. Chen, H. Duan, Y. Tong, H. He, Y. Chen, G. Liu, *Adv. Sci.* **2023**, 10, 2305075.
11. Z. Zhao, M. E. El‐Khouly, Q. Che, F. Sun, B. Zhang, H. He, Y. Chen, *Angew. Chem. Int. Ed.* **2023**, 62, e202217249.
12. S. Sokolov, M. Ali, R. Riaz, Y. Abbas, M. J. Ko, C. Choi, *Adv. Funct. Mater.* **2019**, 29,1807504.
13. Y. Y. Zhao, W. J. Sun, J. Wang, J. H. He, H. Li, Q. F. Xu, N. J. Li, D. Y. Chen, J. M. Lu, *Adv. Funct. Mater.* **2020**, 30, 2004245.
14. H. Kim, K. Lee, G. Zan, E. Shin, W. Kim, K. Zhao, G. Jang, J. Moon, C. Park, *ACS Nano* **2024**, 19, 691-703.
15. Q. Zhang, Q. Che, D. Wu, Y. Zhao, Y. Chen, F. Xuan, B. Zhang, *Angew. Chem. Int. Ed. Engl.* **2024**, 136, e202413311.
16. S. Chen, Y. Ju, Y. Yang, F. Xiang, Z. Yao, H. Zhang, Y. Li, Y. Zhang, S. Xiang, B. Chen, Z. Zhang, *Nat. Commun.* **2024**, 15, 298.
17. Y. Wang, J. Su, G. Ouyang, S. Geng, M. Ren, W. Pan, J. Bian, M. Cao, *Adv. Funct. Mater.* **2024**, 34, 397.
18. L. Liu, W. Ji, W. He, Y. Cheng, R. Hao, P. Hao, H. Dong, X. Ding, S. Lei, B. Han, W. Hu, *Adv. Mater.* **2024**, 36,2405328.
19. D. Huo, Z. Gu, B. Song, Y. Yu, M. Wang, L. Qin, H. Li, D. Ouyang, S. Xiao, W. Hu, J. Wu, Y. Li, X. Chi, T. Zhai, *IM.* **2025**, 4, 515.
20. G. Guo, T. Xiong, B. Xie, J. Zhang, J. Zhang, Y. Lu, Y. Zhao, W. Ma, C. Pan, Y. Jiang, L. Mao, J. Wang, P. Yu, *Nat Commun* **2026**, DOI: 10.1038/s41467-026-70728-1.
21. Y. Tao, D. Li, T. Hu, S. Qin, Z. Wan, S. Zhang, K. Cao, Y. Rao, G. Ma, J. Zhang, H. Wang, C. Chen, H. Wan, H. Wang, *Journal of Materials Chemistry C* **2025**, 13, 6444.
22. Y. Huang, W. Yang, P. K. Zhou, N. Liu, S. Xu, J. Qiu, T. Zeng, C. Wu, W. Huang, W. Lin, X. Chen, *Adv. Funct. Mater.* **2025**, 35, 2505890.
